# Supplementary figures and images for: A CRISPR-Cas9 screen reveals genetic determinants of the cellular response to decitabine (part 1 of 2)
Source: EMBO Rep. 2025 Feb 10;26(6):1528–65. doi: 10.1038/s44319-025-00385-w (PMC11933316; doi:10.1038/s44319-025-00385-w)

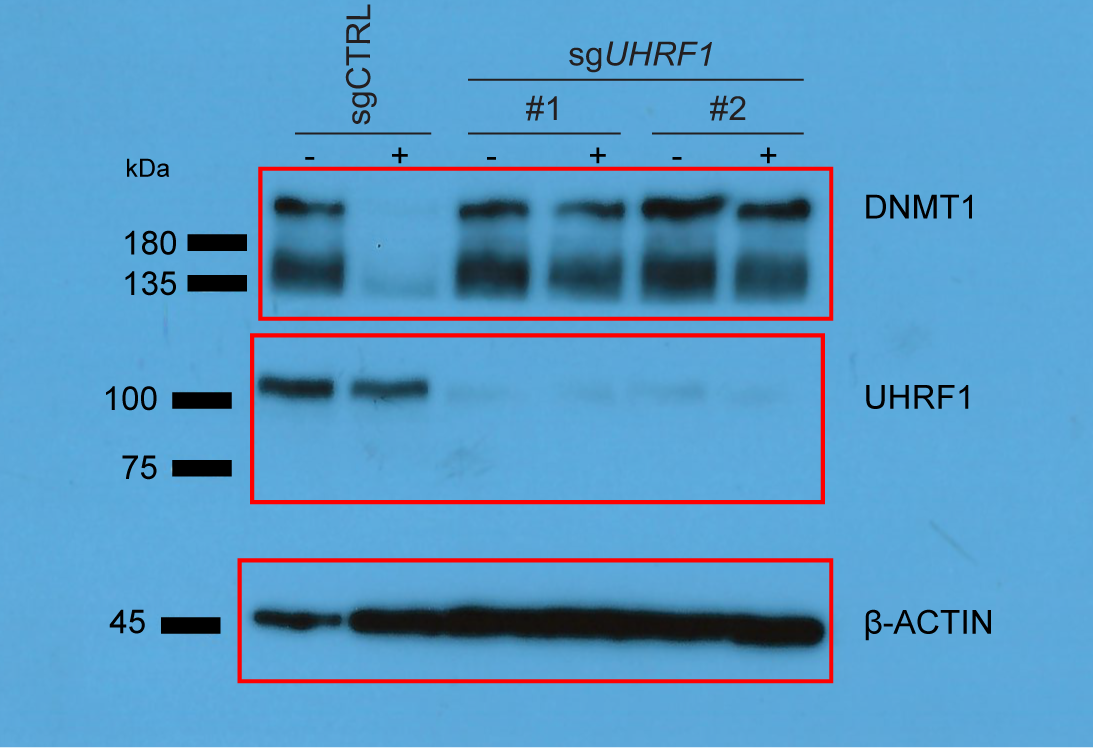

Supplement: Supplementary file 7 — Source data Fig. 3 [file 44319_2025_385_MOESM7_ESM.zip › Figure 3/3C/Western-DNMT1-UHRF1-ACTB.tif]

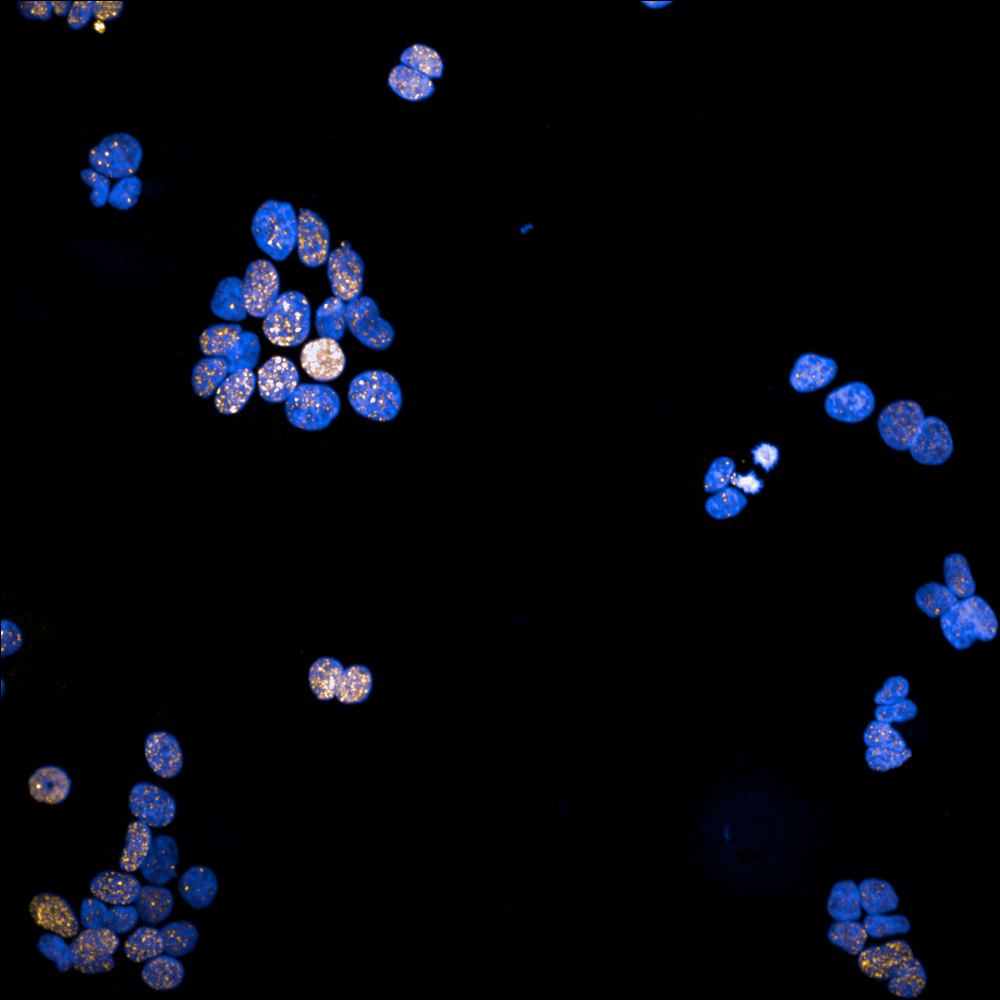

Supplement: Supplementary file 7 — Source data Fig. 3 [file 44319_2025_385_MOESM7_ESM.zip › Figure 3/3D/sgCTRL-DAC.png]

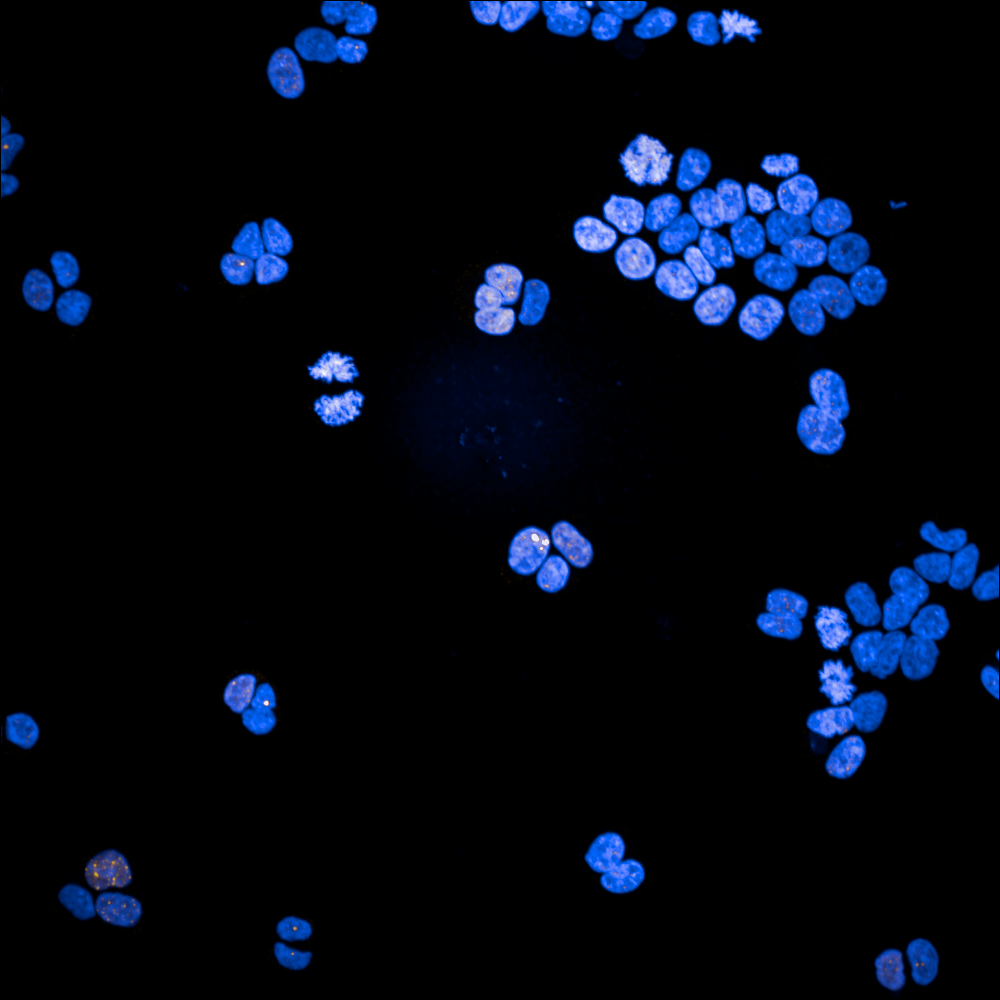

Supplement: Supplementary file 7 — Source data Fig. 3 [file 44319_2025_385_MOESM7_ESM.zip › Figure 3/3D/sgCTRL-DMSO.png]

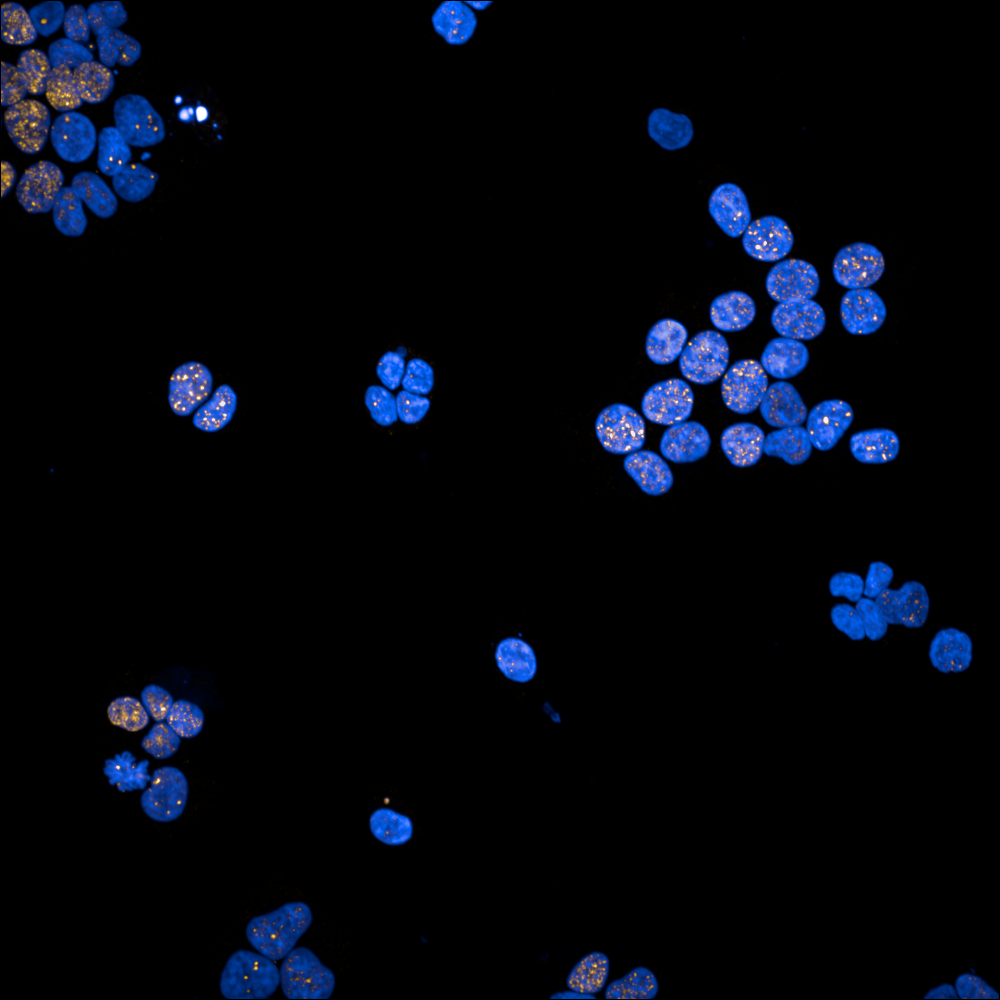

Supplement: Supplementary file 7 — Source data Fig. 3 [file 44319_2025_385_MOESM7_ESM.zip › Figure 3/3D/sgDNMT1#1-DAC.png]

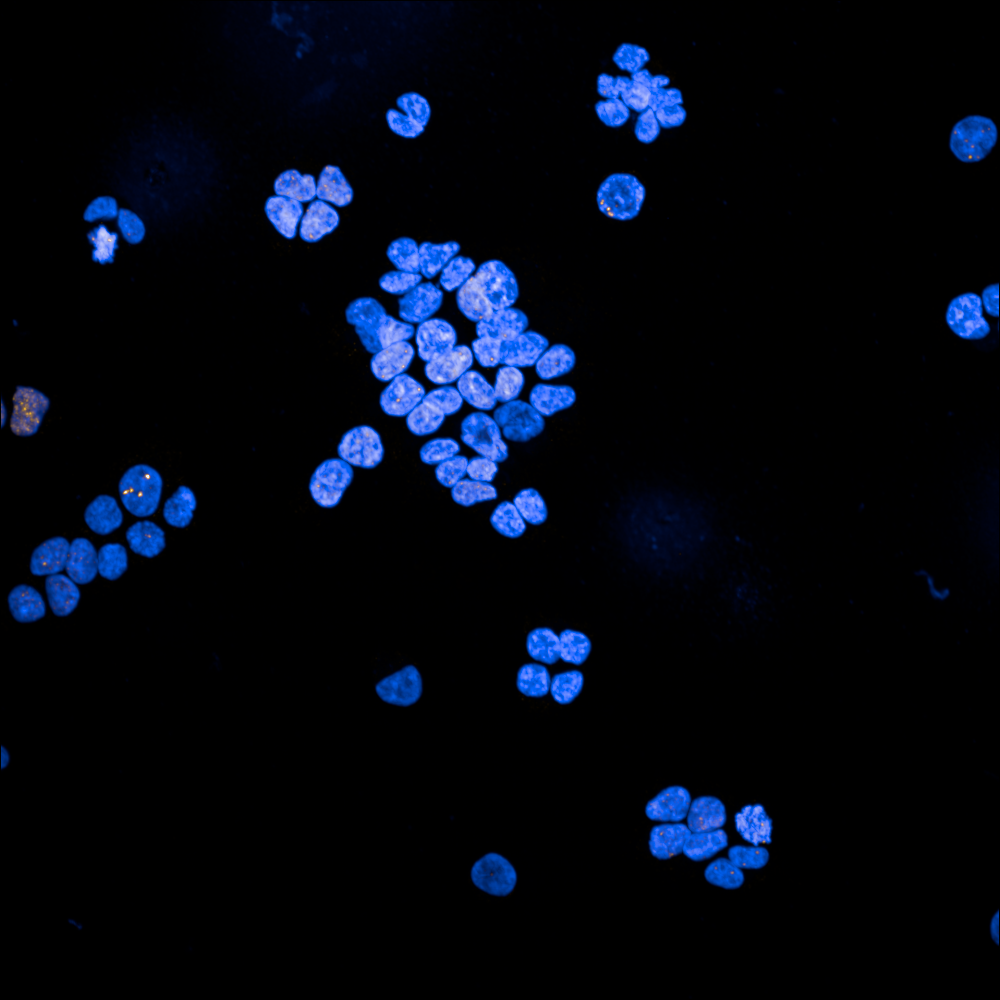

Supplement: Supplementary file 7 — Source data Fig. 3 [file 44319_2025_385_MOESM7_ESM.zip › Figure 3/3D/sgDNMT1#1-DMSO.png]

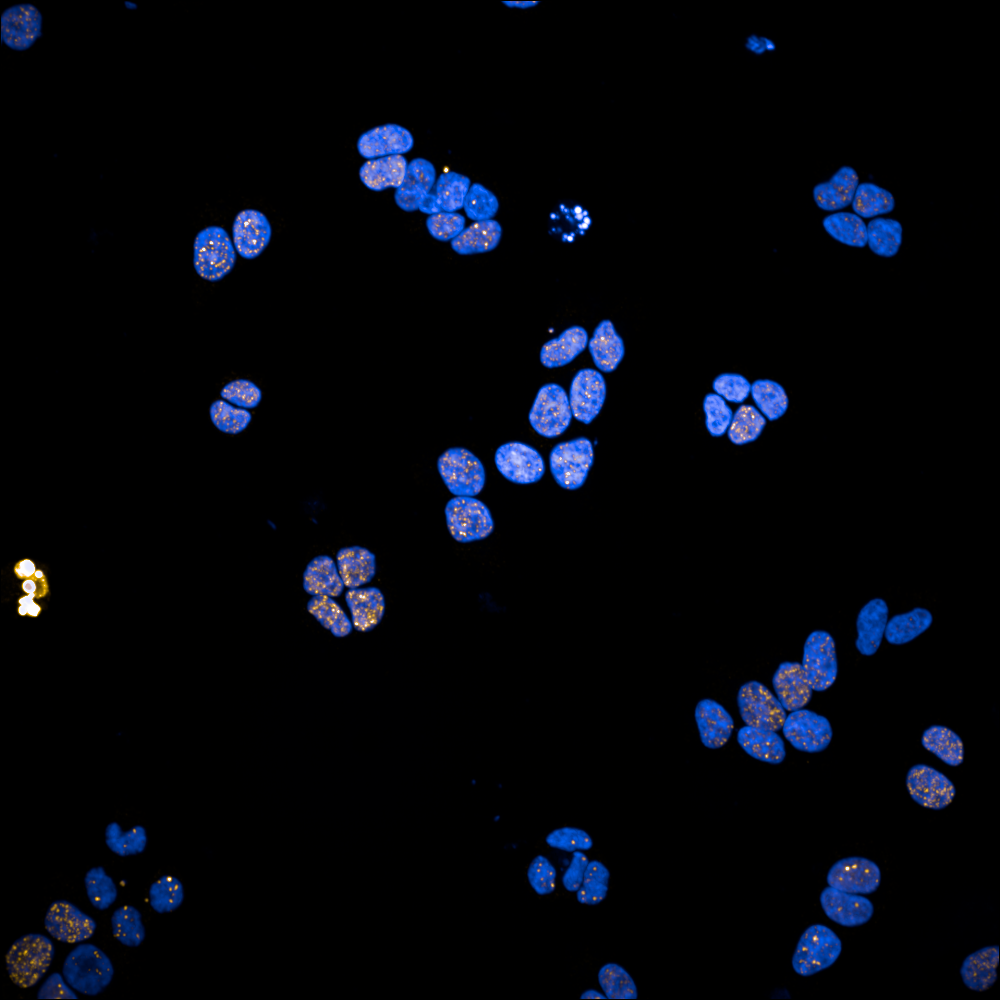

Supplement: Supplementary file 7 — Source data Fig. 3 [file 44319_2025_385_MOESM7_ESM.zip › Figure 3/3D/sgDNMT1#2-DAC.png]

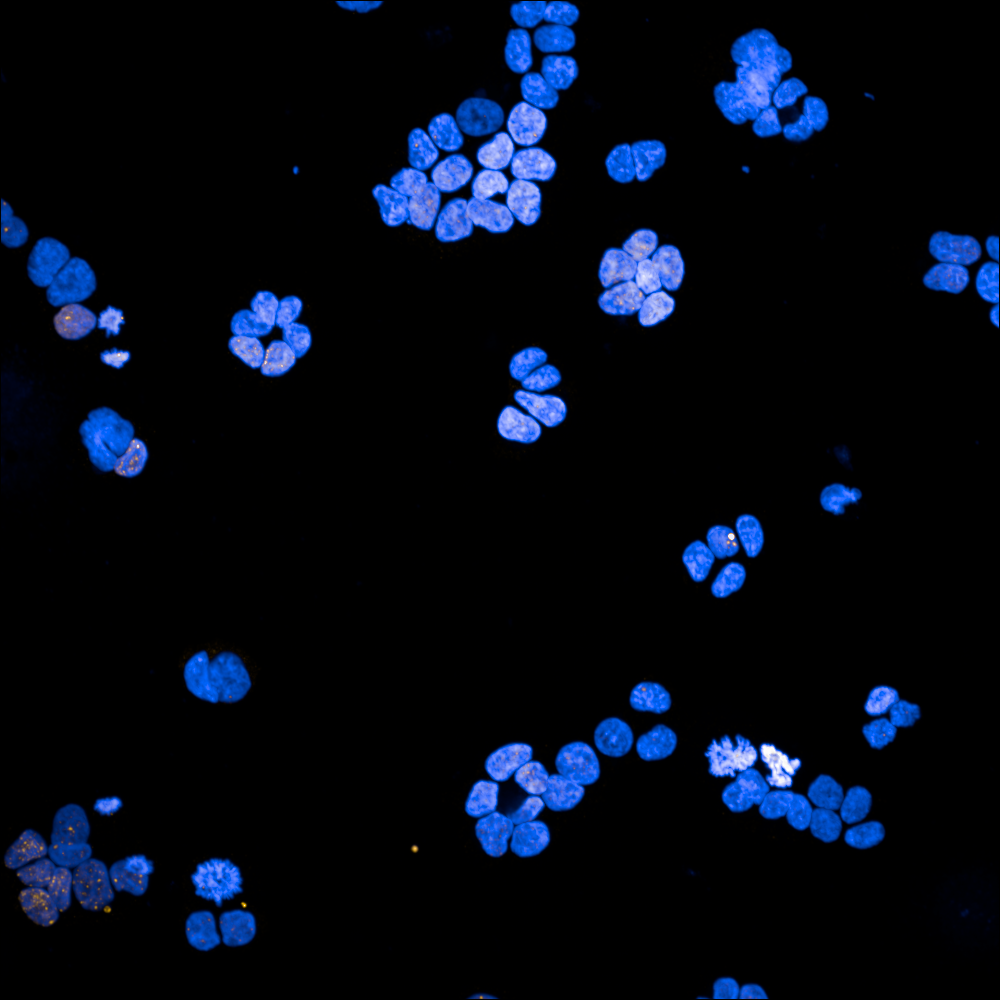

Supplement: Supplementary file 7 — Source data Fig. 3 [file 44319_2025_385_MOESM7_ESM.zip › Figure 3/3D/sgDNMT1#2-DMSO.png]

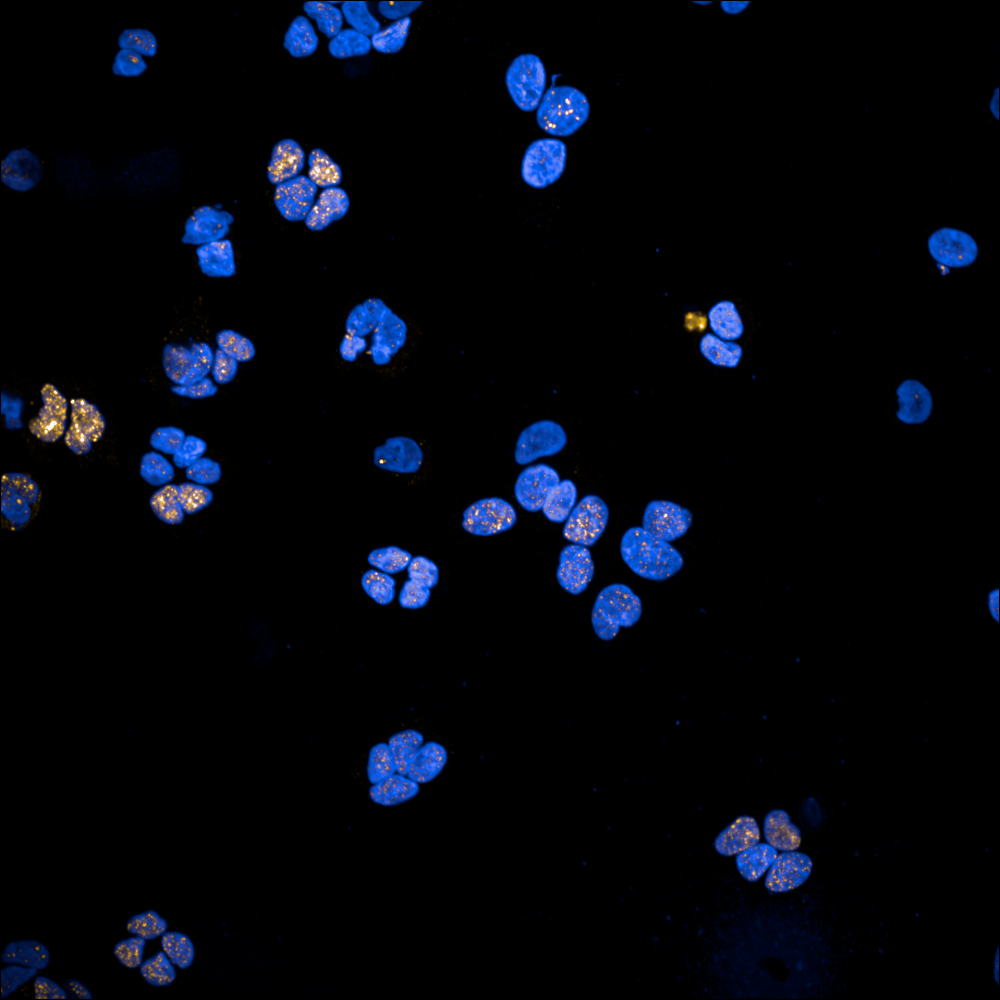

Supplement: Supplementary file 7 — Source data Fig. 3 [file 44319_2025_385_MOESM7_ESM.zip › Figure 3/3D/sgUHRF1#1-DAC.png]

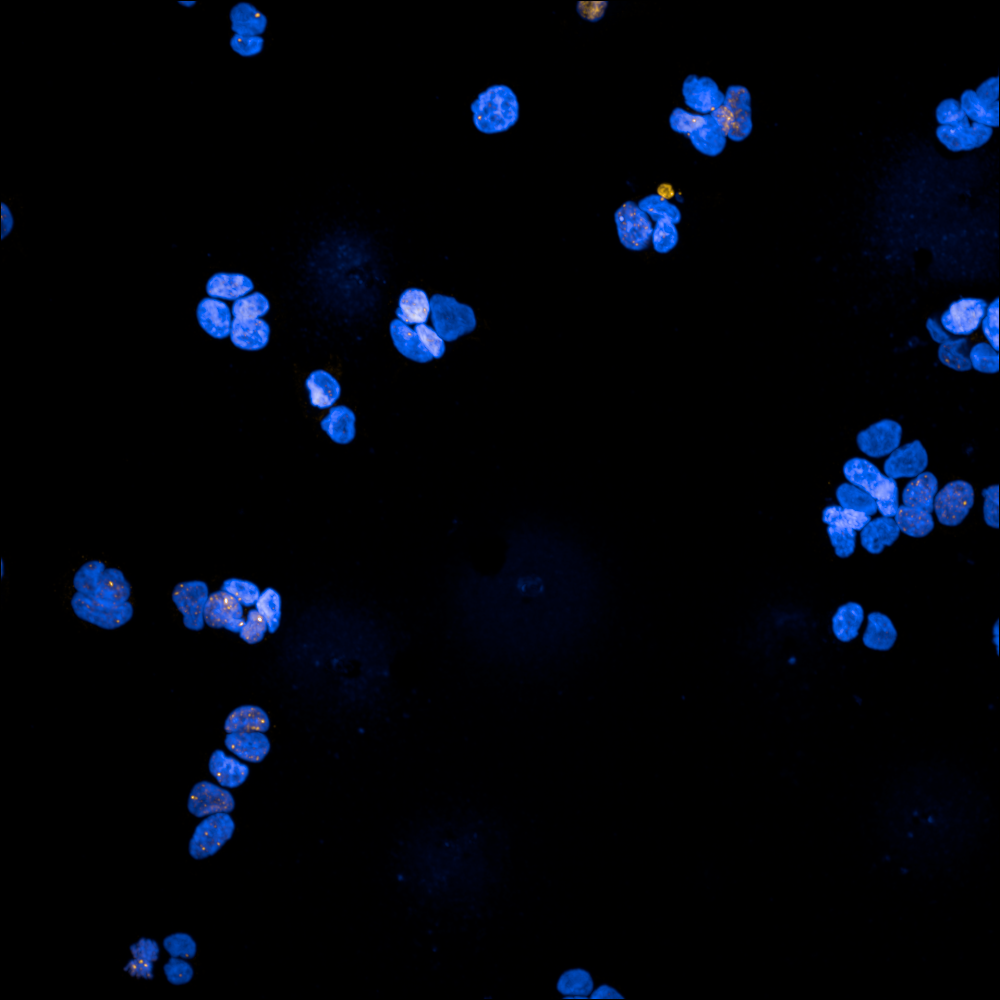

Supplement: Supplementary file 7 — Source data Fig. 3 [file 44319_2025_385_MOESM7_ESM.zip › Figure 3/3D/sgUHRF1#1-DMSO.png]

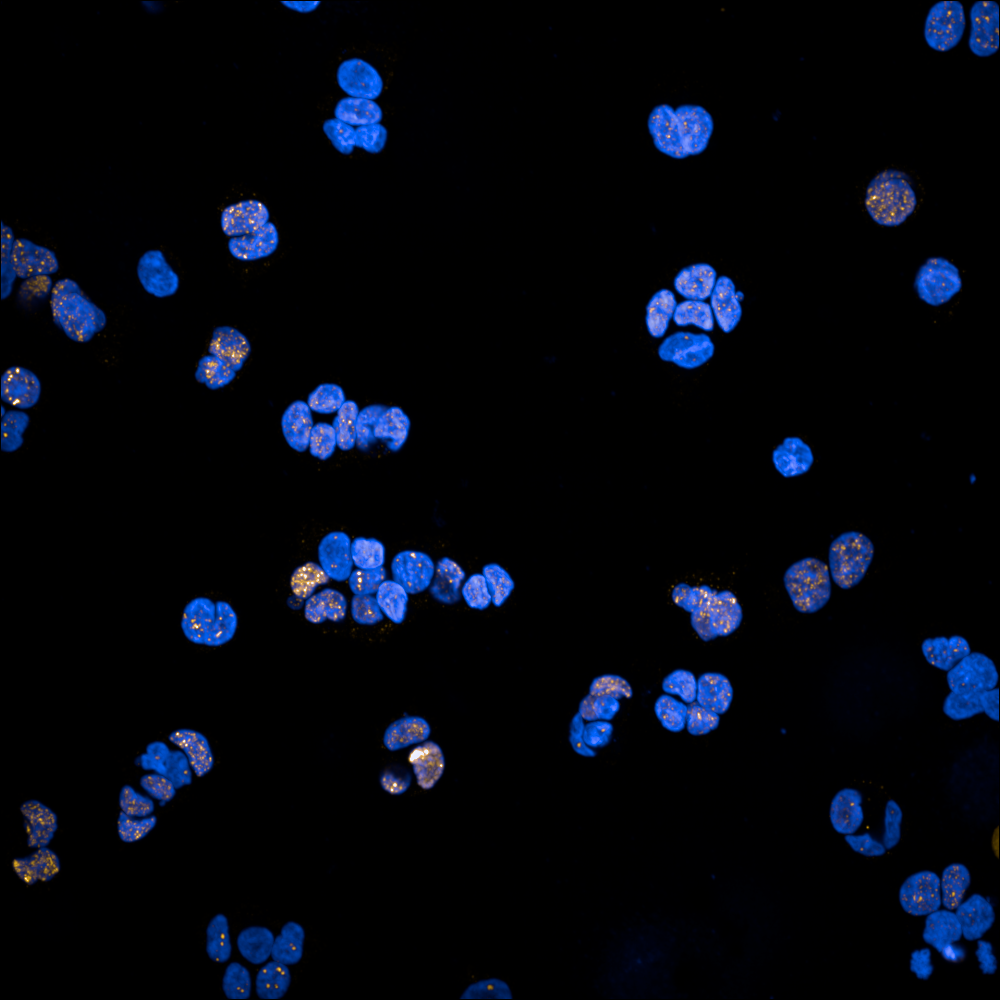

Supplement: Supplementary file 7 — Source data Fig. 3 [file 44319_2025_385_MOESM7_ESM.zip › Figure 3/3D/sgUHRF1#2-DAC.png]

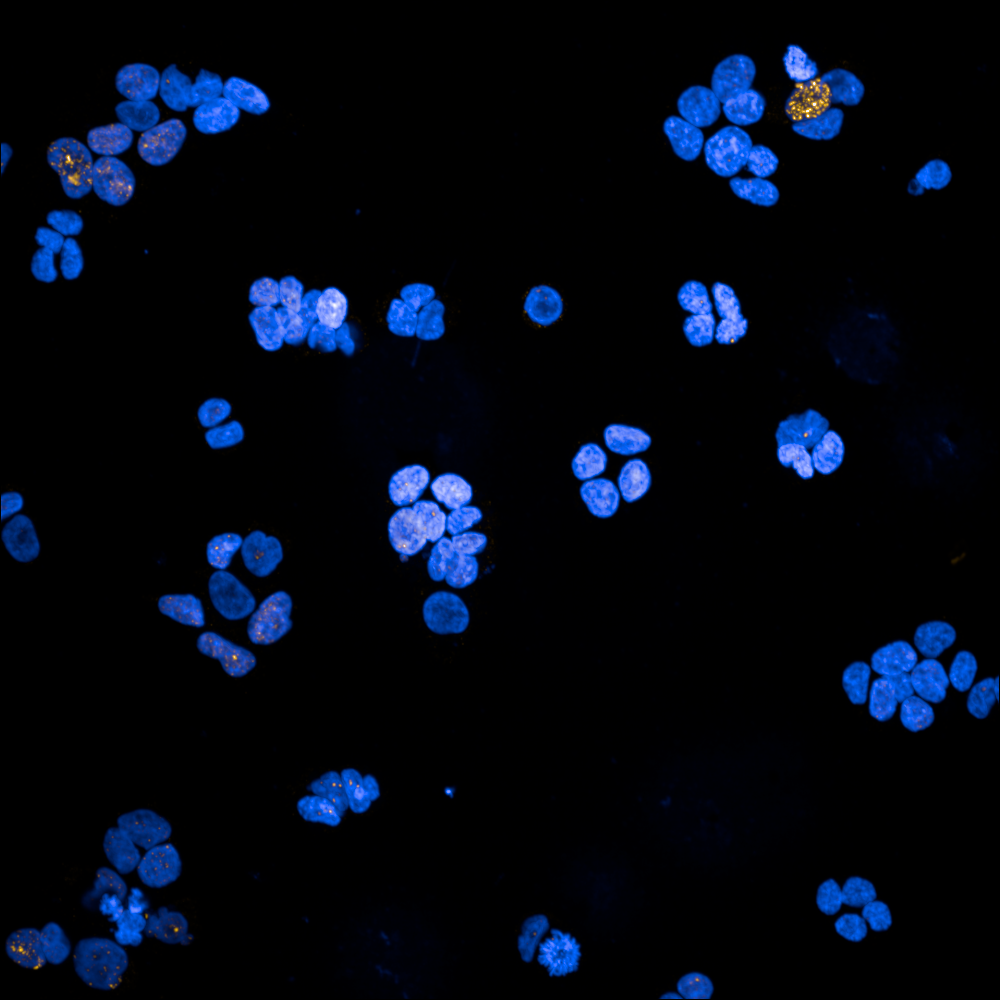

Supplement: Supplementary file 7 — Source data Fig. 3 [file 44319_2025_385_MOESM7_ESM.zip › Figure 3/3D/sgUHRF1#2-DMSO.png]

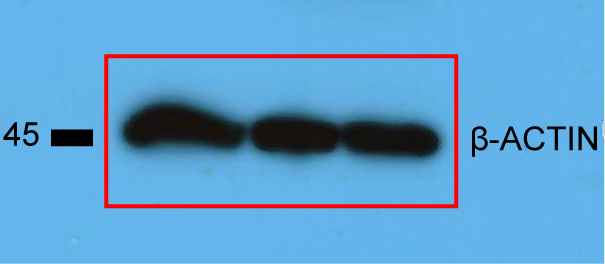

Supplement: Supplementary file 7 — Source data Fig. 3 [file 44319_2025_385_MOESM7_ESM.zip › Figure 3/3F/Western-ACTB.tif]

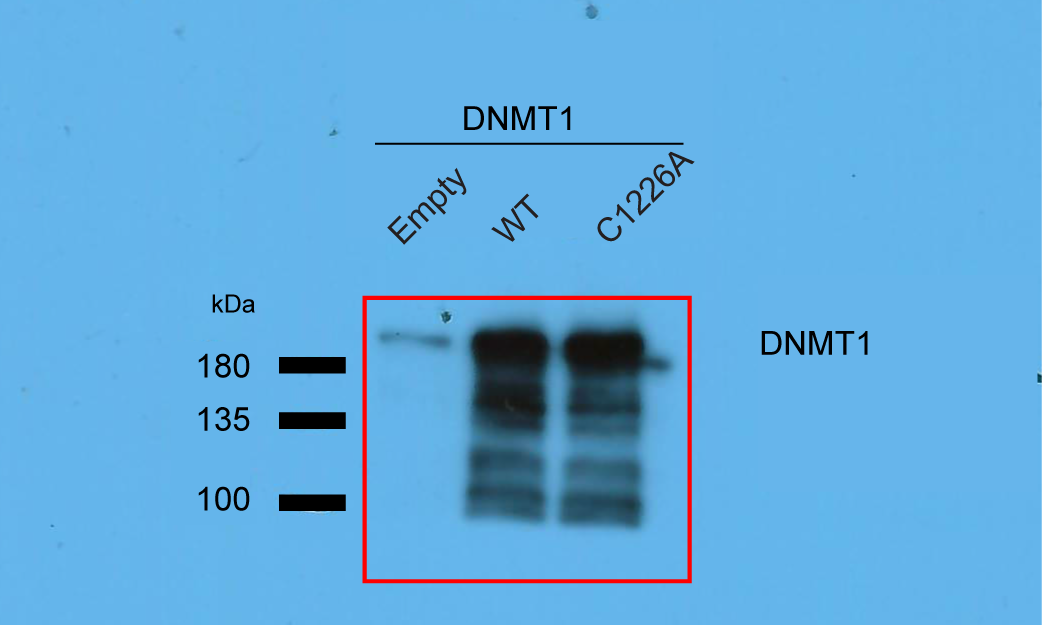

Supplement: Supplementary file 7 — Source data Fig. 3 [file 44319_2025_385_MOESM7_ESM.zip › Figure 3/3F/Western-DNMT1.tif]

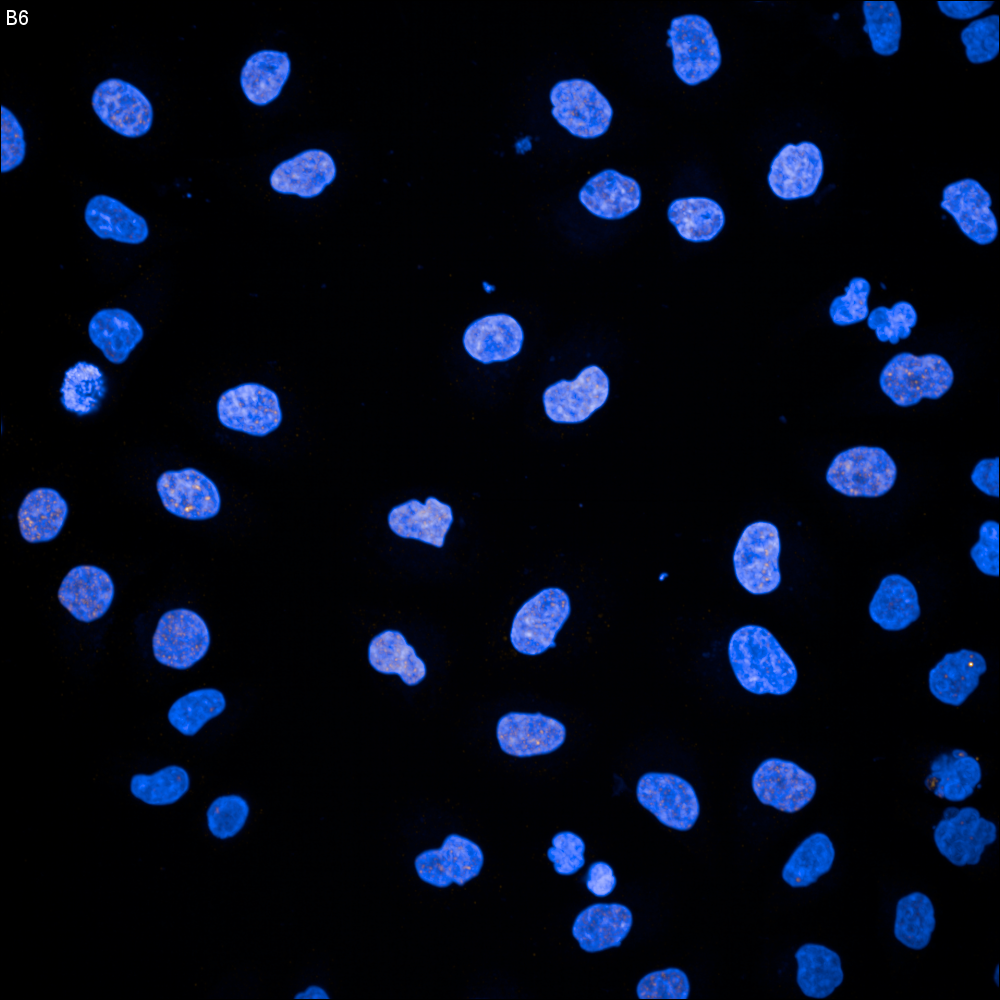

Supplement: Supplementary file 7 — Source data Fig. 3 [file 44319_2025_385_MOESM7_ESM.zip › Figure 3/3G/DNMT1-C1226A-DAC.png]

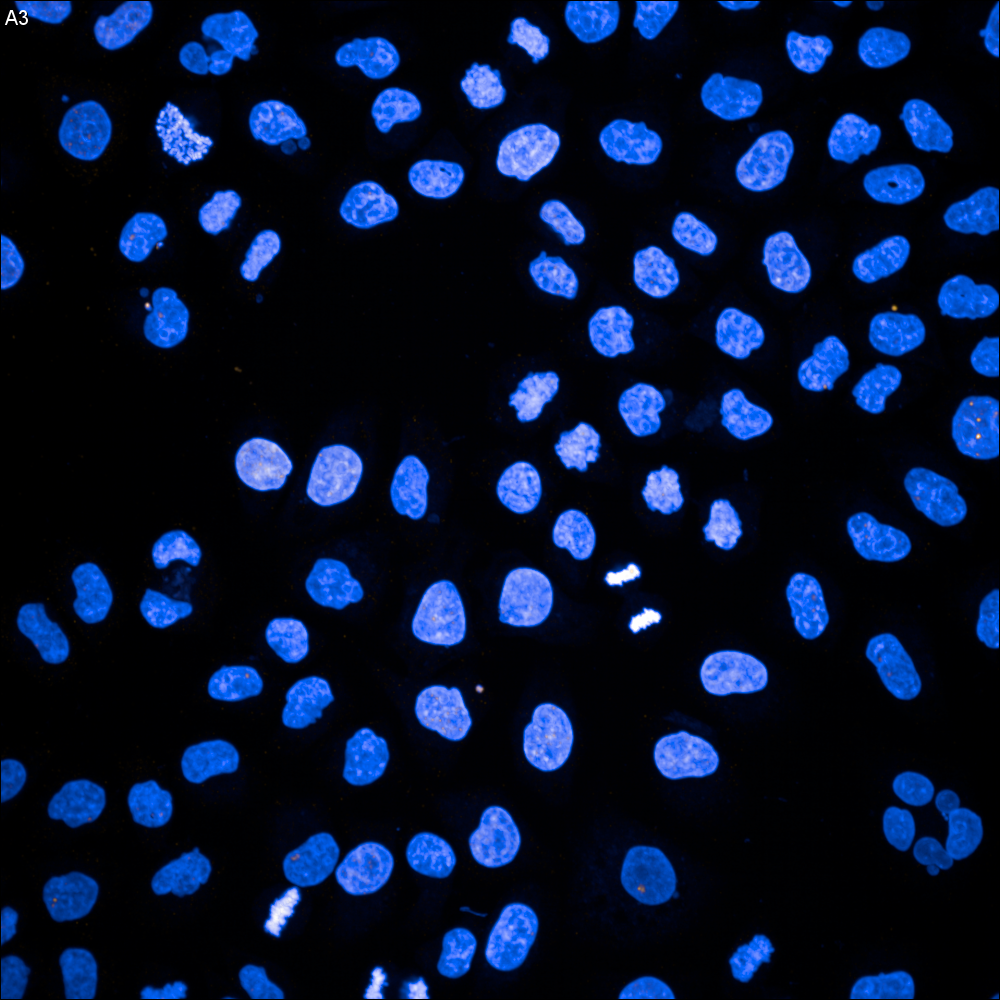

Supplement: Supplementary file 7 — Source data Fig. 3 [file 44319_2025_385_MOESM7_ESM.zip › Figure 3/3G/DNMT1-C1226A-DMSO.png]

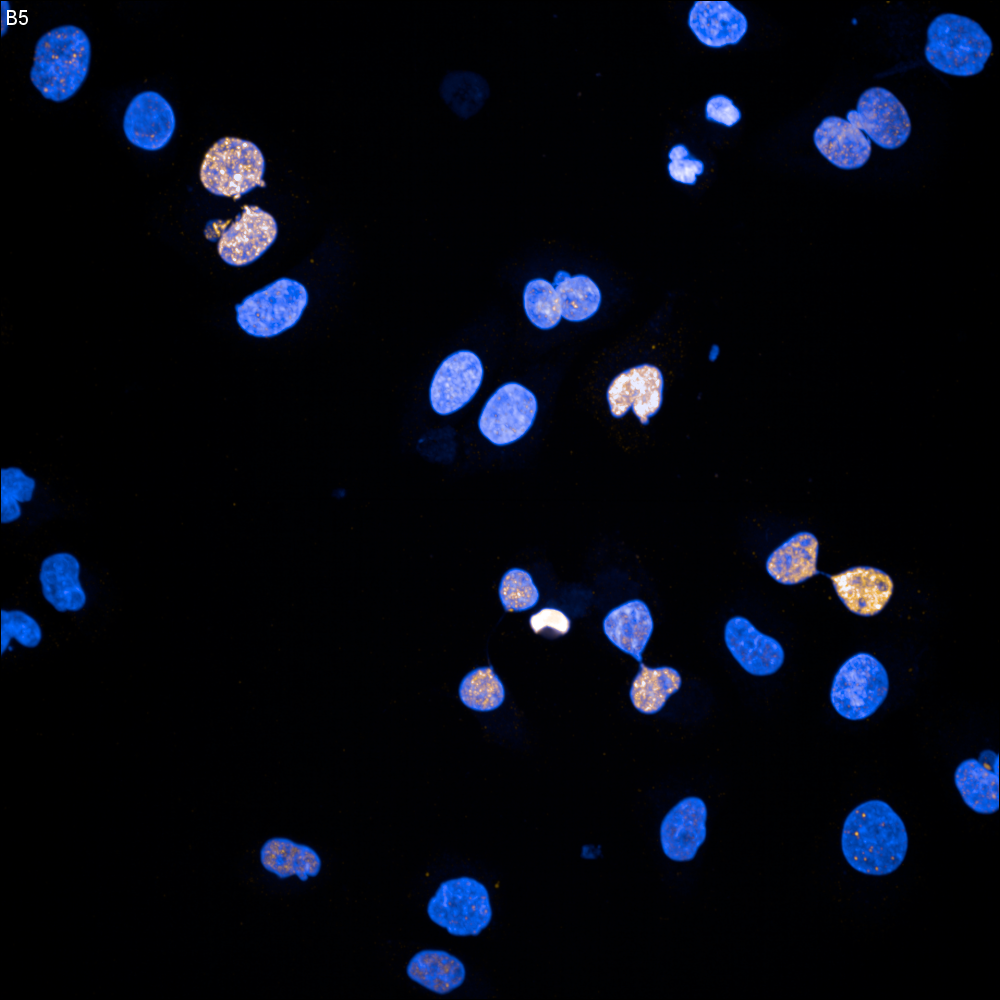

Supplement: Supplementary file 7 — Source data Fig. 3 [file 44319_2025_385_MOESM7_ESM.zip › Figure 3/3G/DNMT1-WT-DAC.png]

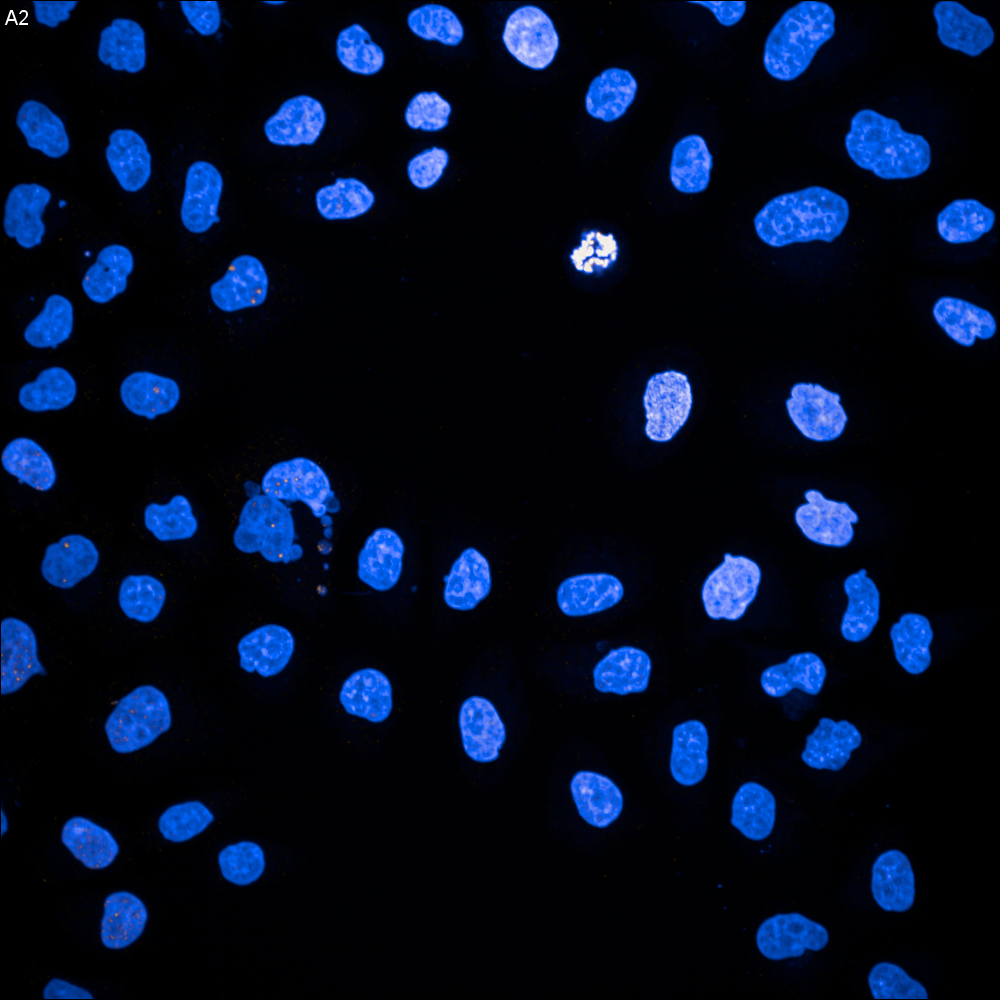

Supplement: Supplementary file 7 — Source data Fig. 3 [file 44319_2025_385_MOESM7_ESM.zip › Figure 3/3G/DNMT1-WT-DMSO.png]

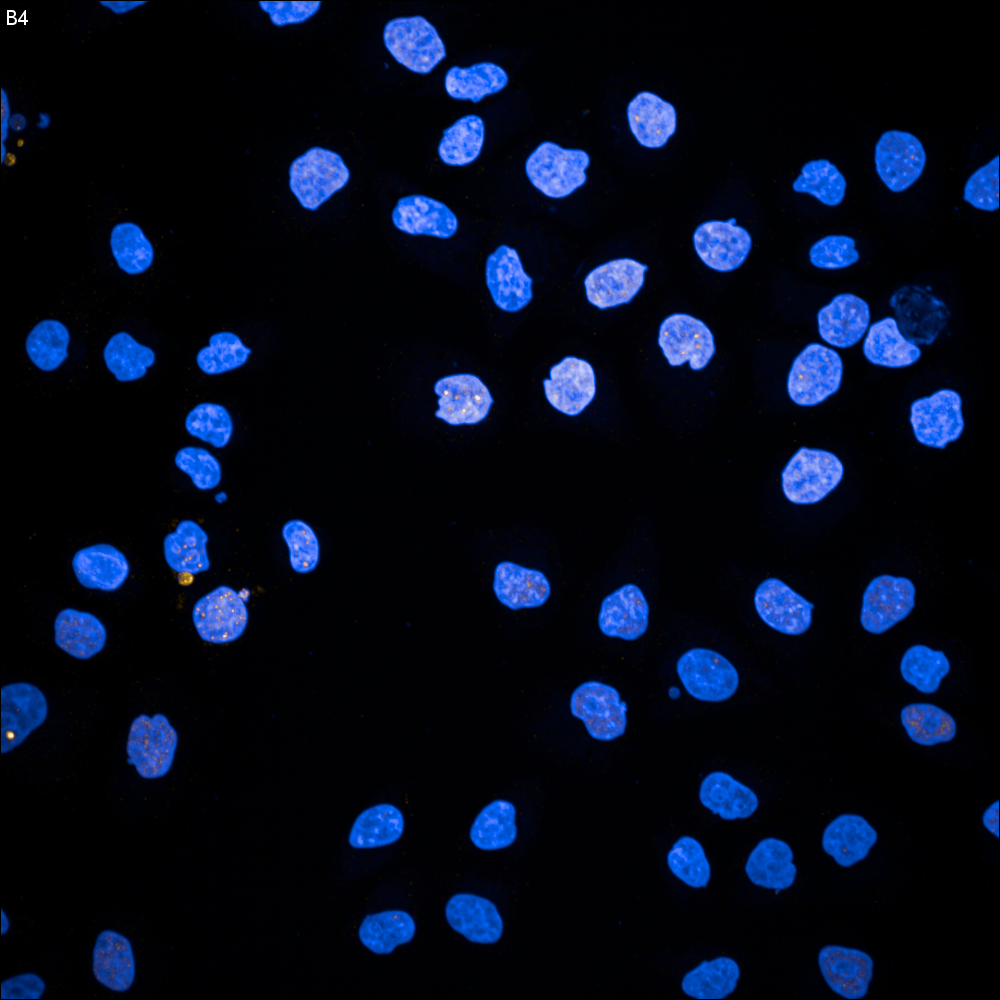

Supplement: Supplementary file 7 — Source data Fig. 3 [file 44319_2025_385_MOESM7_ESM.zip › Figure 3/3G/Empty-DAC.png]

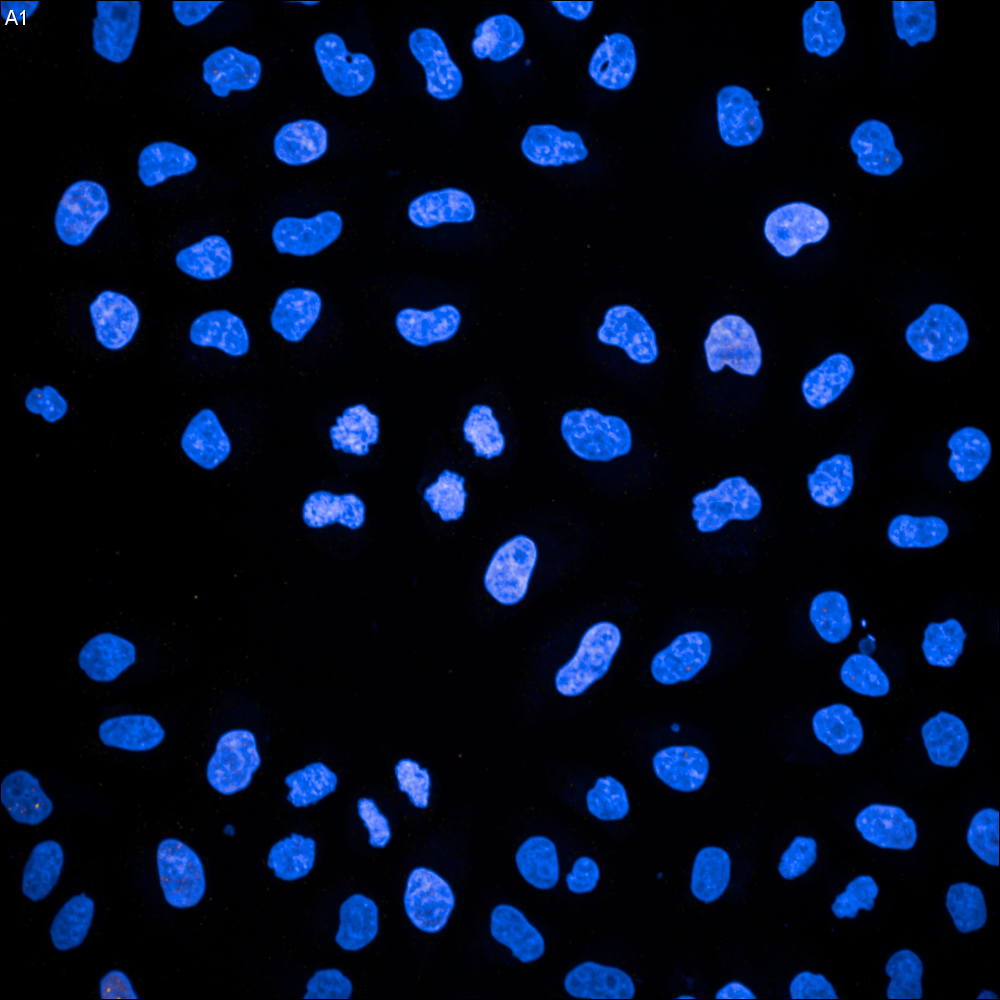

Supplement: Supplementary file 7 — Source data Fig. 3 [file 44319_2025_385_MOESM7_ESM.zip › Figure 3/3G/Empty-DMSO.png]

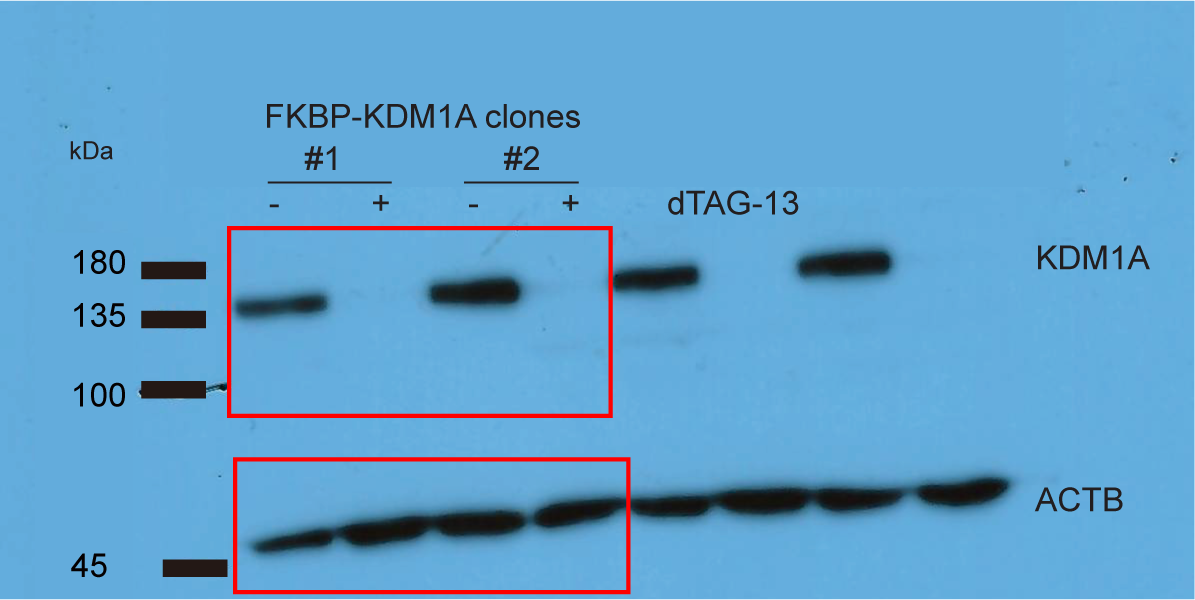

Supplement: Supplementary file 9 — Source data Fig. 5 [file 44319_2025_385_MOESM9_ESM.zip › Figure 5/5D/Western-KDM1A-ACTB.tif]

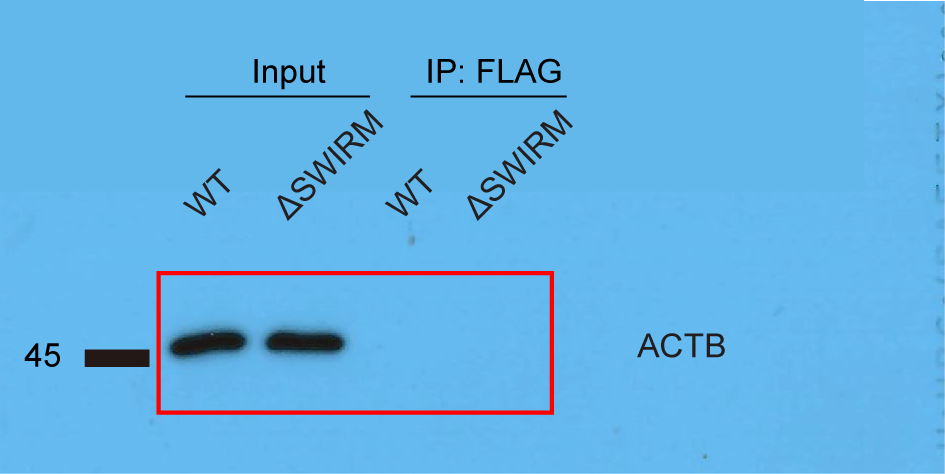

Supplement: Supplementary file 9 — Source data Fig. 5 [file 44319_2025_385_MOESM9_ESM.zip › Figure 5/5J/Western-ACTB.tif]

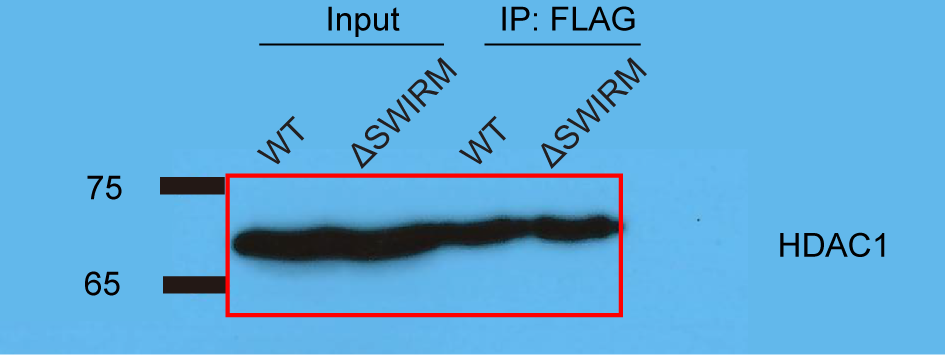

Supplement: Supplementary file 9 — Source data Fig. 5 [file 44319_2025_385_MOESM9_ESM.zip › Figure 5/5J/Western-HDAC1.tif]

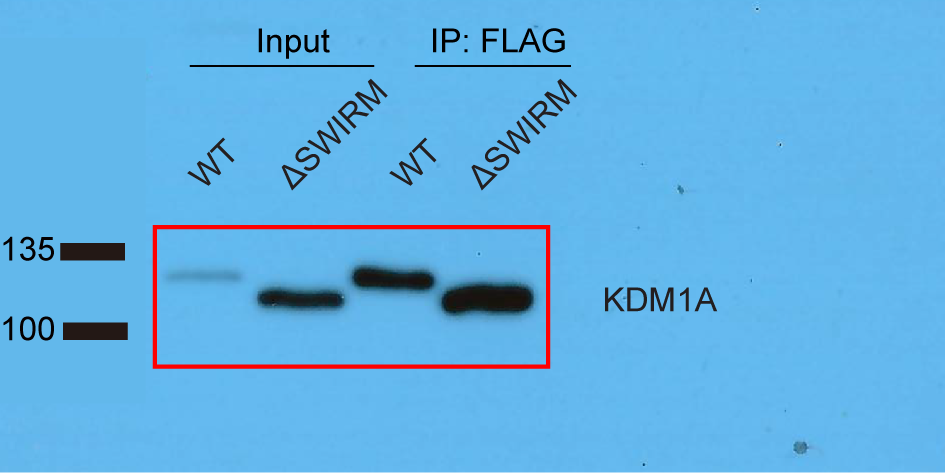

Supplement: Supplementary file 9 — Source data Fig. 5 [file 44319_2025_385_MOESM9_ESM.zip › Figure 5/5J/Western-KDM1A.tif]

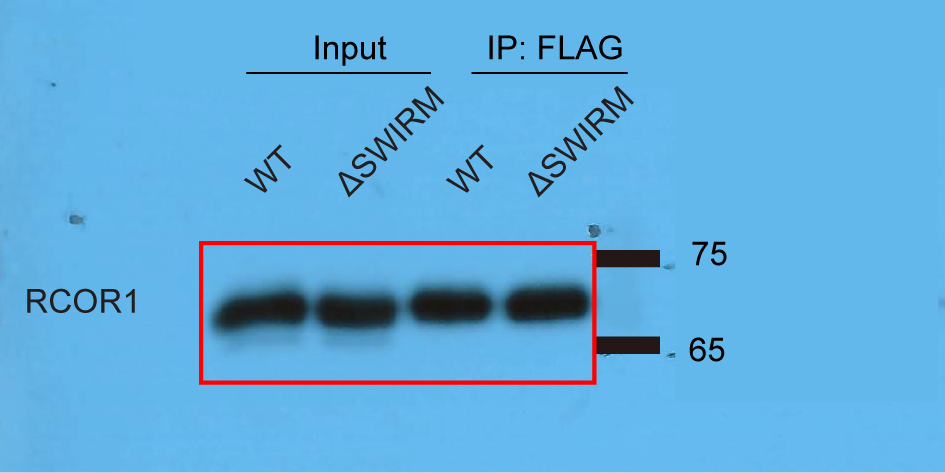

Supplement: Supplementary file 9 — Source data Fig. 5 [file 44319_2025_385_MOESM9_ESM.zip › Figure 5/5J/Western-RCOR1.tif]

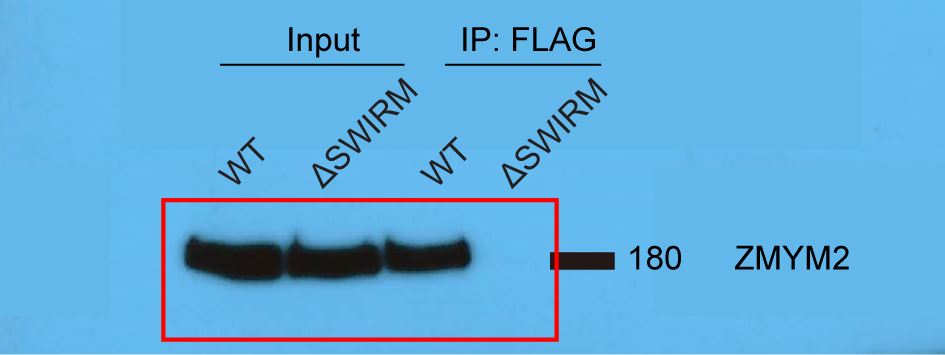

Supplement: Supplementary file 9 — Source data Fig. 5 [file 44319_2025_385_MOESM9_ESM.zip › Figure 5/5J/Western-ZMYM2.tif]

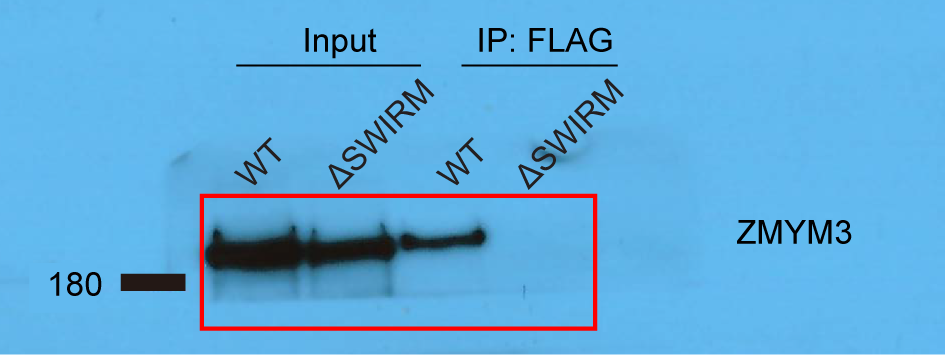

Supplement: Supplementary file 9 — Source data Fig. 5 [file 44319_2025_385_MOESM9_ESM.zip › Figure 5/5J/Western-ZMYM3.tif]

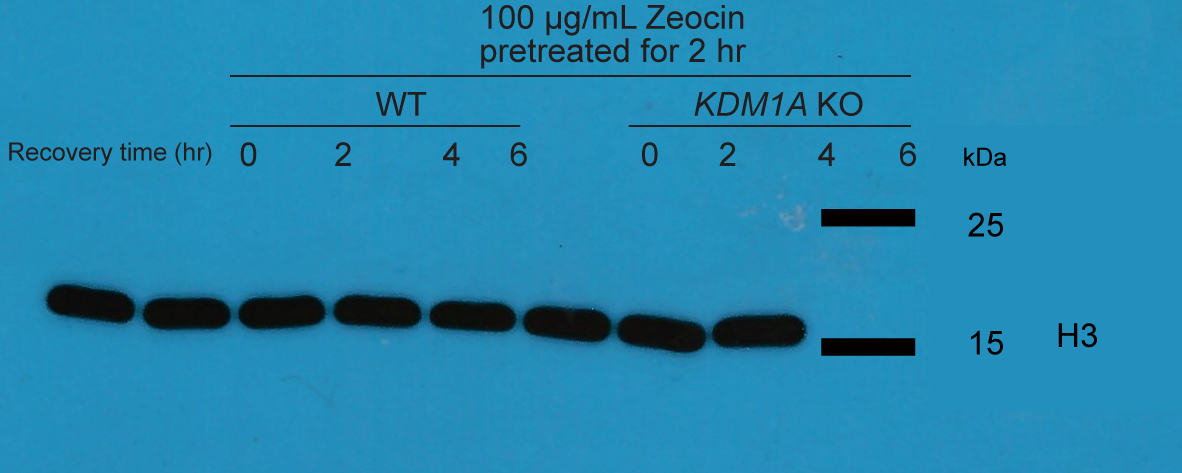

Supplement: Supplementary file 10 — Source data Fig. 6 [file 44319_2025_385_MOESM10_ESM.zip › Figure 6/6B/Western-H3.tif]

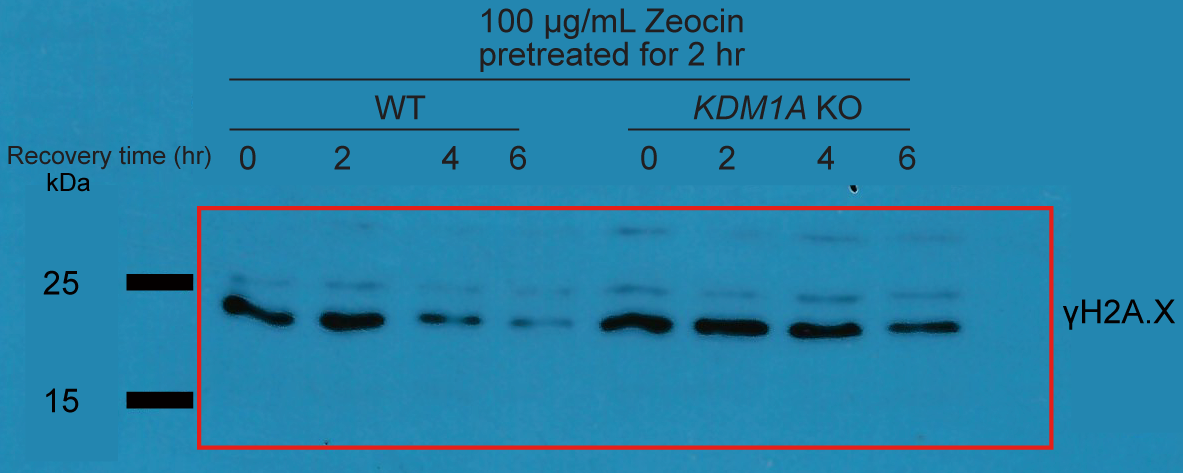

Supplement: Supplementary file 10 — Source data Fig. 6 [file 44319_2025_385_MOESM10_ESM.zip › Figure 6/6B/Western-γH2AX.tif]

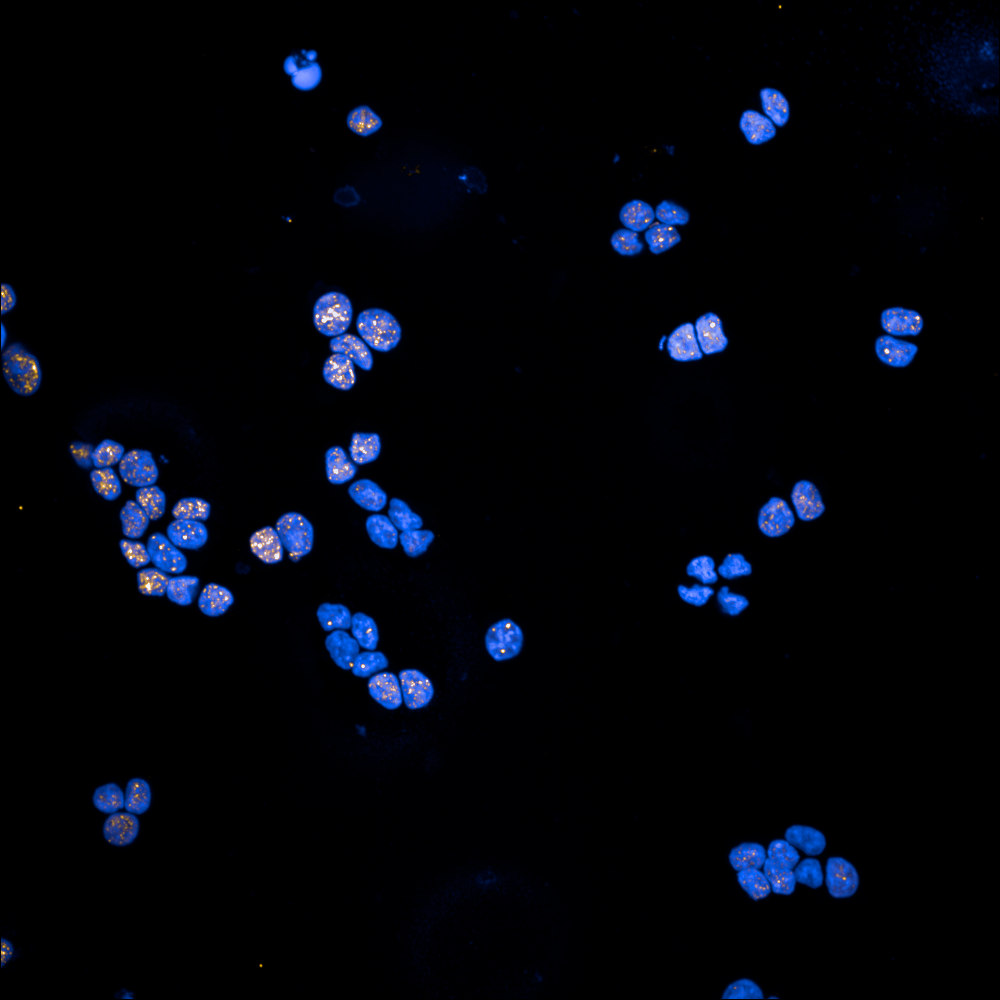

Supplement: Supplementary file 10 — Source data Fig. 6 [file 44319_2025_385_MOESM10_ESM.zip › Figure 6/6C/KDM1A_KO-DAC.png]

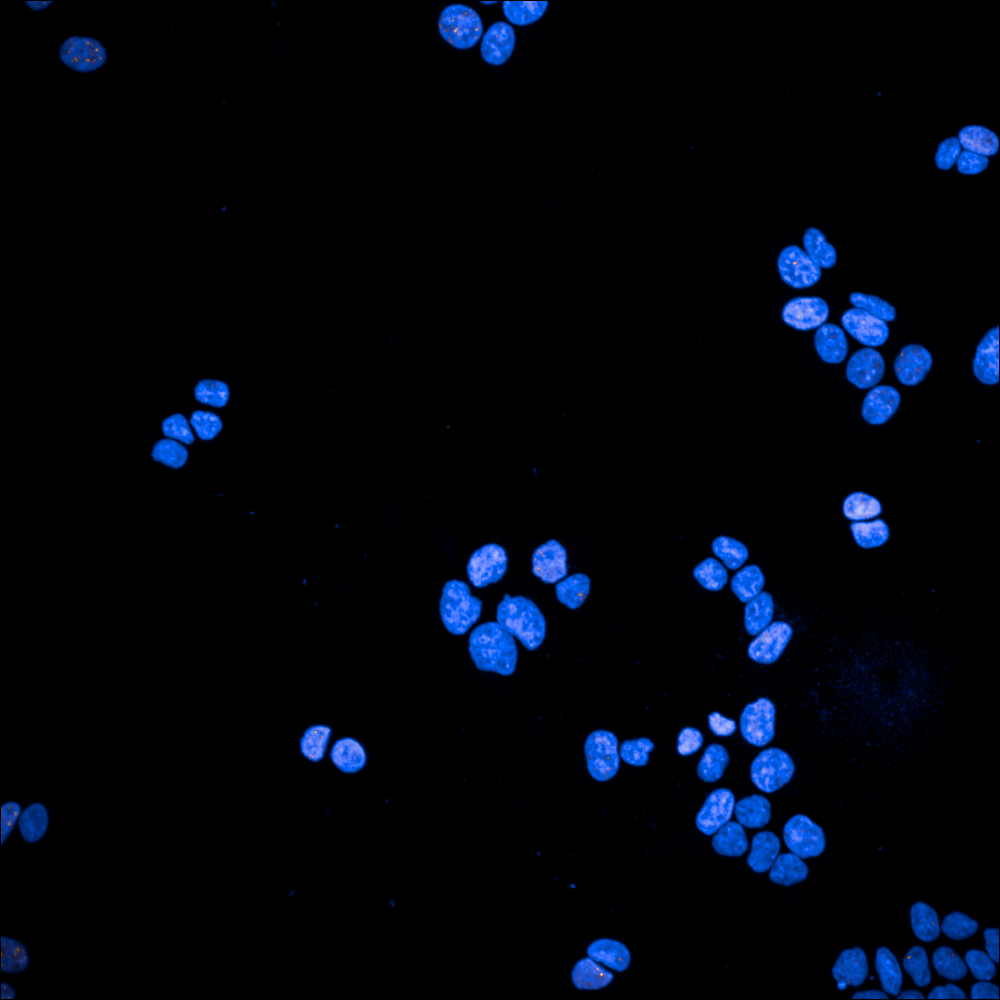

Supplement: Supplementary file 10 — Source data Fig. 6 [file 44319_2025_385_MOESM10_ESM.zip › Figure 6/6C/KDM1A_KO-DMSO.png]

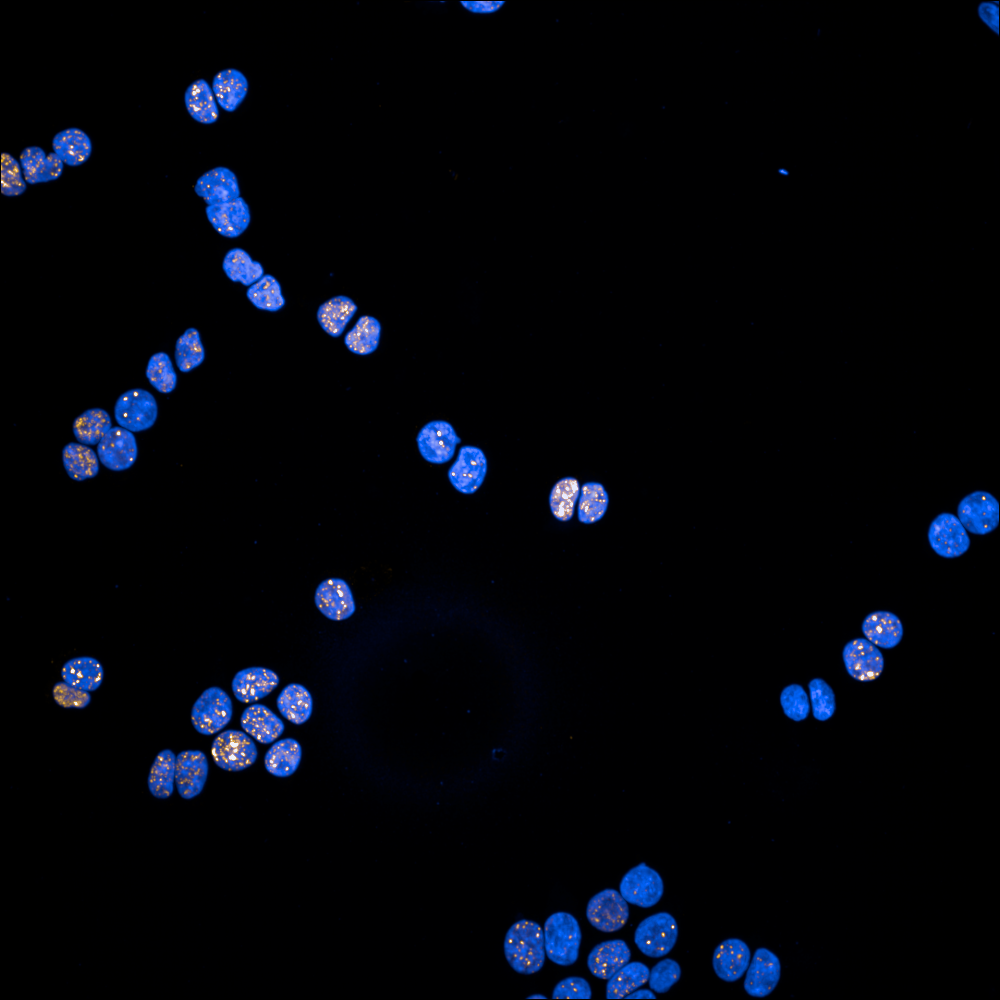

Supplement: Supplementary file 10 — Source data Fig. 6 [file 44319_2025_385_MOESM10_ESM.zip › Figure 6/6C/KO+Empty-DAC.png]

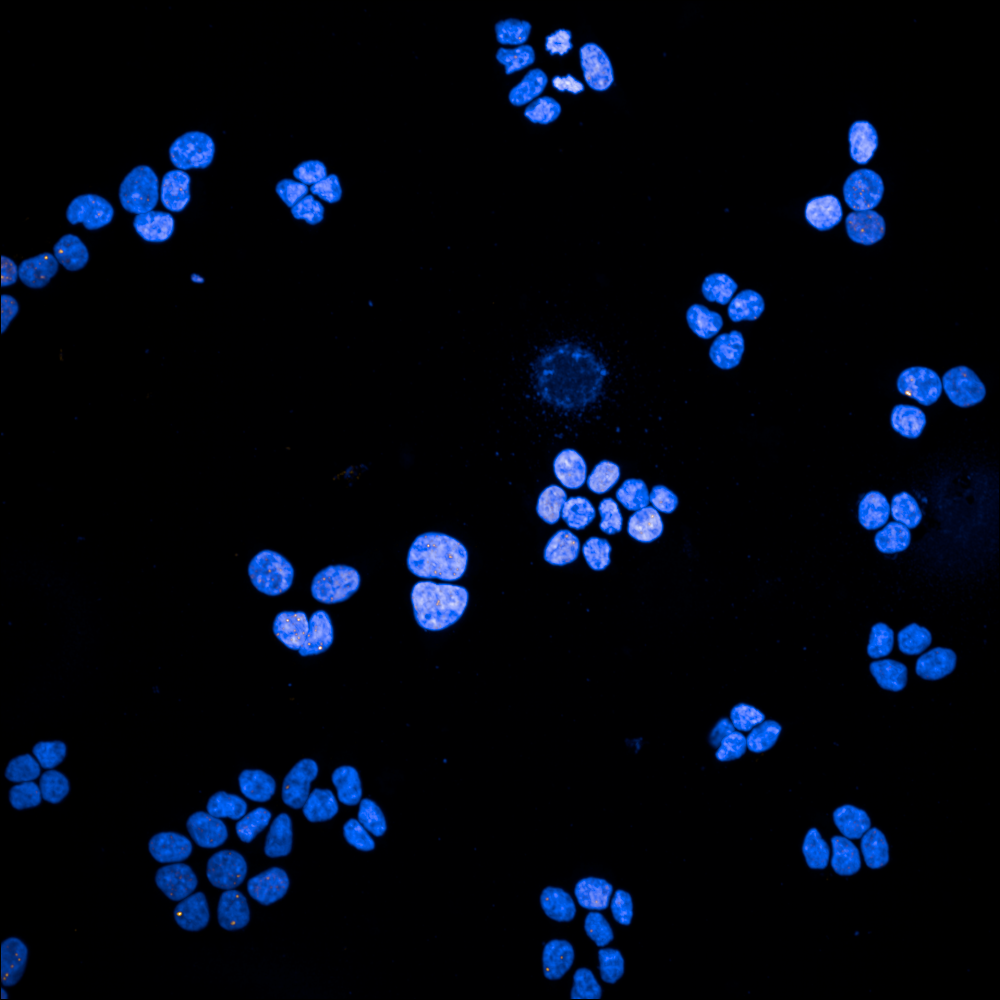

Supplement: Supplementary file 10 — Source data Fig. 6 [file 44319_2025_385_MOESM10_ESM.zip › Figure 6/6C/KO+Empty-DMSO.png]

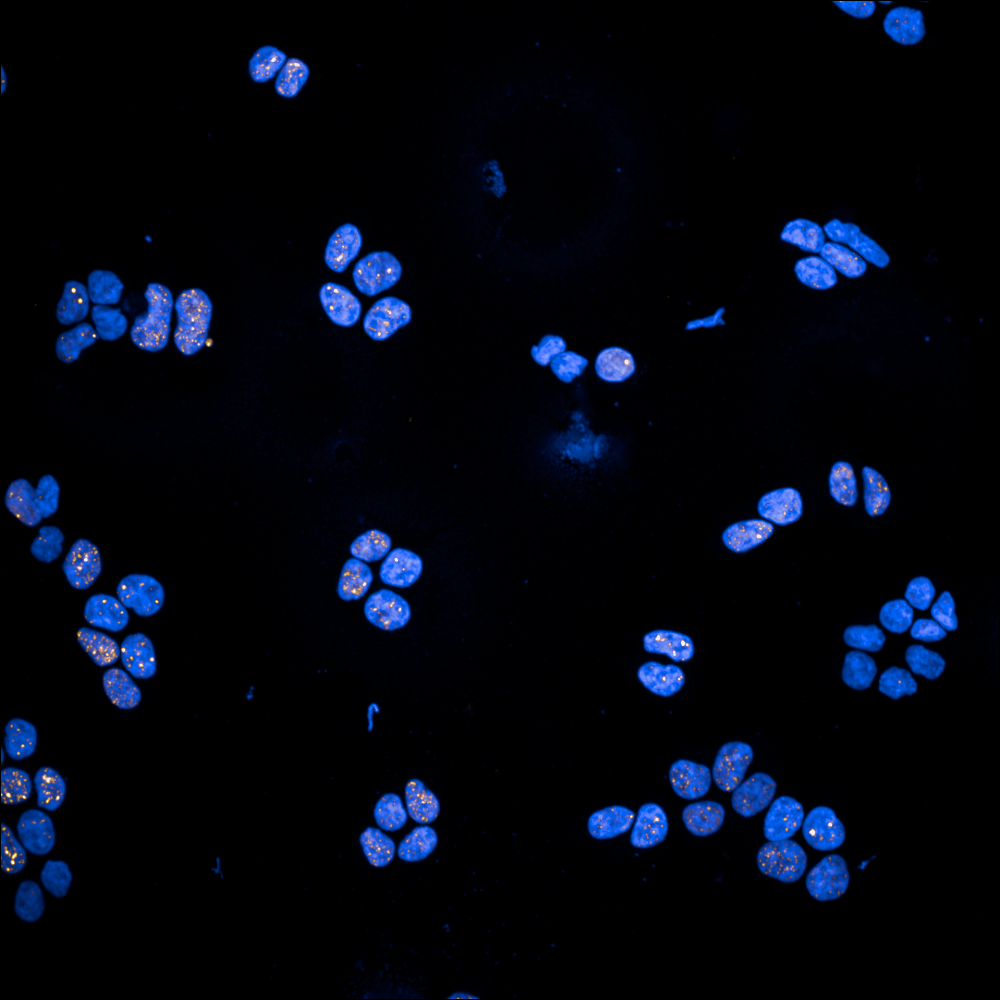

Supplement: Supplementary file 10 — Source data Fig. 6 [file 44319_2025_385_MOESM10_ESM.zip › Figure 6/6C/KO+KDM1A-AE KA-DAC.png]

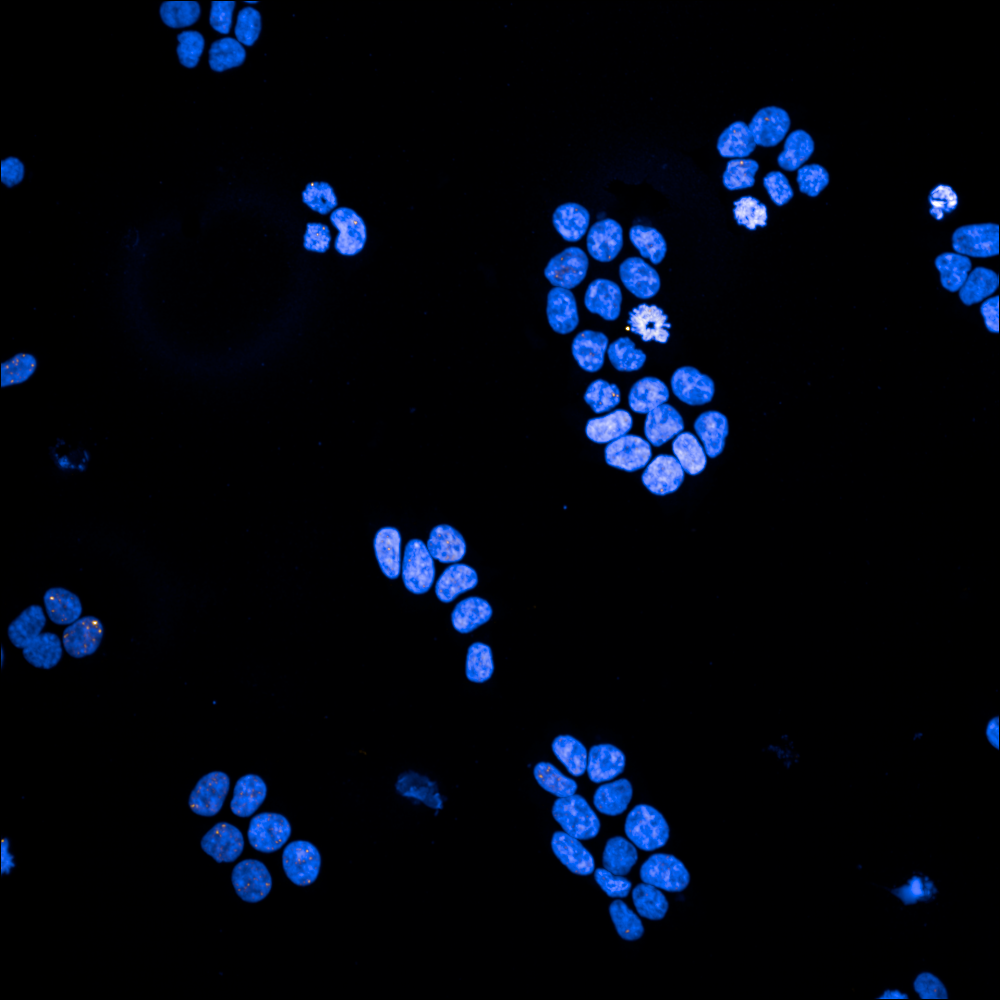

Supplement: Supplementary file 10 — Source data Fig. 6 [file 44319_2025_385_MOESM10_ESM.zip › Figure 6/6C/KO+KDM1A-AE KA-DMSO.png]

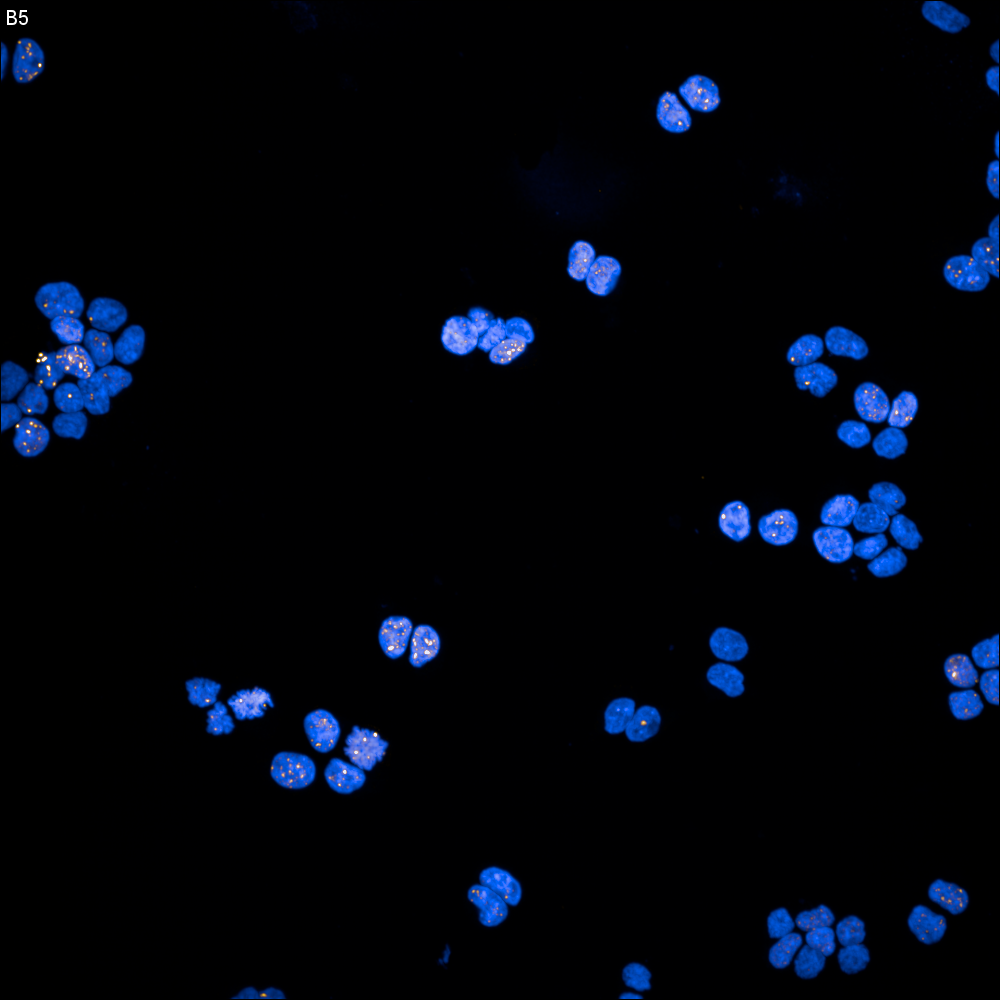

Supplement: Supplementary file 10 — Source data Fig. 6 [file 44319_2025_385_MOESM10_ESM.zip › Figure 6/6C/KO+KDM1A-WT-DAC.png]

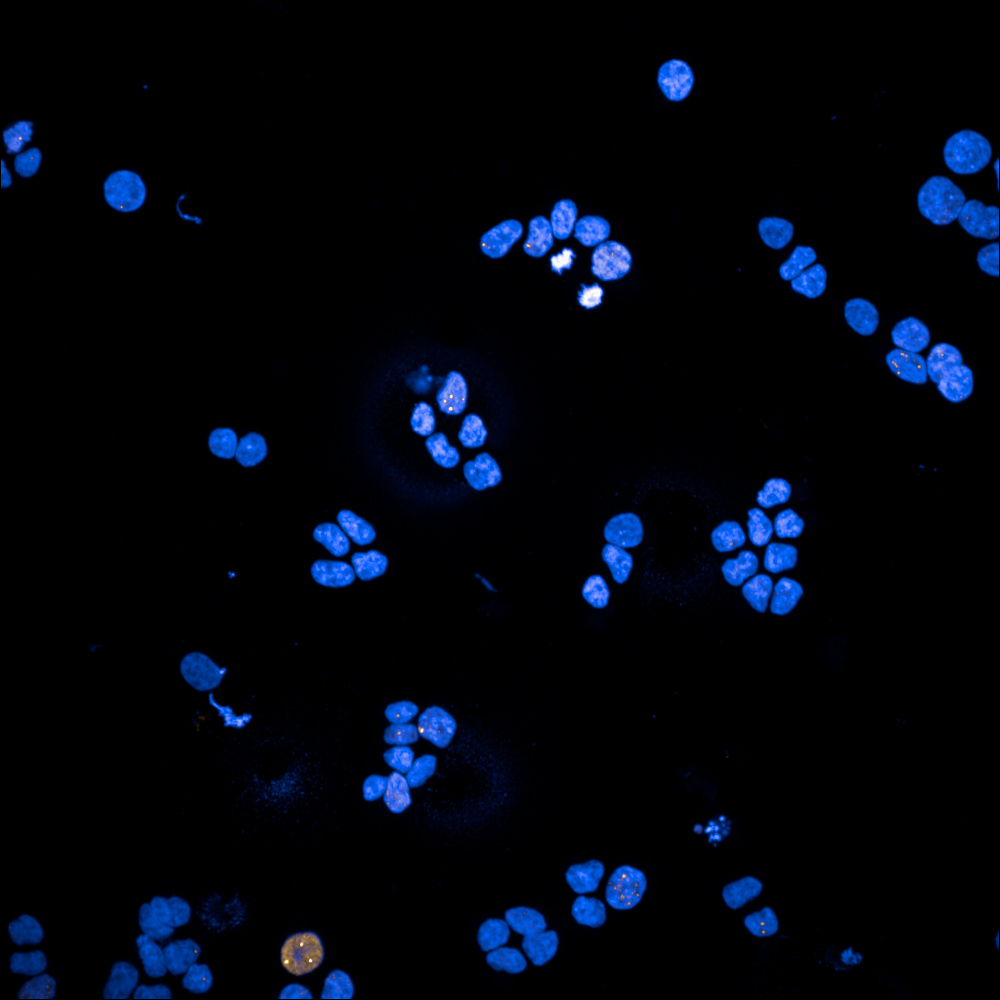

Supplement: Supplementary file 10 — Source data Fig. 6 [file 44319_2025_385_MOESM10_ESM.zip › Figure 6/6C/KO+KDM1A-WT-DMSO.png]

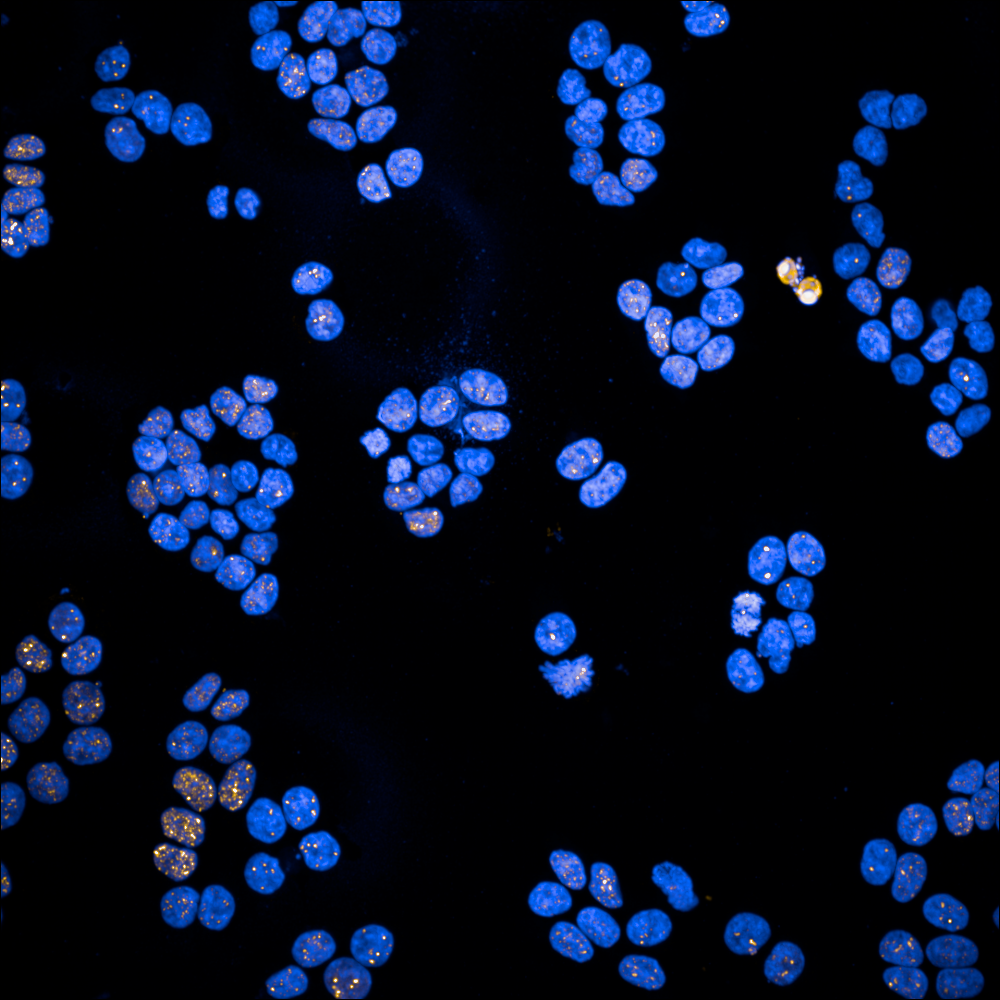

Supplement: Supplementary file 10 — Source data Fig. 6 [file 44319_2025_385_MOESM10_ESM.zip › Figure 6/6C/KO+ΔNFR-DAC.png]

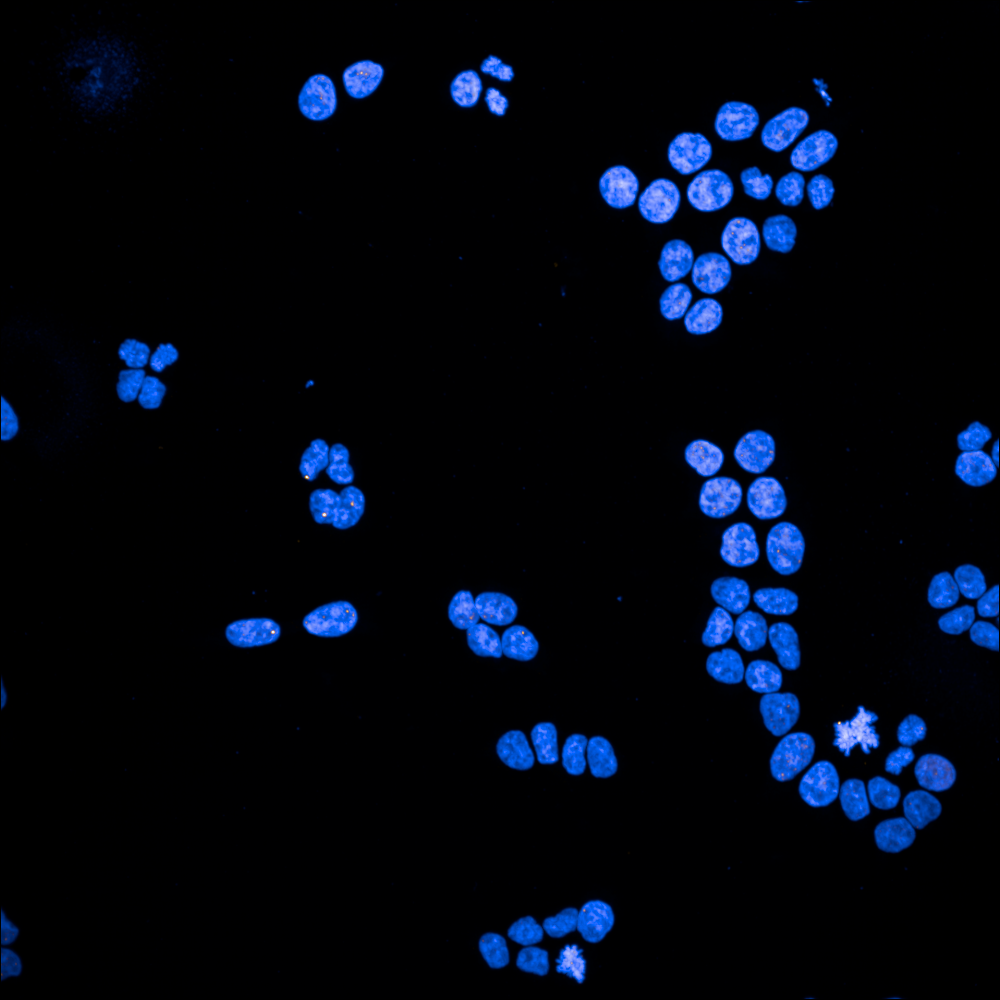

Supplement: Supplementary file 10 — Source data Fig. 6 [file 44319_2025_385_MOESM10_ESM.zip › Figure 6/6C/KO+ΔNFR-DMSO.png]

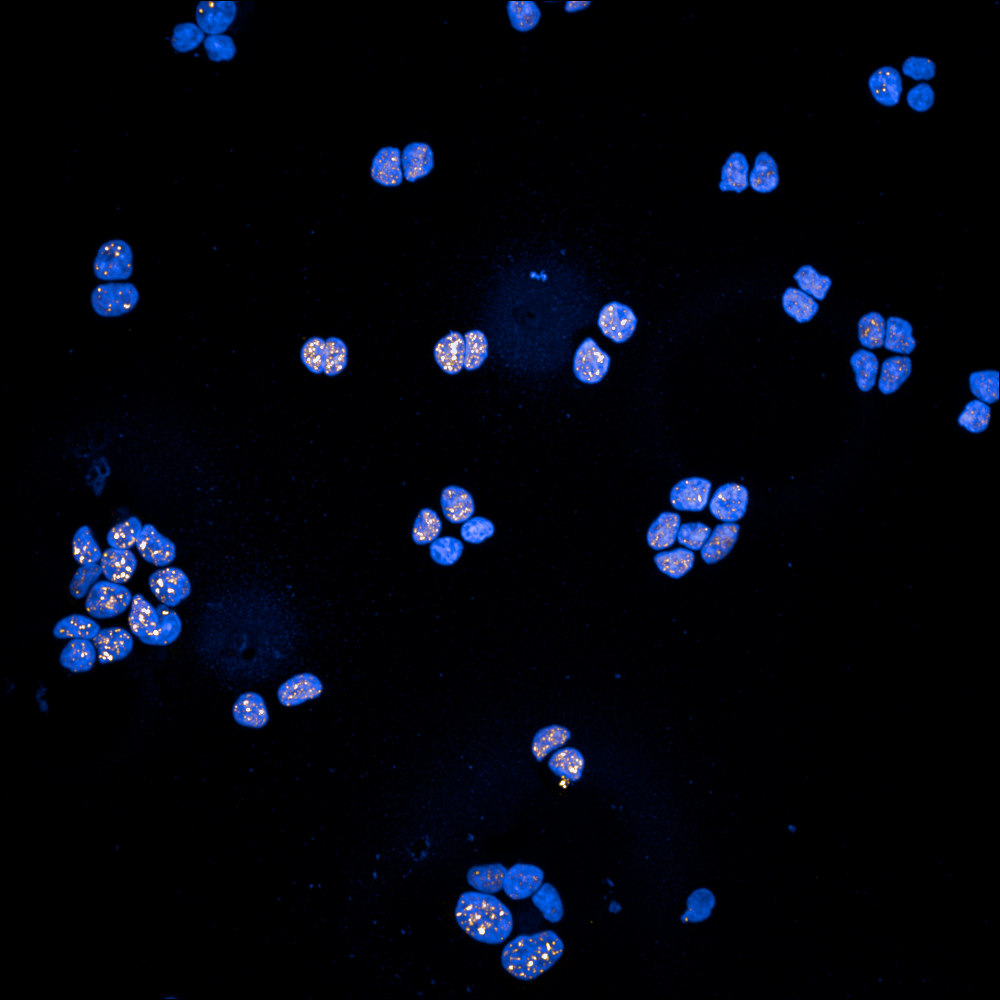

Supplement: Supplementary file 10 — Source data Fig. 6 [file 44319_2025_385_MOESM10_ESM.zip › Figure 6/6C/KO+ΔSWIRM-DAC.png]

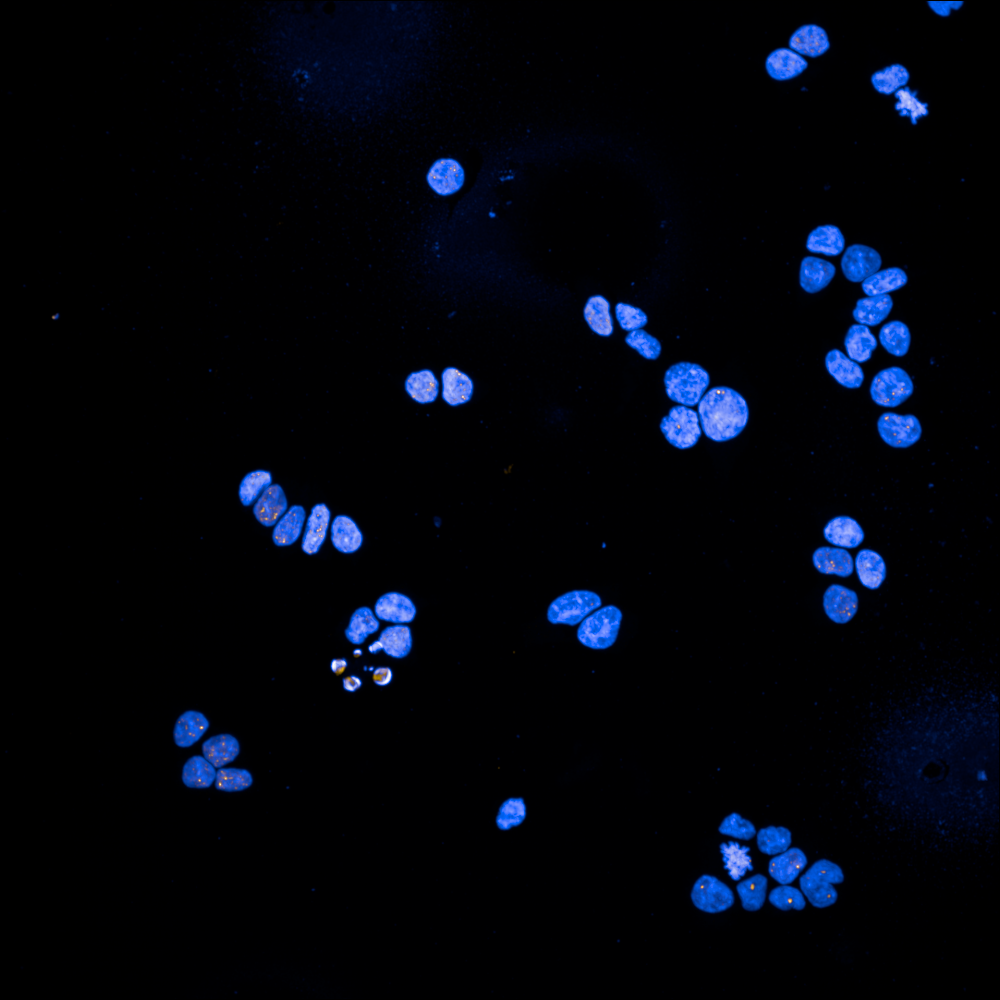

Supplement: Supplementary file 10 — Source data Fig. 6 [file 44319_2025_385_MOESM10_ESM.zip › Figure 6/6C/KO+ΔSWIRM-DMSO.png]

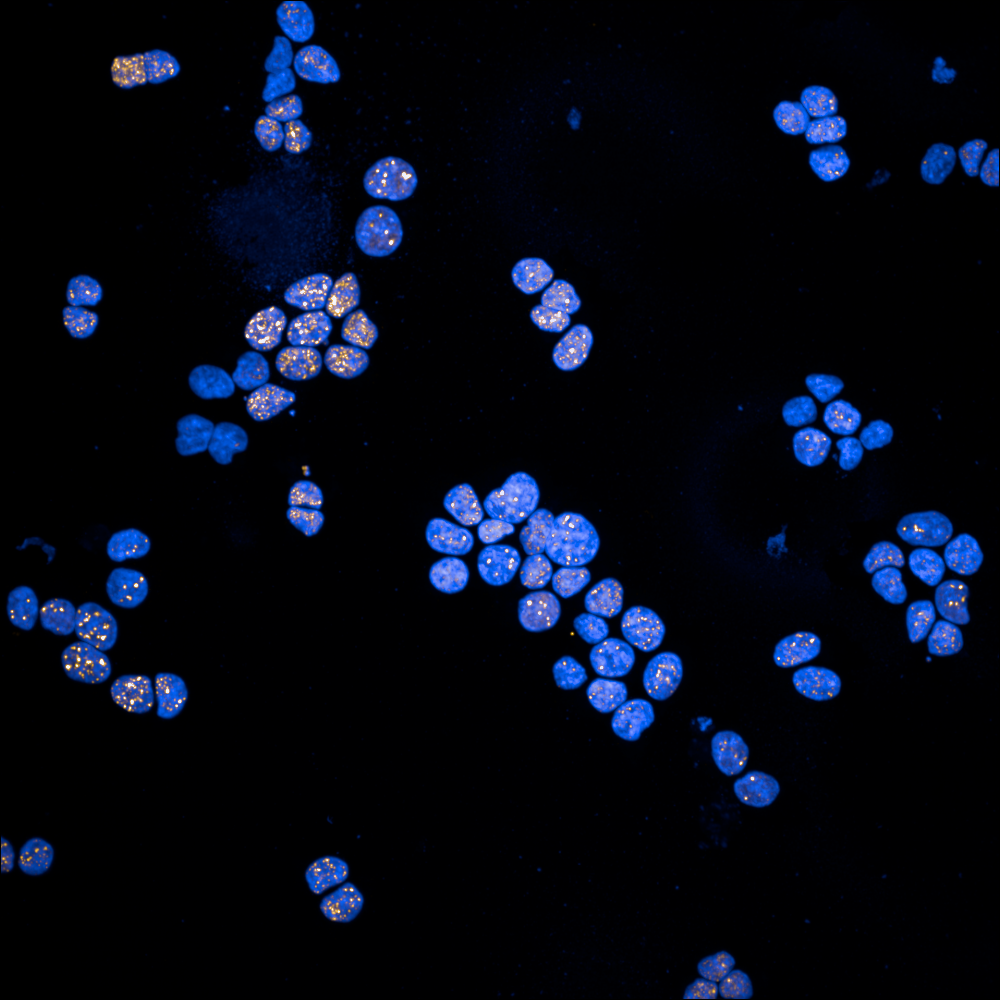

Supplement: Supplementary file 10 — Source data Fig. 6 [file 44319_2025_385_MOESM10_ESM.zip › Figure 6/6C/KO+ΔTOWER-DAC.png]

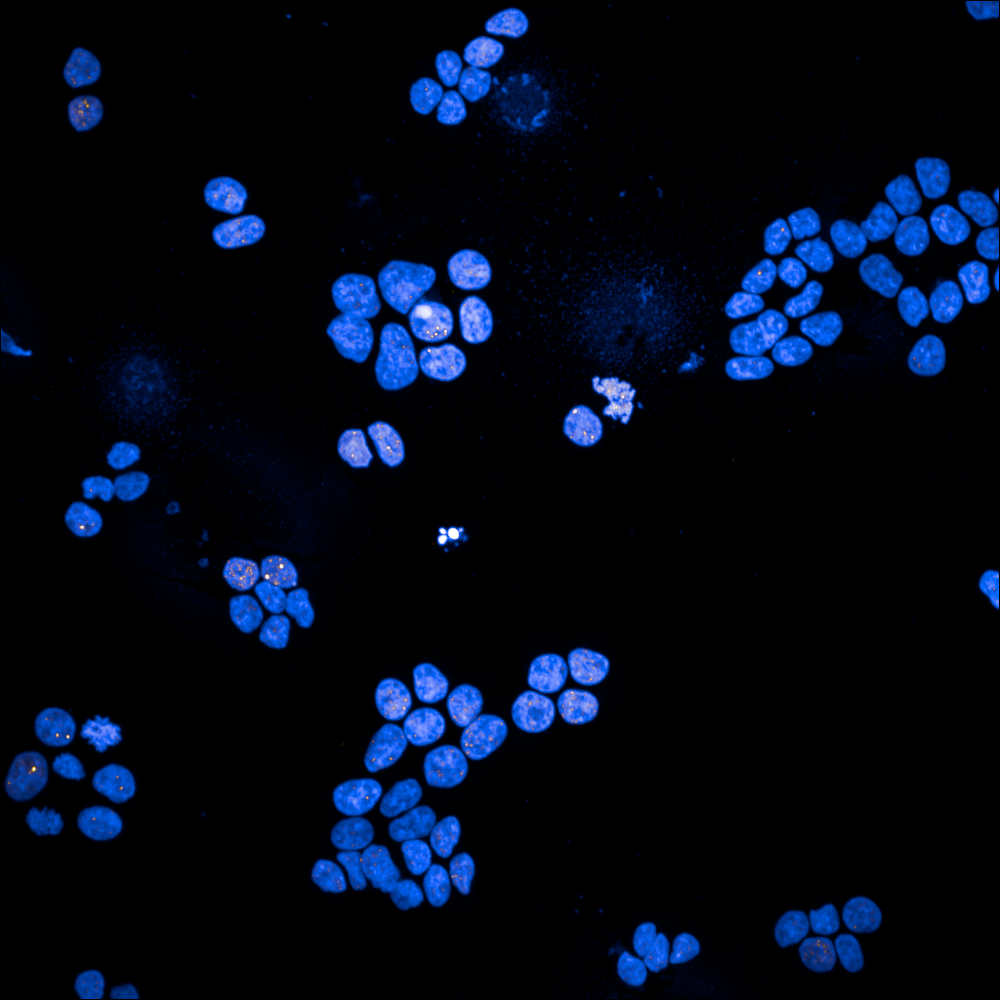

Supplement: Supplementary file 10 — Source data Fig. 6 [file 44319_2025_385_MOESM10_ESM.zip › Figure 6/6C/KO+ΔTOWER-DMSO.png]

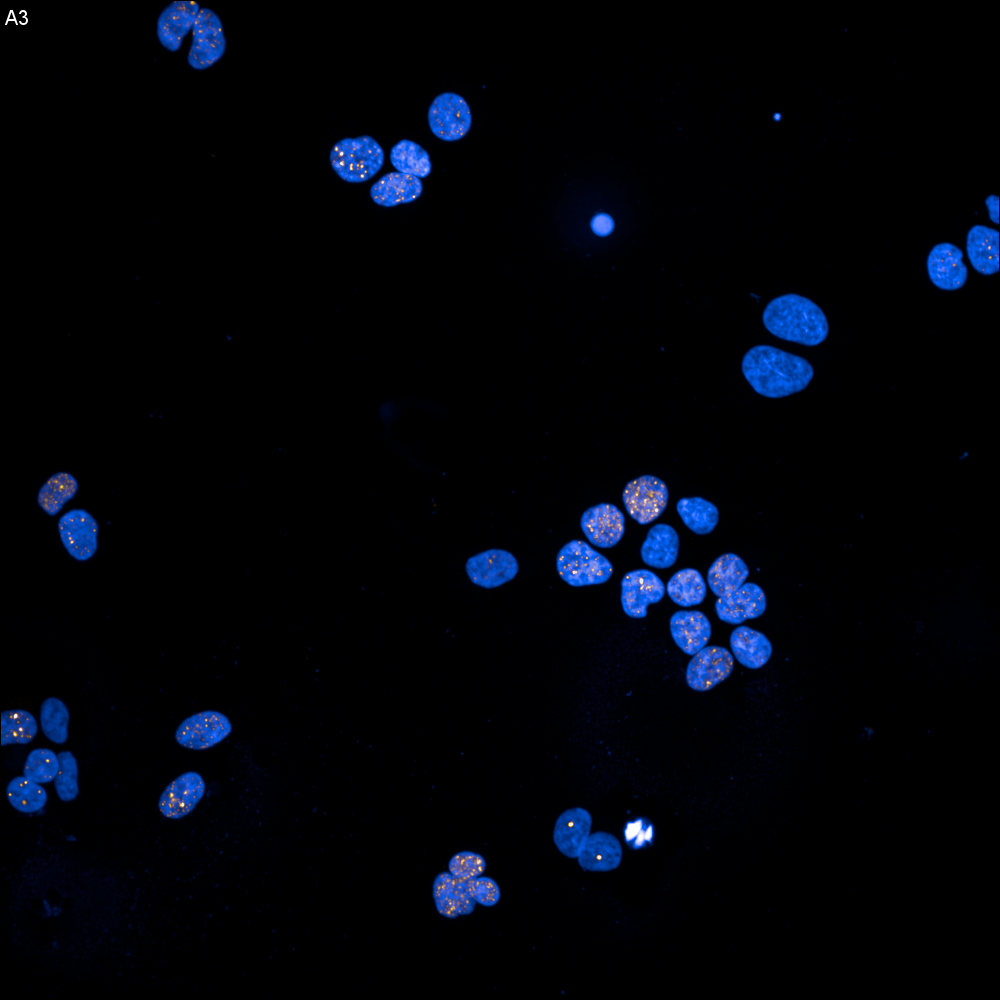

Supplement: Supplementary file 10 — Source data Fig. 6 [file 44319_2025_385_MOESM10_ESM.zip › Figure 6/6C/WT-DAC.png]

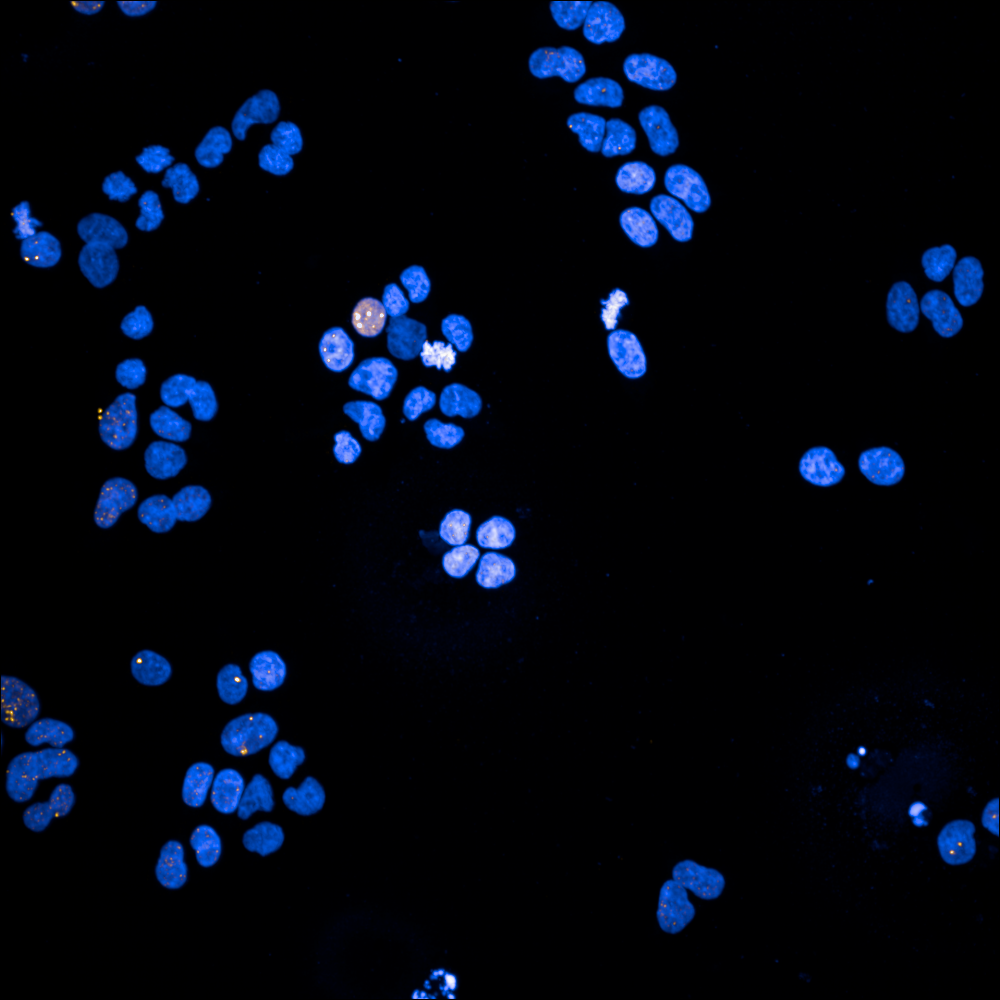

Supplement: Supplementary file 10 — Source data Fig. 6 [file 44319_2025_385_MOESM10_ESM.zip › Figure 6/6C/WT-DMSO.png]

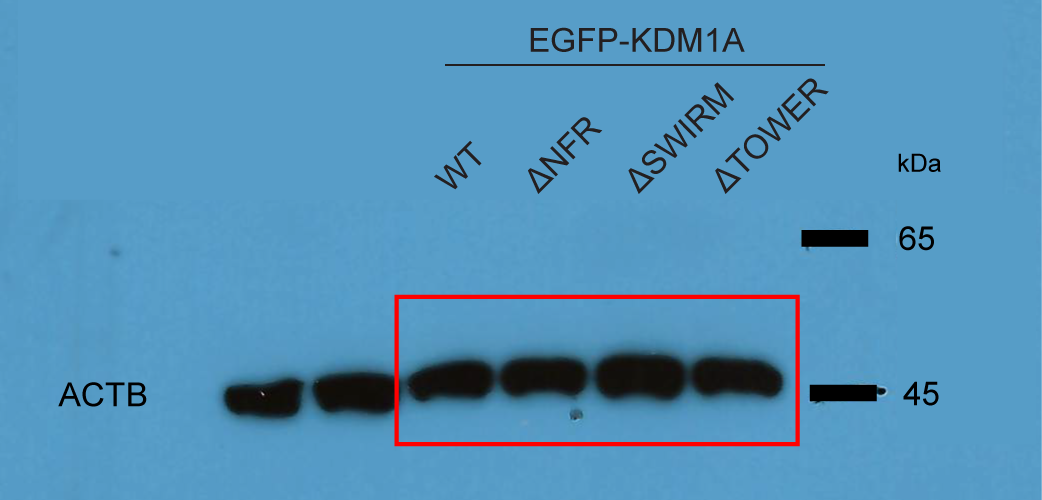

Supplement: Supplementary file 11 — Source data Fig. 7 [file 44319_2025_385_MOESM11_ESM.zip › Figure 7/7A/Western-ACTB.tif]

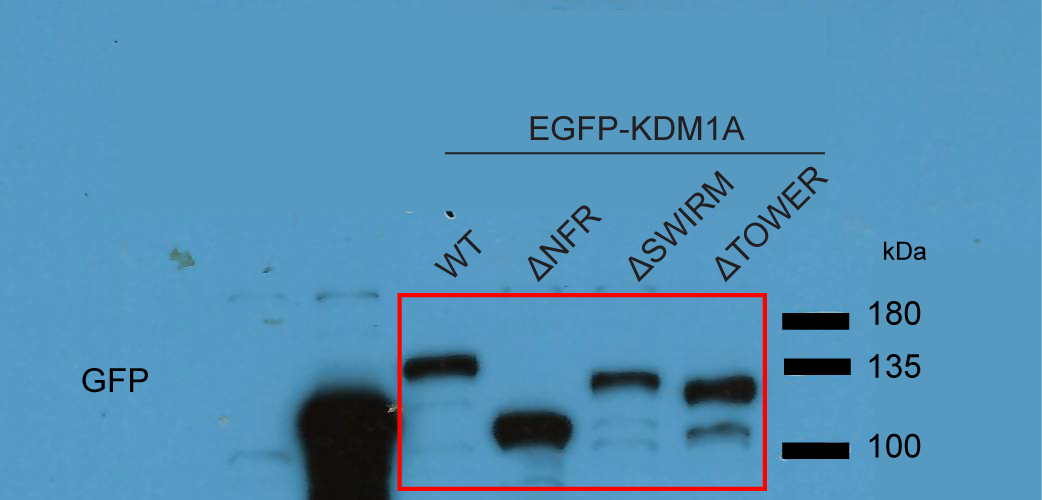

Supplement: Supplementary file 11 — Source data Fig. 7 [file 44319_2025_385_MOESM11_ESM.zip › Figure 7/7A/Western-GFP.tif]

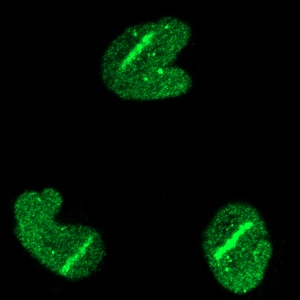

Supplement: Supplementary file 11 — Source data Fig. 7 [file 44319_2025_385_MOESM11_ESM.zip › Figure 7/7B/EGFP-KDM1A-WT-EGFP.jpg]

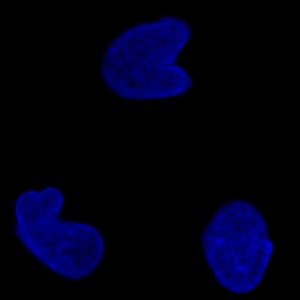

Supplement: Supplementary file 11 — Source data Fig. 7 [file 44319_2025_385_MOESM11_ESM.zip › Figure 7/7B/EGFP-KDM1A-WT-Hoechst33342.jpg]

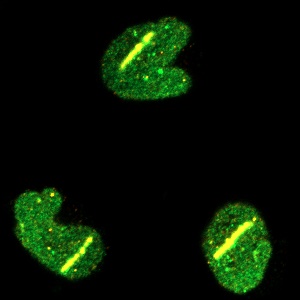

Supplement: Supplementary file 11 — Source data Fig. 7 [file 44319_2025_385_MOESM11_ESM.zip › Figure 7/7B/EGFP-KDM1A-WT-Merge.jpg]

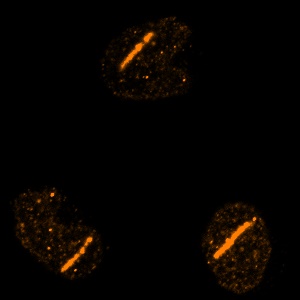

Supplement: Supplementary file 11 — Source data Fig. 7 [file 44319_2025_385_MOESM11_ESM.zip › Figure 7/7B/EGFP-KDM1A-WT-RPA32.jpg]

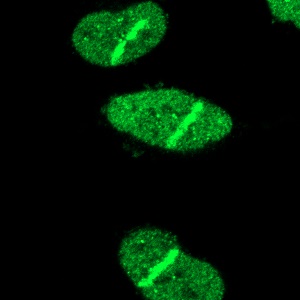

Supplement: Supplementary file 11 — Source data Fig. 7 [file 44319_2025_385_MOESM11_ESM.zip › Figure 7/7B/EGFP-KDM1A-ΔNFR-EGFP.jpg]

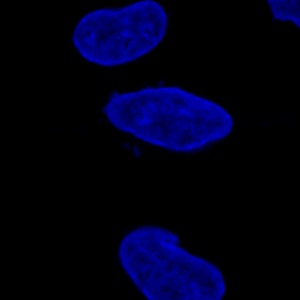

Supplement: Supplementary file 11 — Source data Fig. 7 [file 44319_2025_385_MOESM11_ESM.zip › Figure 7/7B/EGFP-KDM1A-ΔNFR-Hoechst33342.jpg]

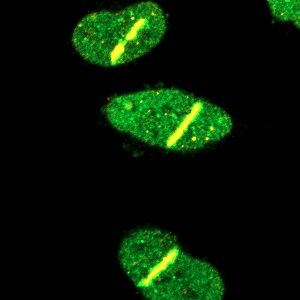

Supplement: Supplementary file 11 — Source data Fig. 7 [file 44319_2025_385_MOESM11_ESM.zip › Figure 7/7B/EGFP-KDM1A-ΔNFR-Merge.jpg]

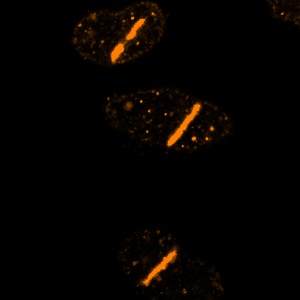

Supplement: Supplementary file 11 — Source data Fig. 7 [file 44319_2025_385_MOESM11_ESM.zip › Figure 7/7B/EGFP-KDM1A-ΔNFR-RPA32.jpg]

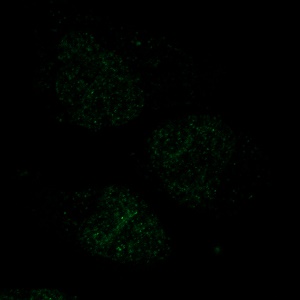

Supplement: Supplementary file 11 — Source data Fig. 7 [file 44319_2025_385_MOESM11_ESM.zip › Figure 7/7B/EGFP-KDM1A-ΔSWIRM-EGFP.jpg]

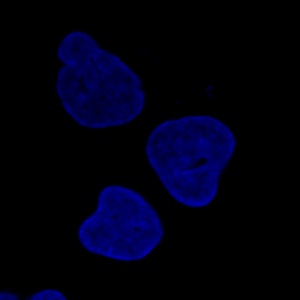

Supplement: Supplementary file 11 — Source data Fig. 7 [file 44319_2025_385_MOESM11_ESM.zip › Figure 7/7B/EGFP-KDM1A-ΔSWIRM-Hoechst33342.jpg]

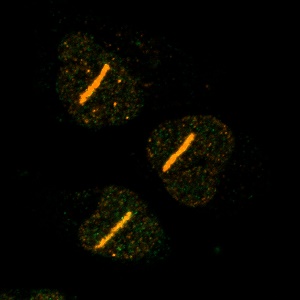

Supplement: Supplementary file 11 — Source data Fig. 7 [file 44319_2025_385_MOESM11_ESM.zip › Figure 7/7B/EGFP-KDM1A-ΔSWIRM-Merge.jpg]

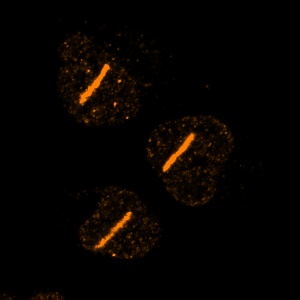

Supplement: Supplementary file 11 — Source data Fig. 7 [file 44319_2025_385_MOESM11_ESM.zip › Figure 7/7B/EGFP-KDM1A-ΔSWIRM-RPA32.jpg]

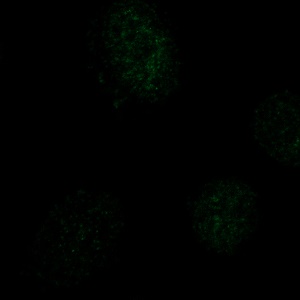

Supplement: Supplementary file 11 — Source data Fig. 7 [file 44319_2025_385_MOESM11_ESM.zip › Figure 7/7B/EGFP-KDM1A-ΔTOWER-EGFP.jpg]

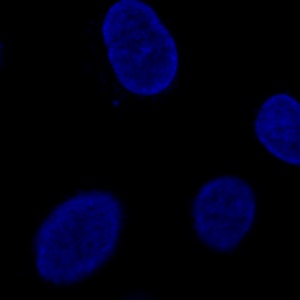

Supplement: Supplementary file 11 — Source data Fig. 7 [file 44319_2025_385_MOESM11_ESM.zip › Figure 7/7B/EGFP-KDM1A-ΔTOWER-Hoechst33342.jpg]

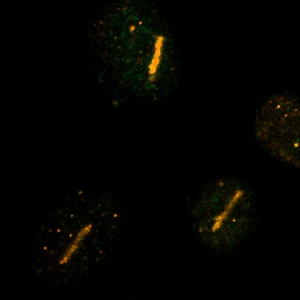

Supplement: Supplementary file 11 — Source data Fig. 7 [file 44319_2025_385_MOESM11_ESM.zip › Figure 7/7B/EGFP-KDM1A-ΔTOWER-Merge.jpg]

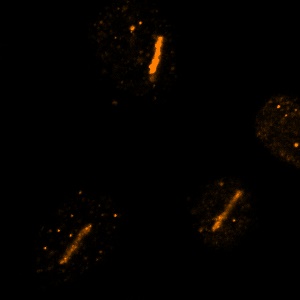

Supplement: Supplementary file 11 — Source data Fig. 7 [file 44319_2025_385_MOESM11_ESM.zip › Figure 7/7B/EGFP-KDM1A-ΔTOWER-RPA32.jpg]

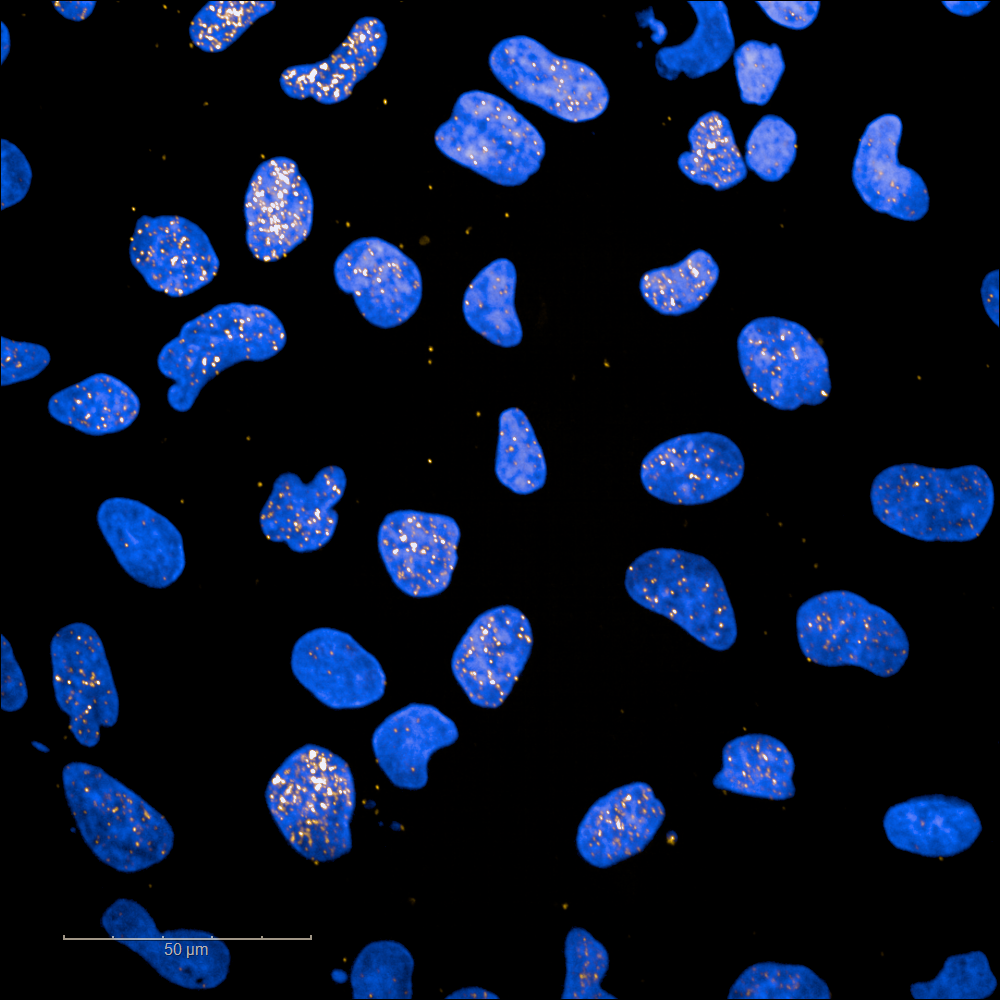

Supplement: Supplementary file 11 — Source data Fig. 7 [file 44319_2025_385_MOESM11_ESM.zip › Figure 7/7D/WT-DAC.png]

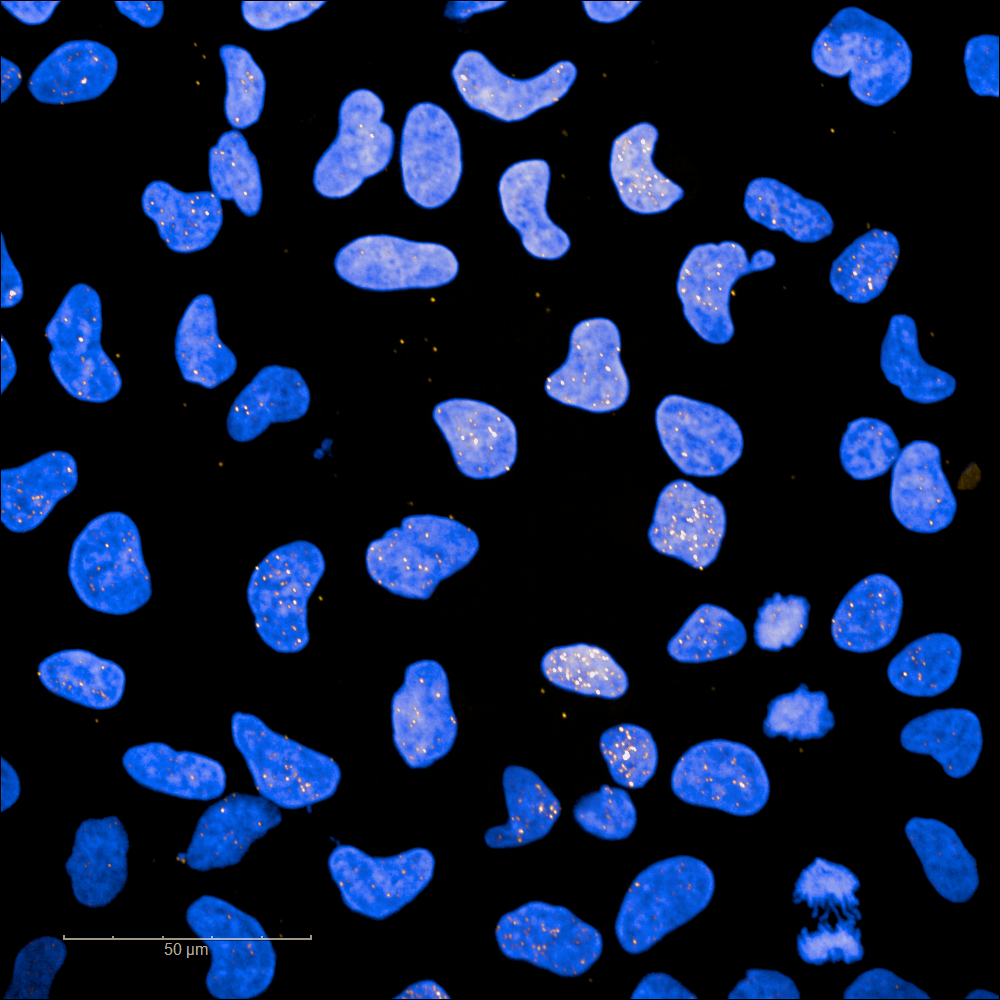

Supplement: Supplementary file 11 — Source data Fig. 7 [file 44319_2025_385_MOESM11_ESM.zip › Figure 7/7D/WT-DMSO.png]

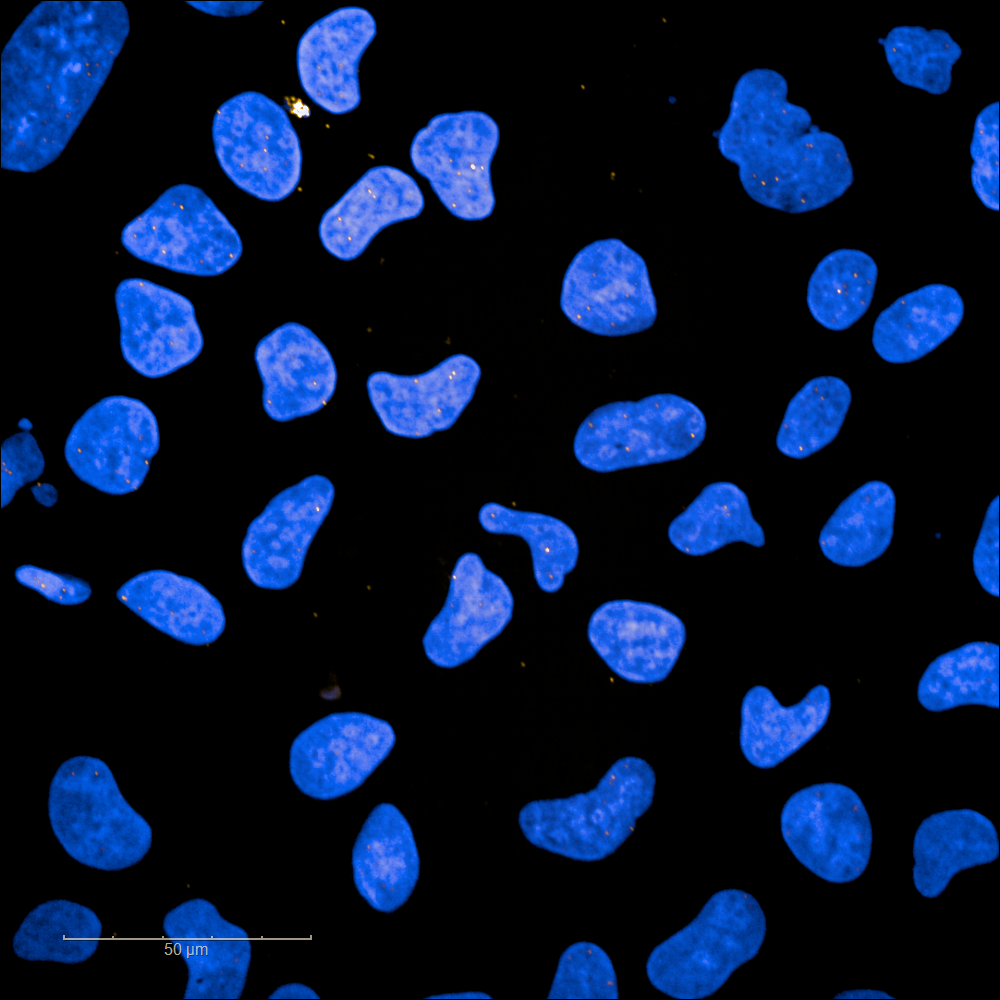

Supplement: Supplementary file 11 — Source data Fig. 7 [file 44319_2025_385_MOESM11_ESM.zip › Figure 7/7D/ΔSWIRM-DAC.png]

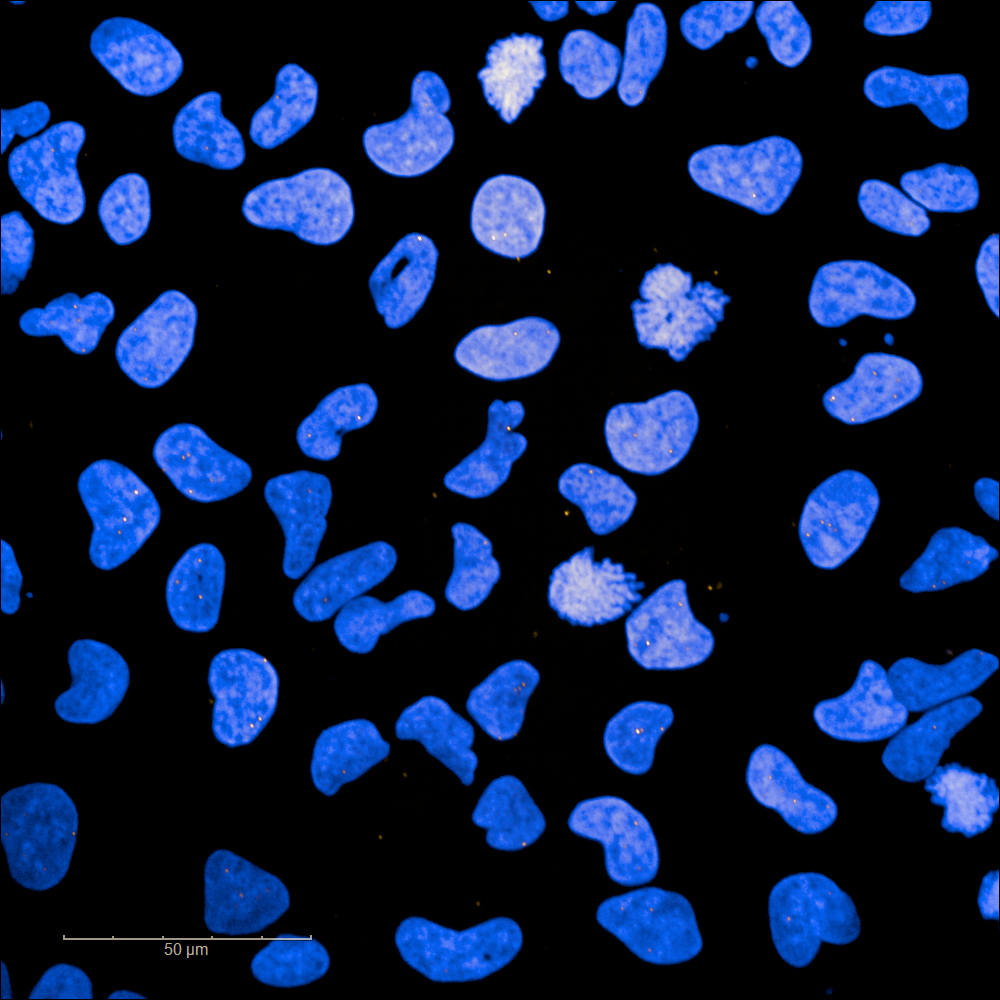

Supplement: Supplementary file 11 — Source data Fig. 7 [file 44319_2025_385_MOESM11_ESM.zip › Figure 7/7D/ΔSWIRM-DMSO.png]

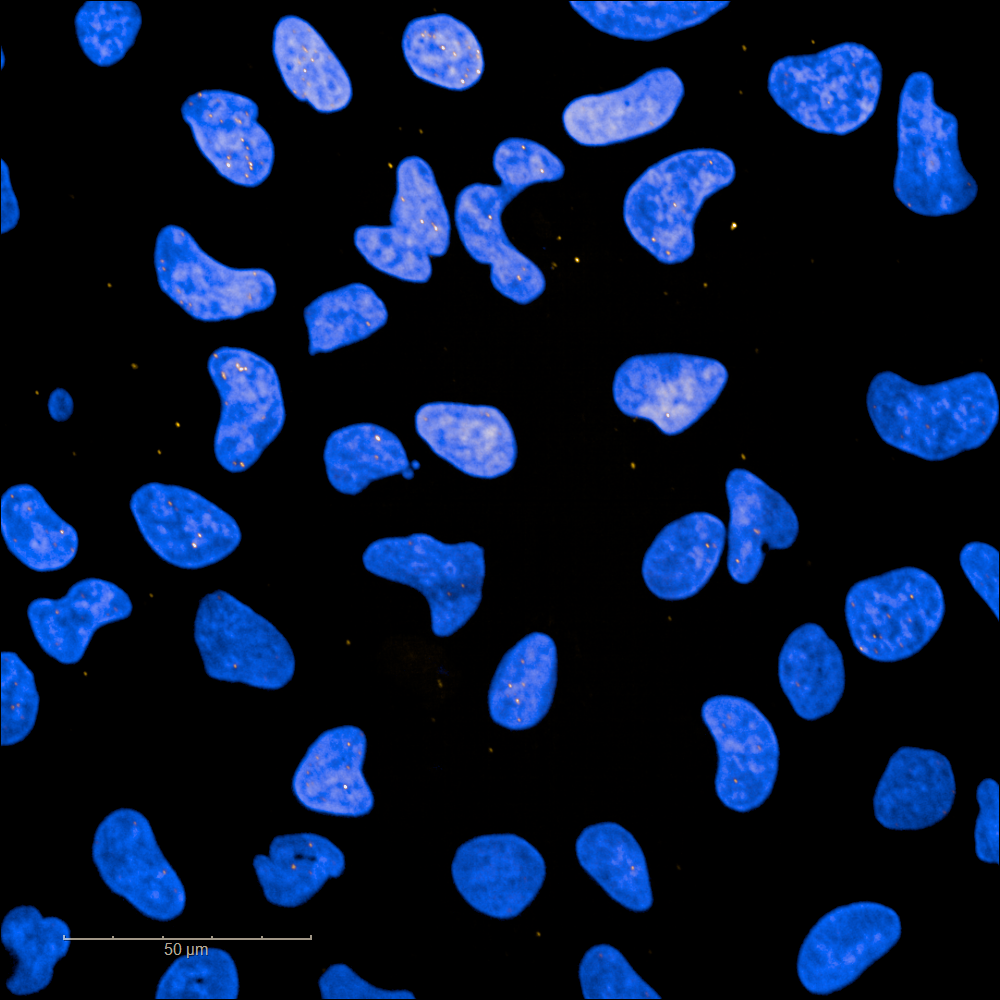

Supplement: Supplementary file 11 — Source data Fig. 7 [file 44319_2025_385_MOESM11_ESM.zip › Figure 7/7D/ΔTOWER-DAC.png]

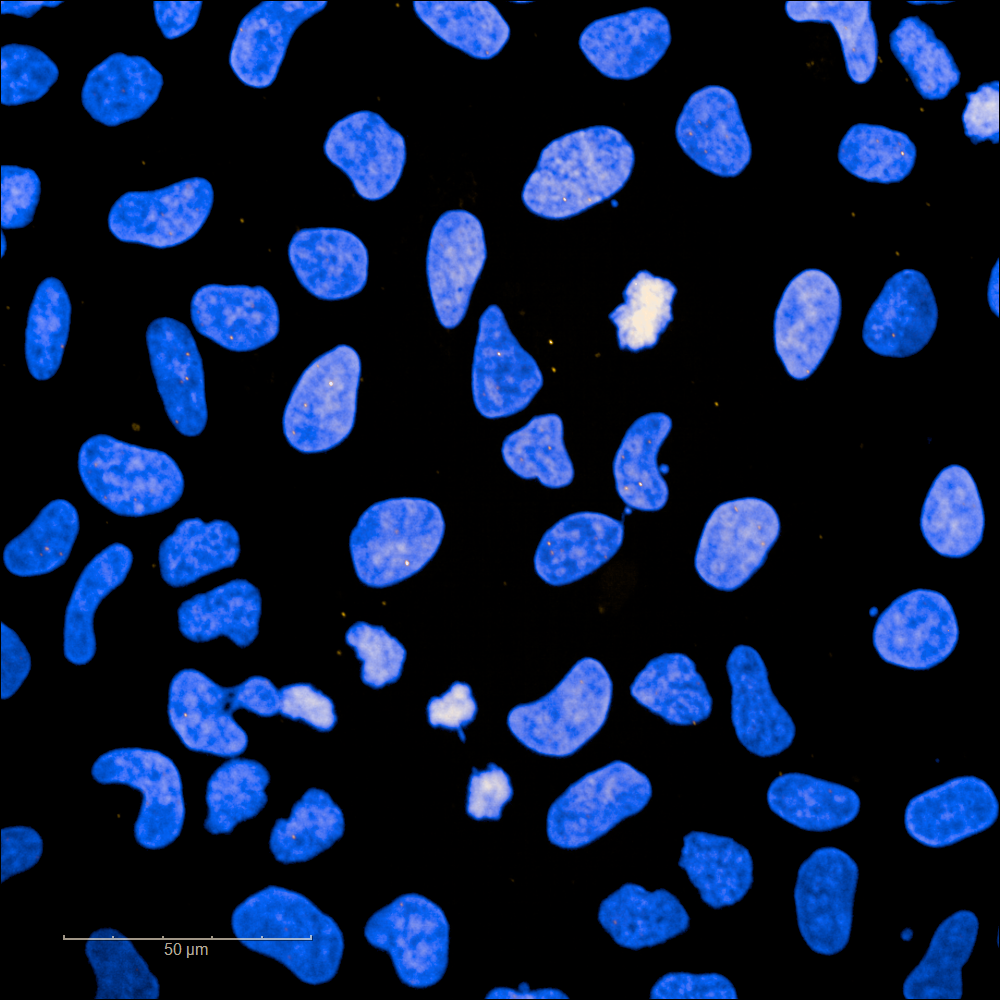

Supplement: Supplementary file 11 — Source data Fig. 7 [file 44319_2025_385_MOESM11_ESM.zip › Figure 7/7D/ΔTOWER-DMSO.png]

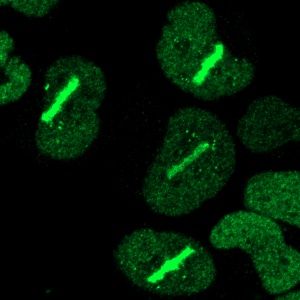

Supplement: Supplementary file 11 — Source data Fig. 7 [file 44319_2025_385_MOESM11_ESM.zip › Figure 7/7E/sgCTRL-EGFP.jpg]

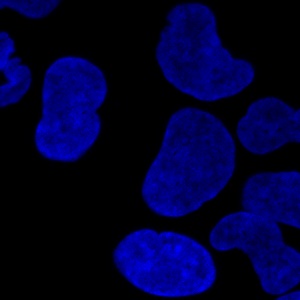

Supplement: Supplementary file 11 — Source data Fig. 7 [file 44319_2025_385_MOESM11_ESM.zip › Figure 7/7E/sgCTRL-Hoechst33342.jpg]

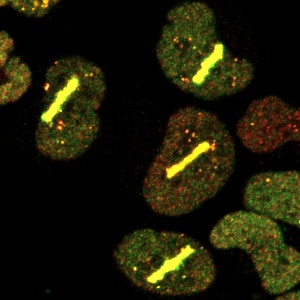

Supplement: Supplementary file 11 — Source data Fig. 7 [file 44319_2025_385_MOESM11_ESM.zip › Figure 7/7E/sgCTRL-Merge.jpg]

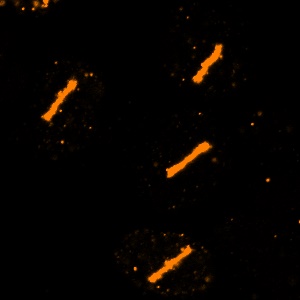

Supplement: Supplementary file 11 — Source data Fig. 7 [file 44319_2025_385_MOESM11_ESM.zip › Figure 7/7E/sgCTRL-RPA32.jpg]

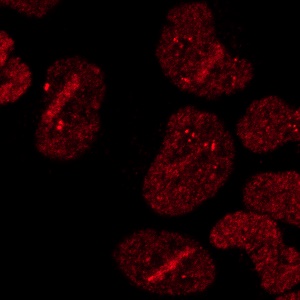

Supplement: Supplementary file 11 — Source data Fig. 7 [file 44319_2025_385_MOESM11_ESM.zip › Figure 7/7E/sgCTRL-ZMYM2.jpg]

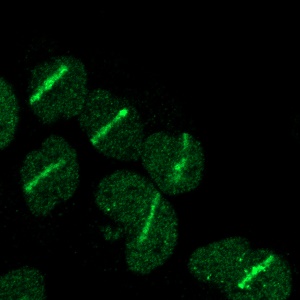

Supplement: Supplementary file 11 — Source data Fig. 7 [file 44319_2025_385_MOESM11_ESM.zip › Figure 7/7E/sgZMYM2 #1-EGFP.jpg]

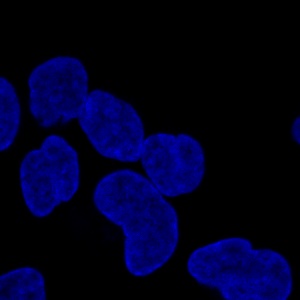

Supplement: Supplementary file 11 — Source data Fig. 7 [file 44319_2025_385_MOESM11_ESM.zip › Figure 7/7E/sgZMYM2 #1-Hoechst33342.jpg]

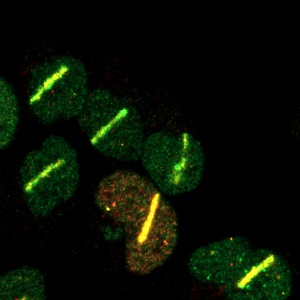

Supplement: Supplementary file 11 — Source data Fig. 7 [file 44319_2025_385_MOESM11_ESM.zip › Figure 7/7E/sgZMYM2 #1-Merge.jpg]

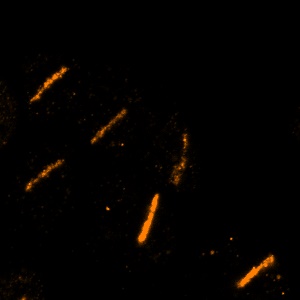

Supplement: Supplementary file 11 — Source data Fig. 7 [file 44319_2025_385_MOESM11_ESM.zip › Figure 7/7E/sgZMYM2 #1-RPA32.jpg]

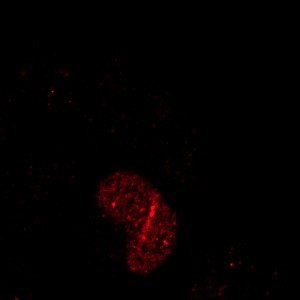

Supplement: Supplementary file 11 — Source data Fig. 7 [file 44319_2025_385_MOESM11_ESM.zip › Figure 7/7E/sgZMYM2 #1-ZMYM2.jpg]

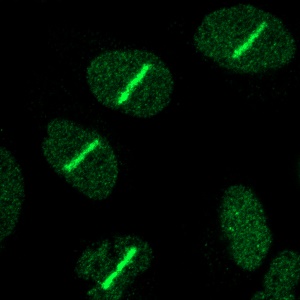

Supplement: Supplementary file 11 — Source data Fig. 7 [file 44319_2025_385_MOESM11_ESM.zip › Figure 7/7E/sgZMYM2 #2-EGFP.jpg]

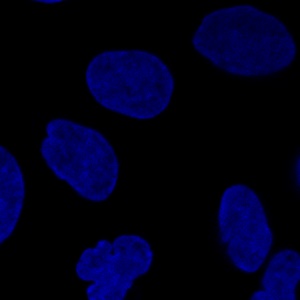

Supplement: Supplementary file 11 — Source data Fig. 7 [file 44319_2025_385_MOESM11_ESM.zip › Figure 7/7E/sgZMYM2 #2-Hoechst33342.jpg]

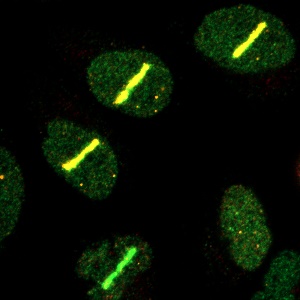

Supplement: Supplementary file 11 — Source data Fig. 7 [file 44319_2025_385_MOESM11_ESM.zip › Figure 7/7E/sgZMYM2 #2-Merge.jpg]

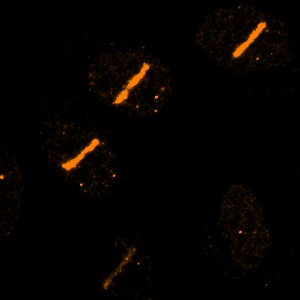

Supplement: Supplementary file 11 — Source data Fig. 7 [file 44319_2025_385_MOESM11_ESM.zip › Figure 7/7E/sgZMYM2 #2-RPA32.jpg]

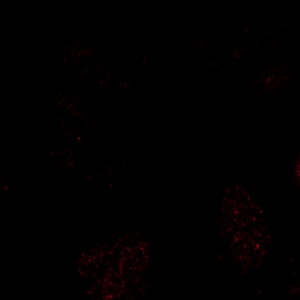

Supplement: Supplementary file 11 — Source data Fig. 7 [file 44319_2025_385_MOESM11_ESM.zip › Figure 7/7E/sgZMYM2 #2-ZMYM2.jpg]

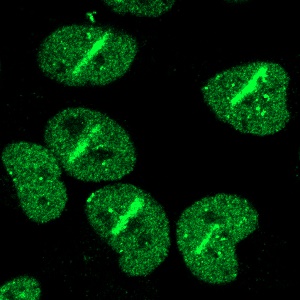

Supplement: Supplementary file 11 — Source data Fig. 7 [file 44319_2025_385_MOESM11_ESM.zip › Figure 7/7G/sgCTRL-EGFP.jpg]

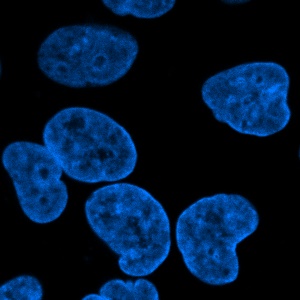

Supplement: Supplementary file 11 — Source data Fig. 7 [file 44319_2025_385_MOESM11_ESM.zip › Figure 7/7G/sgCTRL-Hoechst33342.jpg]

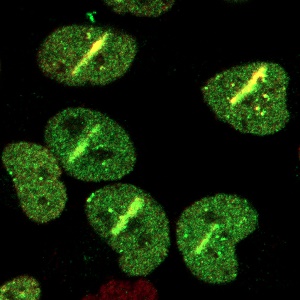

Supplement: Supplementary file 11 — Source data Fig. 7 [file 44319_2025_385_MOESM11_ESM.zip › Figure 7/7G/sgCTRL-Merge.jpg]

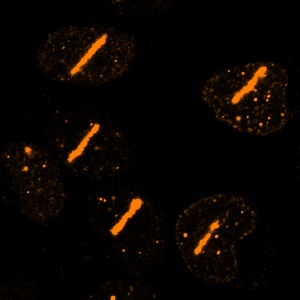

Supplement: Supplementary file 11 — Source data Fig. 7 [file 44319_2025_385_MOESM11_ESM.zip › Figure 7/7G/sgCTRL-RPA32.jpg]

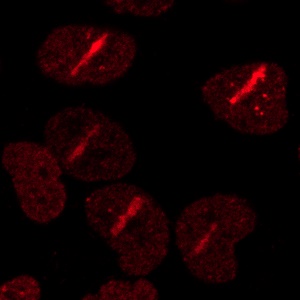

Supplement: Supplementary file 11 — Source data Fig. 7 [file 44319_2025_385_MOESM11_ESM.zip › Figure 7/7G/sgCTRL-ZMYM3.jpg]

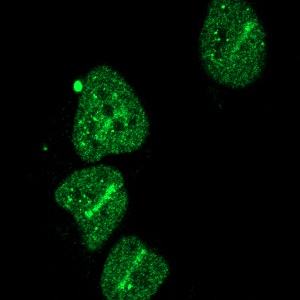

Supplement: Supplementary file 11 — Source data Fig. 7 [file 44319_2025_385_MOESM11_ESM.zip › Figure 7/7G/sgZMYM3 #1-EGFP.jpg]

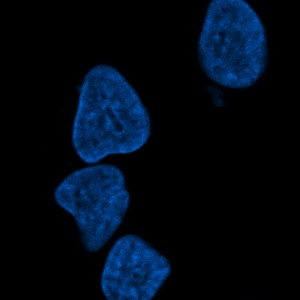

Supplement: Supplementary file 11 — Source data Fig. 7 [file 44319_2025_385_MOESM11_ESM.zip › Figure 7/7G/sgZMYM3 #1-Hoechst33342.jpg]

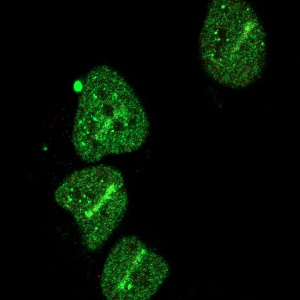

Supplement: Supplementary file 11 — Source data Fig. 7 [file 44319_2025_385_MOESM11_ESM.zip › Figure 7/7G/sgZMYM3 #1-Merge.jpg]

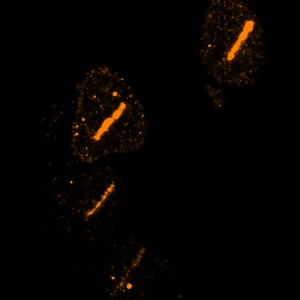

Supplement: Supplementary file 11 — Source data Fig. 7 [file 44319_2025_385_MOESM11_ESM.zip › Figure 7/7G/sgZMYM3 #1-RPA32.jpg]

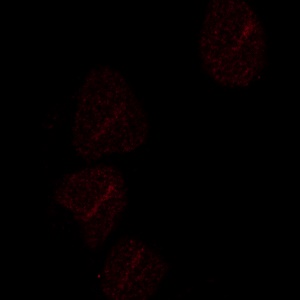

Supplement: Supplementary file 11 — Source data Fig. 7 [file 44319_2025_385_MOESM11_ESM.zip › Figure 7/7G/sgZMYM3 #1-ZMYM3.jpg]

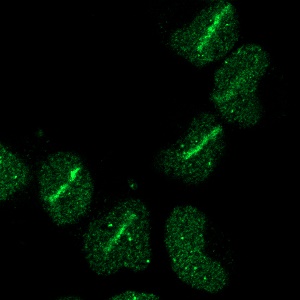

Supplement: Supplementary file 11 — Source data Fig. 7 [file 44319_2025_385_MOESM11_ESM.zip › Figure 7/7G/sgZMYM3 #2-EGFP.jpg]

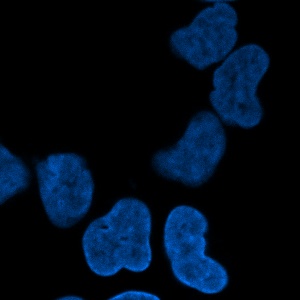

Supplement: Supplementary file 11 — Source data Fig. 7 [file 44319_2025_385_MOESM11_ESM.zip › Figure 7/7G/sgZMYM3 #2-Hoechst33342.jpg]

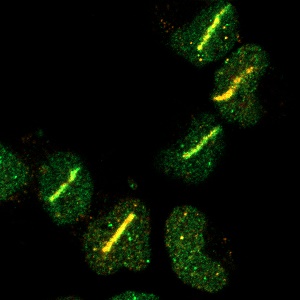

Supplement: Supplementary file 11 — Source data Fig. 7 [file 44319_2025_385_MOESM11_ESM.zip › Figure 7/7G/sgZMYM3 #2-Merge.jpg]

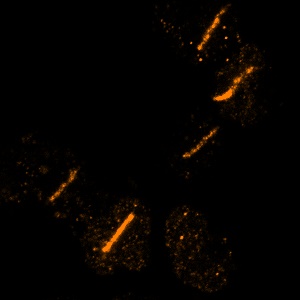

Supplement: Supplementary file 11 — Source data Fig. 7 [file 44319_2025_385_MOESM11_ESM.zip › Figure 7/7G/sgZMYM3 #2-RPA32.jpg]

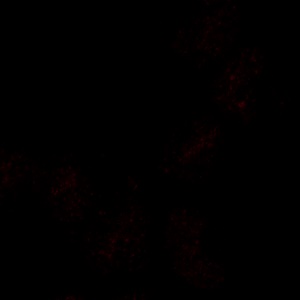

Supplement: Supplementary file 11 — Source data Fig. 7 [file 44319_2025_385_MOESM11_ESM.zip › Figure 7/7G/sgZMYM3 #2-ZMYM3.jpg]

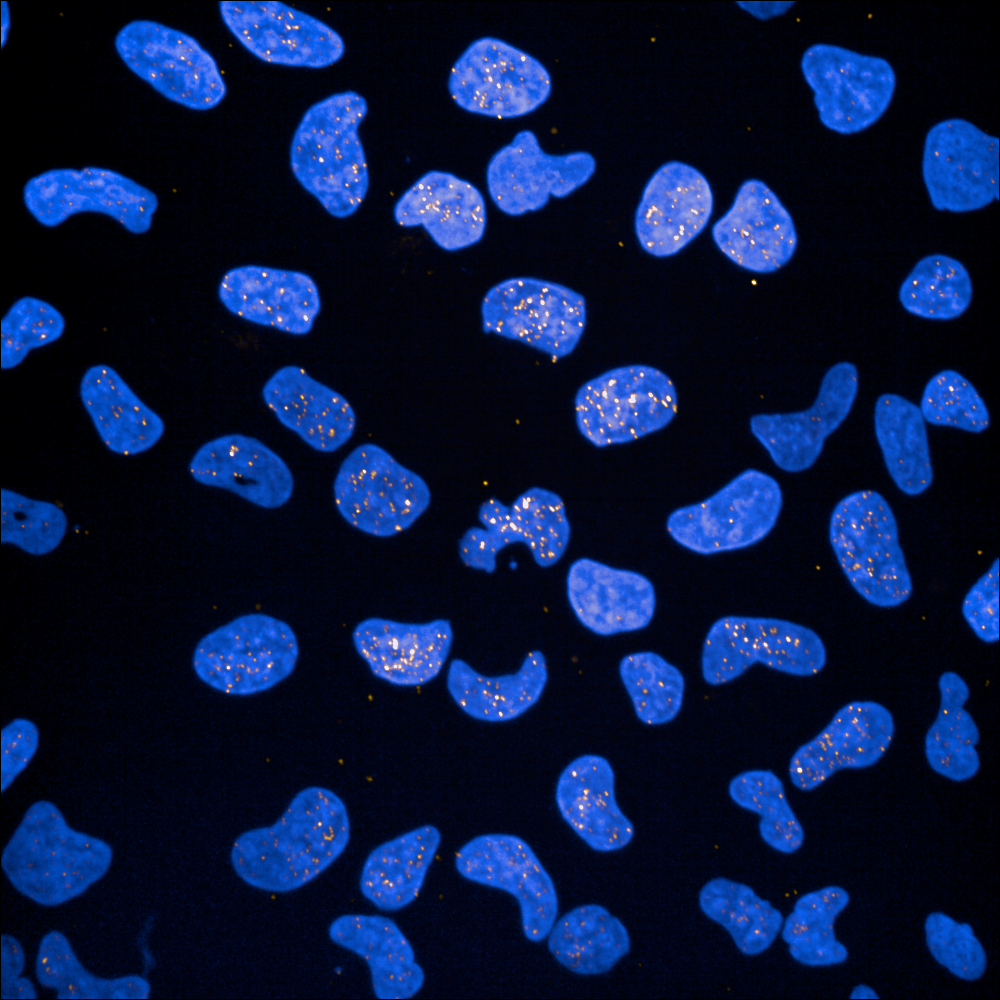

Supplement: Supplementary file 11 — Source data Fig. 7 [file 44319_2025_385_MOESM11_ESM.zip › Figure 7/7I/sgCTRL-DAC.png]

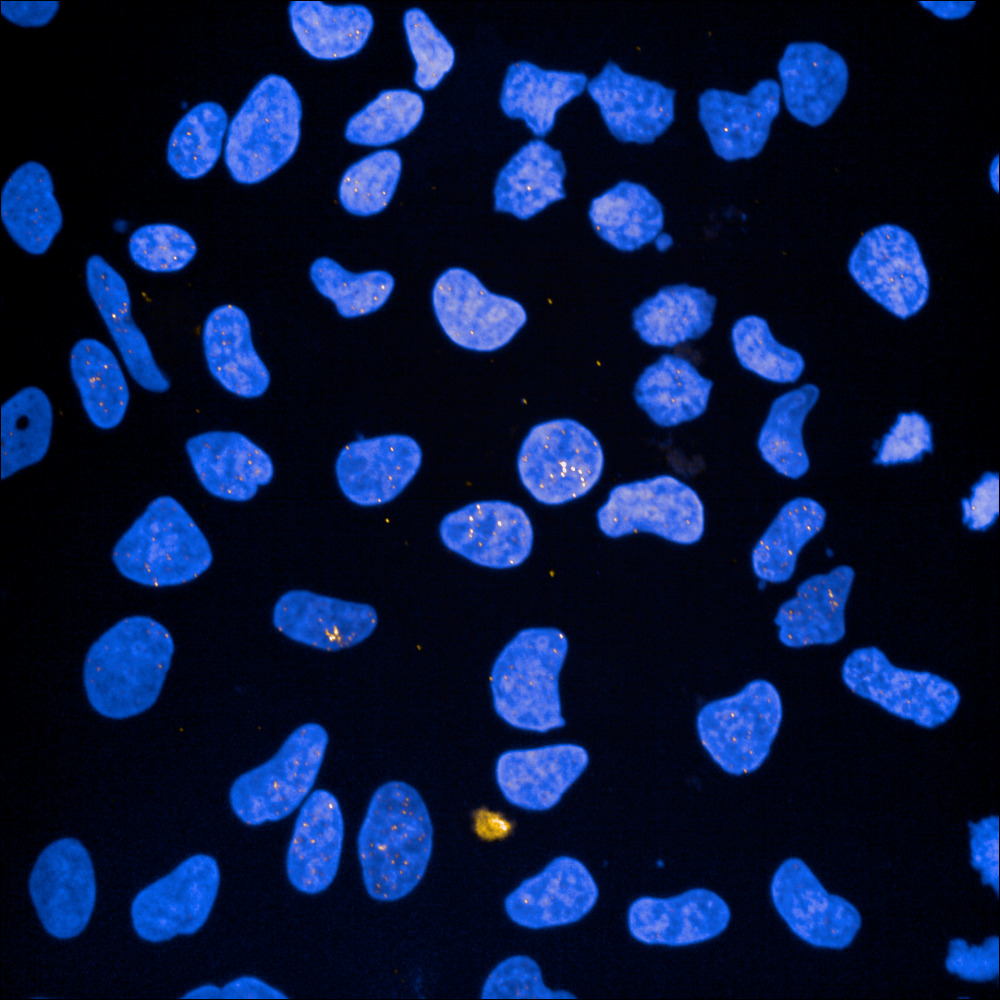

Supplement: Supplementary file 11 — Source data Fig. 7 [file 44319_2025_385_MOESM11_ESM.zip › Figure 7/7I/sgCTRL-DMSO.png]
