# Supplementary figures and images for: A CRISPR-Cas9 screen reveals genetic determinants of the cellular response to decitabine (part 2 of 2)
Source: EMBO Rep. 2025 Feb 10;26(6):1528–65. doi: 10.1038/s44319-025-00385-w (PMC11933316; doi:10.1038/s44319-025-00385-w)

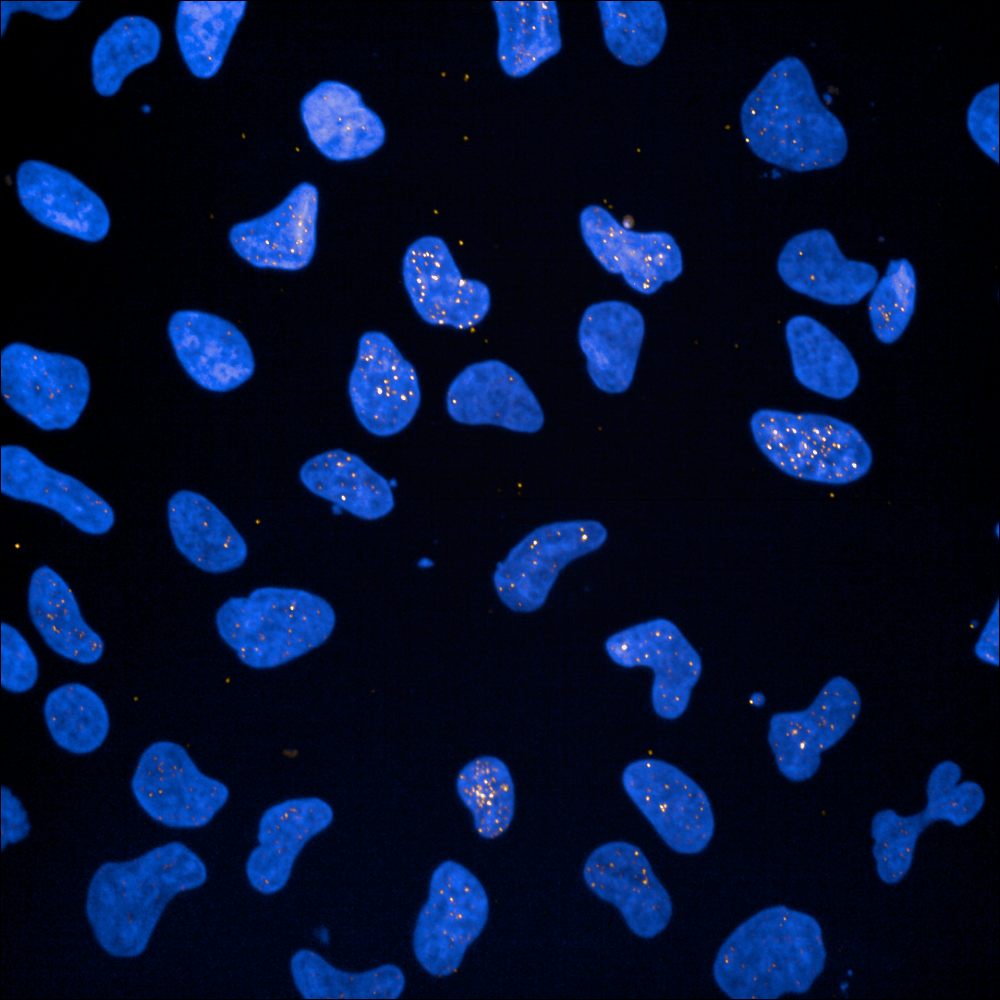

Supplement: Supplementary file 11 — Source data Fig. 7 [file 44319_2025_385_MOESM11_ESM.zip › Figure 7/7I/sgZMYM3-1-DAC.png]

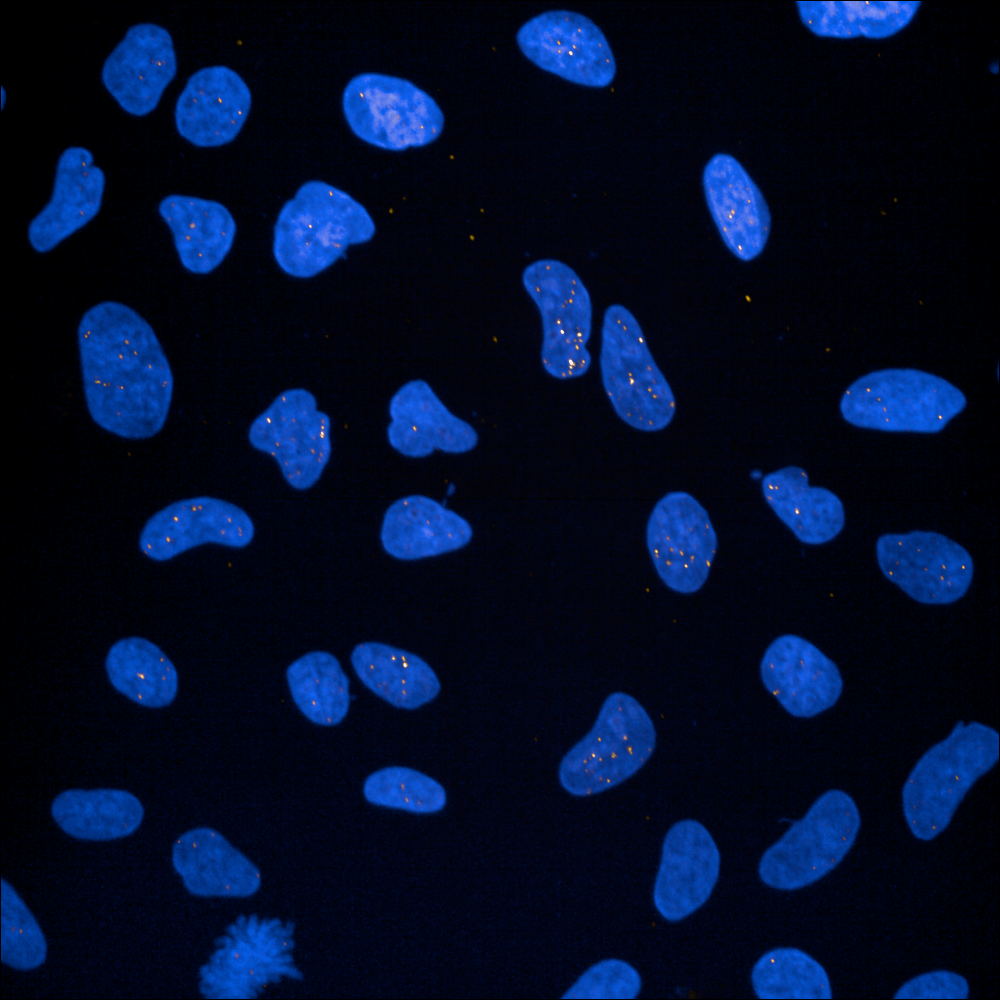

Supplement: Supplementary file 11 — Source data Fig. 7 [file 44319_2025_385_MOESM11_ESM.zip › Figure 7/7I/sgZMYM3-1-DMSO.png]

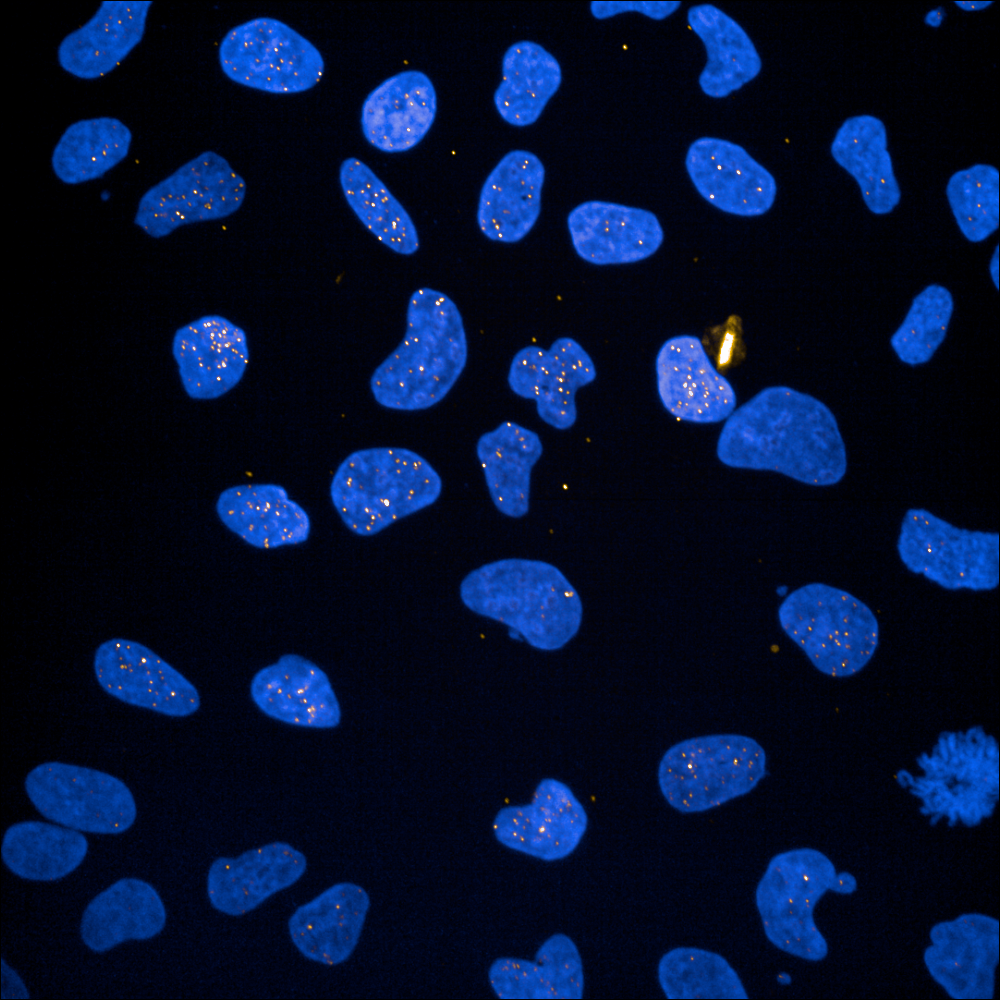

Supplement: Supplementary file 11 — Source data Fig. 7 [file 44319_2025_385_MOESM11_ESM.zip › Figure 7/7I/sgZMYM3-2-DAC.png]

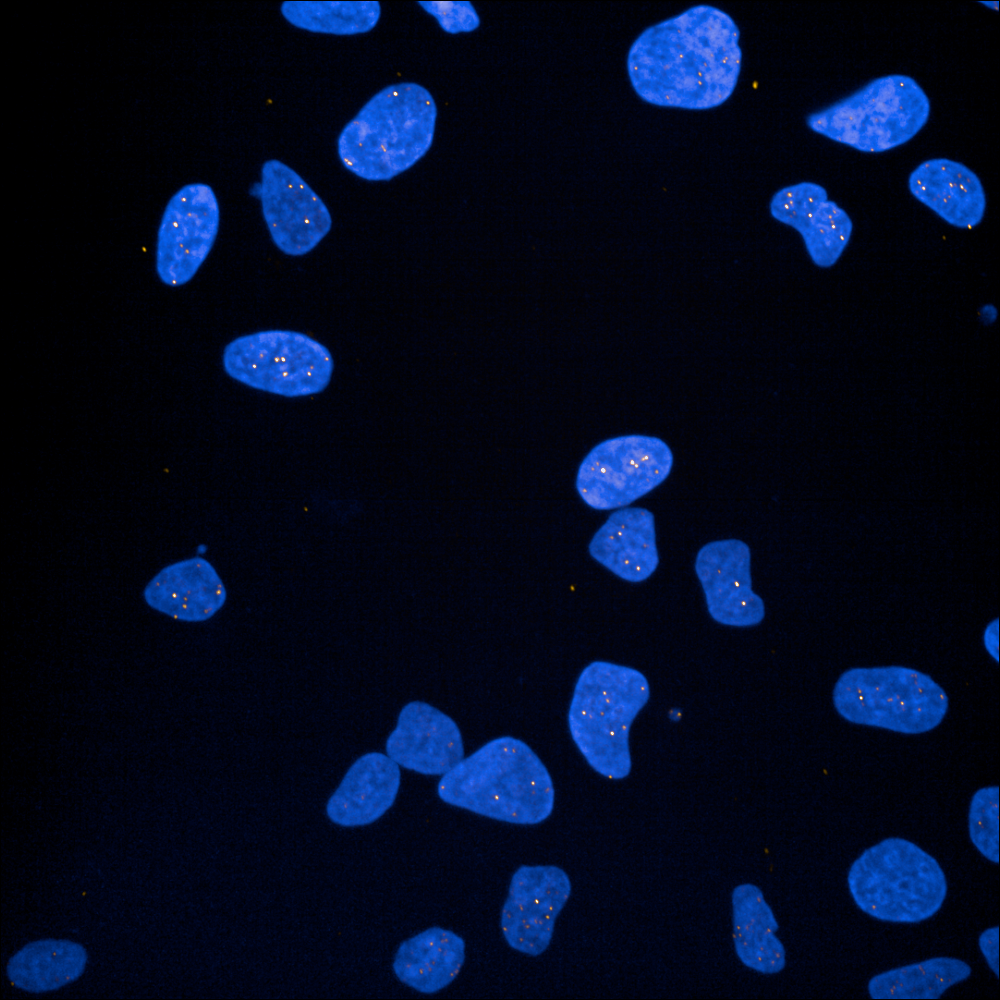

Supplement: Supplementary file 11 — Source data Fig. 7 [file 44319_2025_385_MOESM11_ESM.zip › Figure 7/7I/sgZMYM3-2-DMSO.png]

Source data for Fig. EV6 C (previous Fig. EV4 C)

Shorter exposure

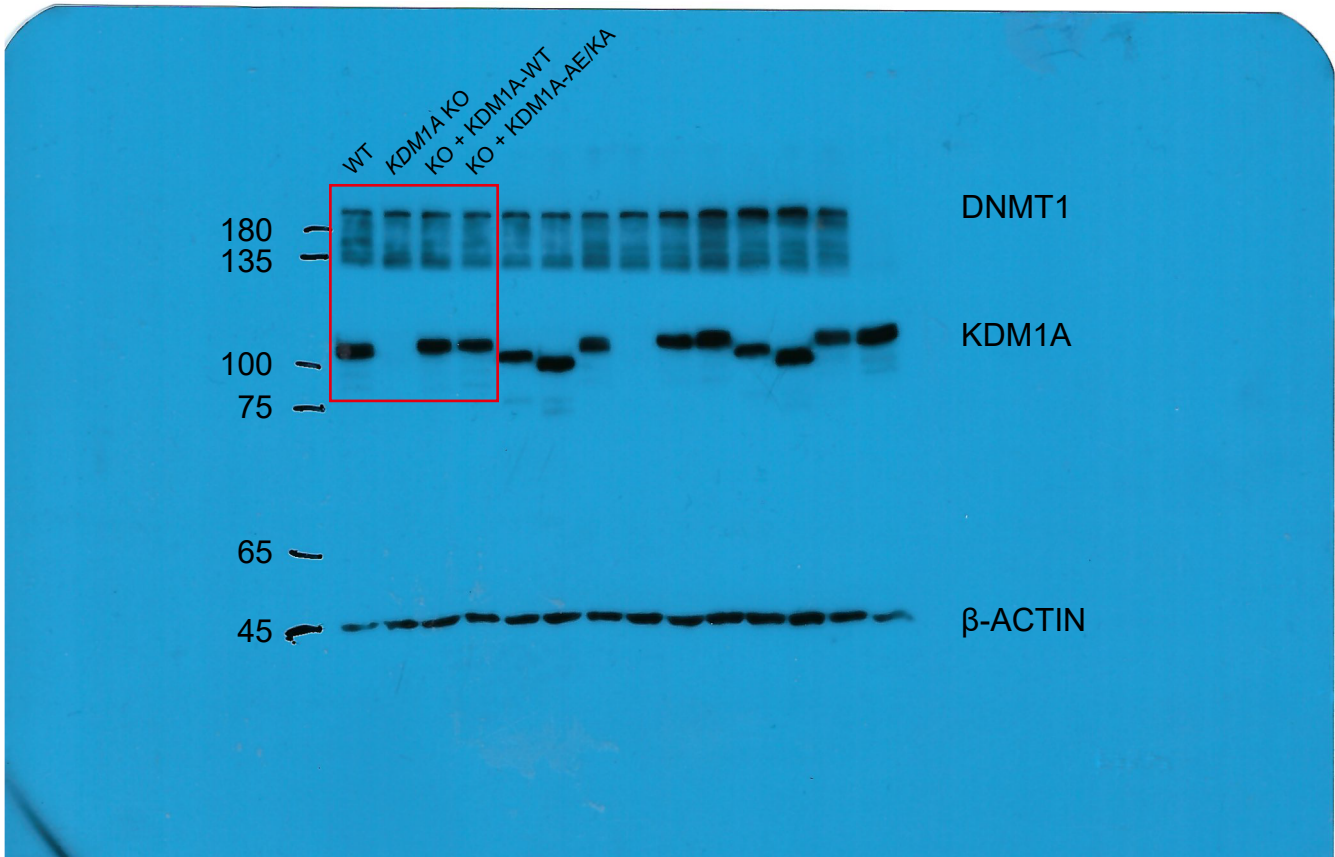

Longer exposure

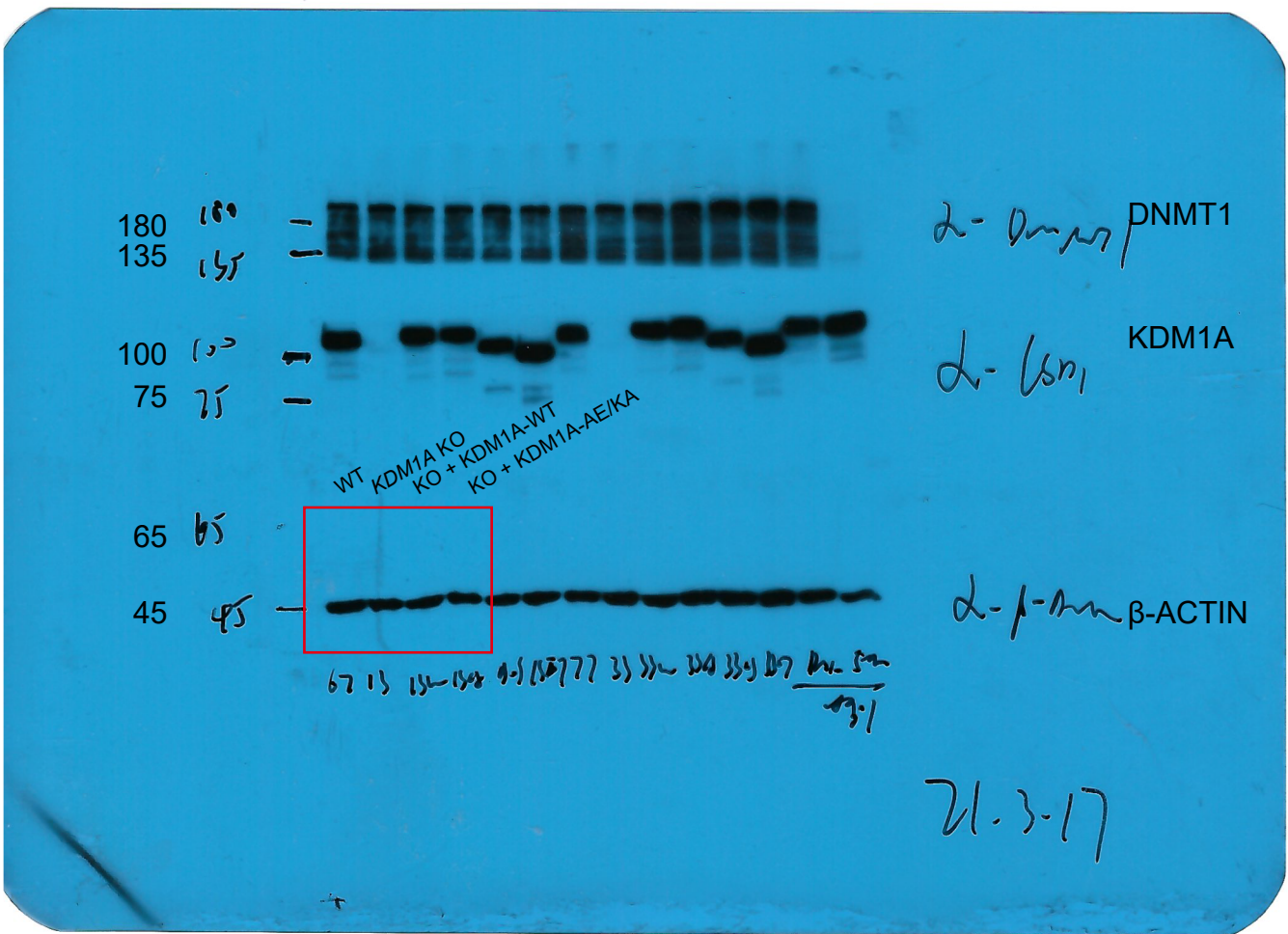

Supplement: Supplementary file 12 — Figure Source Data EV6C [file 44319_2025_385_MOESM12_ESM.zip › Source data for Fig EV6 C (previous Fig EV4 C)/Source data for Fig EV6 C (previous Fig EV4 C).pdf]
