# Supplementary material for: A CRISPR-Cas9 screen reveals genetic determinants of the cellular response to decitabine
Source: EMBO Rep. 2025 Feb 10;26(6):1528–65. doi: 10.1038/s44319-025-00385-w (PMC11933316; doi:10.1038/s44319-025-00385-w)
Supplement: Supplementary file 13 — Expanded View Figures [file 44319_2025_385_MOESM13_ESM.pdf]

Expanded View Figures

Figure EV1. Genome-wide CRISPR-Cas9 screen results analysis to identify gene networks that regulating cellular response to DAC.

(**A**) Immunoblot analysis showing that FLAG-tagged Cas9 protein was stably expressed in HAP1-Cas9-F5 cells, with β-ACTIN as a loading control. (**B**) Dose–response curves of HAP1-Cas9-F5 cells transduced with either control sgRNA (sgCTRL) or *DCK*-targeting sgRNA (sg*DCK*) upon DAC treatment at indicated concentrations. Cell viability was measured by CellTiter-Glo after 3 days of DAC treatment. Data are presented with ± SEM. Experiments performed in duplicates. The *p* value was determined using nonlinear regression followed by the extra sum-of-squares F test. (**C**) Ranked genes from CRISPR screen determined by comparing DAC to DMSO treatment. Genes are ranked by the averaged differential β scores at indicated time points. Negatively and positively selected genes are labeled with blue and red, respectively. (**D**) KEGG pathway enrichment of 6 clusters in Fig. 1D. The *p* values were determined using the hypergeometric distribution and adjusted by the Benjamini-Hochberg (BH) procedure. (**E**) STRING network of Cluster 1 hits. (**F**) STRING network of Cluster 6 hits.

© The Author(s)

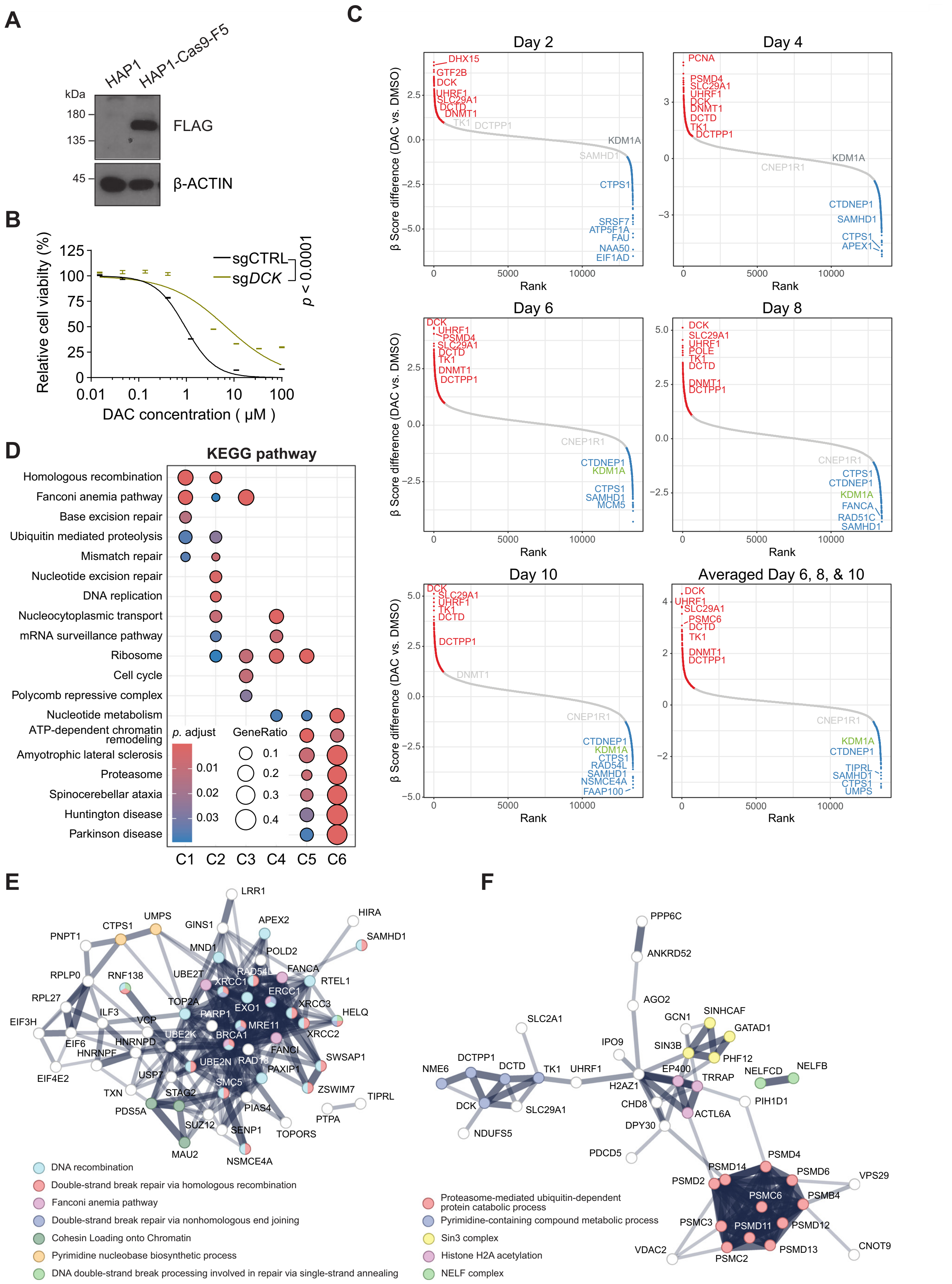
A
HAP1
HAP1-Cas9-F5
kDa
180
135
45
FLAG
β-ACTIN
B
Relative cell viability (%)
sgCTRL
sgDCK
p < 0.0001
DAC concentration (μM)
C
Day 2
Day 4
Day 6
Day 8
Day 10
Averaged Day 6, 8, & 10
β Score difference (DAC vs. DMSO)
Rank
D
KEGG pathway
Homologous recombination
Fanconi anemia pathway
Base excision repair
Ubiquitin mediated proteolysis
Mismatch repair
Nucleotide excision repair
DNA replication
Nucleocytoplasmic transport
mRNA surveillance pathway
Ribosome
Cell cycle
Polycomb repressive complex
Nucleotide metabolism
ATP-dependent chromatin remodeling
Amyotrophic lateral sclerosis
Proteasome
Spinocerebellar ataxia
Huntington disease
Parkinson disease
p. adjust
GeneRatio
C1 C2 C3 C4 C5 C6
E
DNA recombination
Double-strand break repair via homologous recombination
Fanconi anemia pathway
Double-strand break repair via nonhomologous end joining
Cohesin Loading onto Chromatin
Pyrimidine nucleobase biosynthetic process
DNA double-strand break processing involved in repair via single-strand annealing
F
Proteasome-mediated ubiquitin-dependent protein catabolic process
Pyrimidine-containing compound metabolic process
Sin3 complex
Histone H2A acetylation
NELF complex

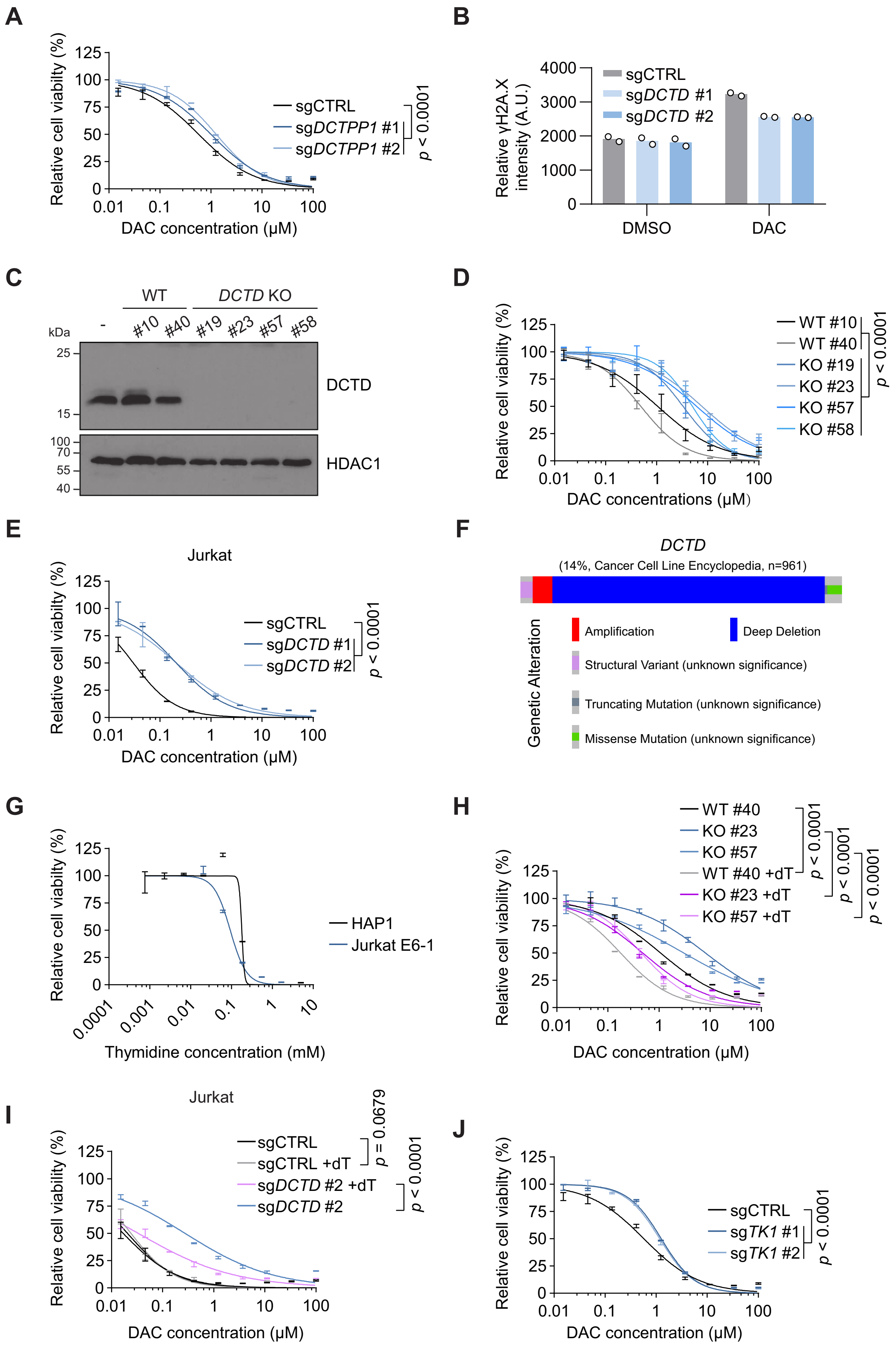
A
Relative cell viabilty (%)
DAC concentration (μM)
sgCTRL
sgDCTPP1 #1
sgDCTPP1 #2
p < 0.0001
B
Relative γH2A.X intensity (A.U.)
sgCTRL
sgDCTD #1
sgDCTD #2
DMSO
DAC
C
WT
DCTD KO
kDa
-
#10
#40
#19
#23
#57
#58
DCTD
HDAC1
D
Relative cell viabilty (%)
DAC concentrations (μM)
WT #10
WT #40
KO #19
KO #23
KO #57
KO #58
p < 0.0001
E
Jurkat
Relative cell viabilty (%)
DAC concentration (μM)
sgCTRL
sgDCTD #1
sgDCTD #2
p < 0.0001
F
DCTD
(14%, Cancer Cell Line Encyclopedia, n=961)
Genetic Alteration
Amplification
Deep Deletion
Structural Variant (unknown significance)
Truncating Mutation (unknown significance)
Missense Mutation (unknown significance)
G
Relative cell viabilty (%)
Thymidine concentration (mM)
HAP1
Jurkat E6-1
H
Relative cell viabilty (%)
DAC concentration (μM)
WT #40
KO #23
KO #57
WT #40 +dT
KO #23 +dT
KO #57 +dT
p < 0.0001
p < 0.0001
p < 0.0001
I
Jurkat
Relative cell viabilty (%)
DAC concentration (μM)
sgCTRL
sgCTRL +dT
sgDCTD #2 +dT
sgDCTD #2
p = 0.0679
p < 0.0001
J
Relative cell viabilty (%)
DAC concentration (μM)
sgCTRL
sgTK1 #1
sgTK1 #2
p < 0.0001

© The Author(s)

Figure EV2. Modulating cellular nucleotide pool impacts sensitivity to DAC.

(**A**) Dose–response curves of HAP1 cells transduced with sgCTRL or two different sgRNAs targeting *DCTPP1* (sg*DCTPP1* #1 and #2) upon DAC treatment at indicated concentrations. Cell viability was measured by CellTiter-Glo after 3 days of DAC treatment. Data are presented with ± SEM. Experiments performed in duplicates. The *p* values were determined using nonlinear regression followed by the extra sum-of-squares F test. (**B**) Quantification of γH2A.X immunostaining signal intensity in control and *DCTD*-depleted HAP1 cells after DAC treatment treated with DMSO or DAC (500 nM) for 24 h. Each point represents the mean of one experiment ($N = 2$ experiments; $n > 3700$ cells for each genotype and treatment). A.U., arbitrary unit. (**C**) Immunoblot analysis showing the DCTD protein level in WT and *DCTD* KO clones with HDAC1 as a loading control. (**D**) Dose–response curves of WT and *DCTD* KO HAP1 clones upon DAC treatment at indicated concentrations. Cell viability was measured by CellTiter-Glo after 3 days of DAC treatment. Data are presented with ± SEM ($n = 6$ replicates). The *p* values were determined using nonlinear regression followed by the extra sum-of-squares F test. (**E**) Dose–response curves of Jurkat cells transduced with sgCTRL or two different sgRNAs targeting *DCTD* (sg*DCTD* #1 and #2) upon DAC treatment at indicated concentrations. Cell viability was measured by CellTiter-Glo after 3 days of DAC treatment. Data are presented with ± SEM. Experiments performed in duplicates. The *p* values were determined using nonlinear regression followed by the extra sum-of-squares F test. (**F**) The genetic alteration profile of *DCTD* in cancer cell lines (data was from cBioPortal). (**G**) Dose–response curves of HAP1 and Jurkat cells treated with thymidine at indicated concentrations for 3 days. Cell viability was measured by CellTiter-Glo. Data are presented with ± SEM. Experiments performed in duplicates. (**H**) Dose–response curves of WT and *DCTD* KO HAP1 clones upon DAC treatment at indicated concentrations with or without (w/o) 50 μM thymidine. Cell viability was measured by CellTiter-Glo after 3 days of DAC treatment. Data are presented with ± SEM. Experiments performed in duplicates. The *p* values were determined using nonlinear regression followed by the extra sum-of-squares F test. (**I**) Dose–response curves of Jurkat cells transduced with control or DCTD-targeting sgRNAs upon DAC treatment at indicated concentrations w/o 10 μM thymidine for 3 days. Cell viability was measured by CellTiter-Glo. Data are presented with ± SEM. Experiments performed in duplicates. The *p* values were determined using nonlinear regression followed by the extra sum-of-squares F test. (**J**) Dose–response curves of HAP1 cells transduced with sgCTRL or two different sgRNAs targeting *TK1* (sg*TK1* #1 and #2) upon DAC treatment at indicated concentrations. Cell viability was measured by CellTiter-Glo after 3 days of DAC treatment. Data are presented with ± SEM. Experiments performed in duplicates. The *p* values were determined using nonlinear regression followed by the extra sum-of-squares F test.

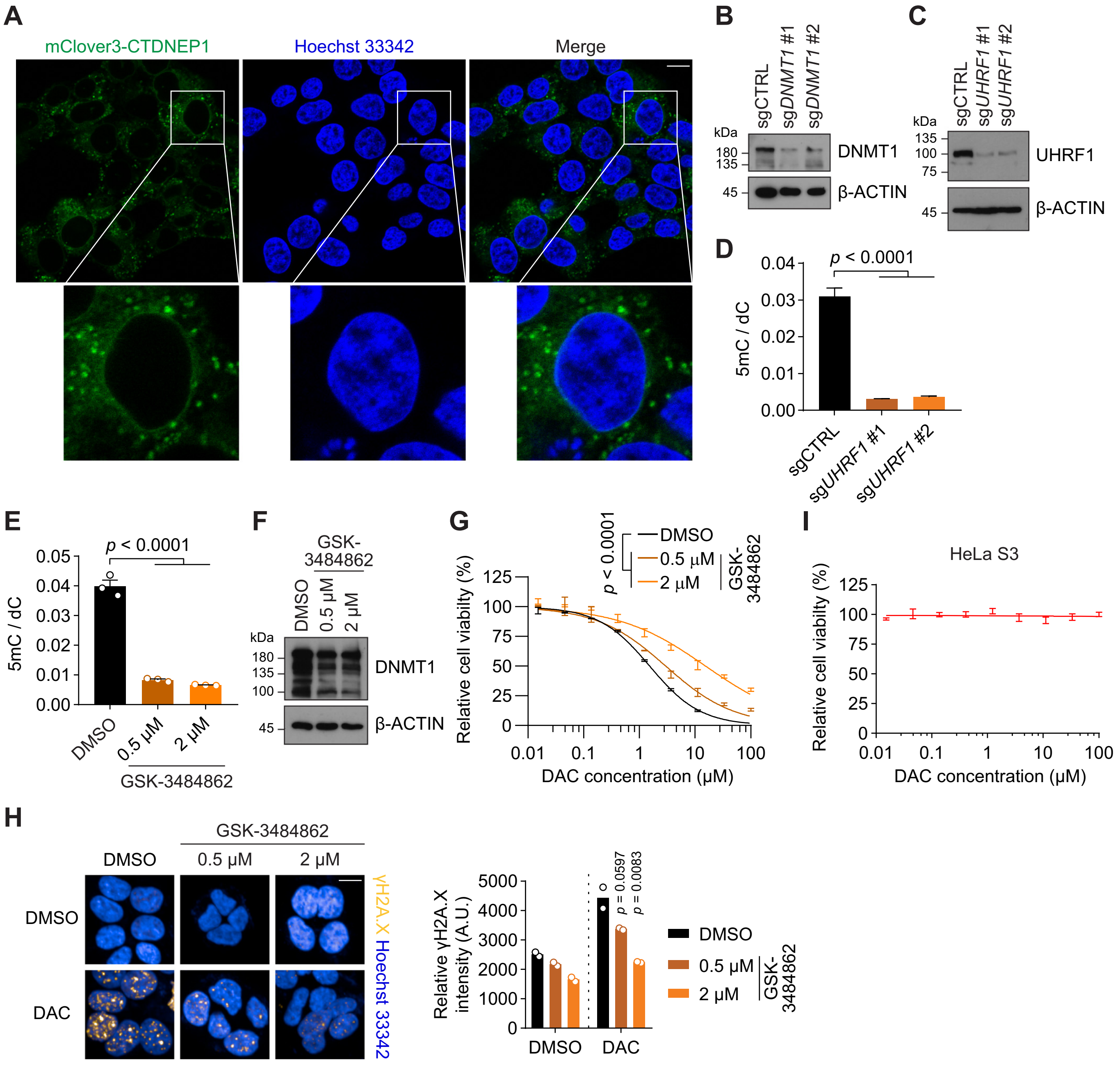
A
mClover3-CTDNEP1
Hoechst 33342
Merge
B
sgCTRL
sgDNMT1 #1
sgDNMT1 #2
kDa
180
135
45
DNMT1
β-ACTIN
C
sgCTRL
sgUHRF1 #1
sgUHRF1 #2
kDa
135
100
75
45
UHRF1
β-ACTIN
D
p < 0.0001
5mC / dC
0.04
0.03
0.02
0.01
0.00
sgCTRL
sgUHRF1 #1
sgUHRF1 #2
E
p < 0.0001
5mC / dC
0.05
0.04
0.03
0.02
0.01
0.00
DMSO
0.5 μM
2 μM
GSK-3484862
F
GSK-3484862
DMSO
0.5 μM
2 μM
kDa
180
135
100
45
DNMT1
β-ACTIN
G
p < 0.0001
DMSO
0.5 μM
2 μM
GSK-3484862
Relative cell viability (%)
125
100
75
50
25
0
0.01
0.1
1
10
100
DAC concentration (μM)
I
HeLa S3
Relative cell viability (%)
125
100
75
50
25
0
0.01
0.1
1
10
100
DAC concentration (μM)
H
GSK-3484862
DMSO
0.5 μM
2 μM
DMSO
DAC
γH2A.X Hoechst 33342
Relative γH2A.X intensity (A.U.)
5000
4000
3000
2000
1000
0
p = 0.0597
p = 0.0083
DMSO
DAC
DMSO
0.5 μM
2 μM
GSK-3484862

 © The Author(s)

Figure EV3. Modulating global DNA methylation level affects the extent of DAC-induced DNA damage.

(**A**) Representative confocal images of mClover3-CTDNEP1 in HAP1 cells. The scale bar is 10 μm. (**B**) Immunoblot analysis showing the DNMT1 protein level in HAP1 cells transduced with either control sgRNA or two different sgRNAs targeting *DNMT1*, with β-ACTIN as a loading control. (**C**) Immunoblot analysis showing the UHRF1 protein level in HAP1 cells transduced with either control sgRNA or two different sgRNAs targeting *UHRF1*, with β-ACTIN as a loading control. (**D**) Percentage of 5mC/dC quantified by LC-MS/MS in genomic DNA isolated from HAP1 cells transduced with control sgRNA or two different sgRNAs targeting *UHRF1*. Data represent the mean ± SD ($n = 3$ technical replicates). The p values were determined using one-way ANOVA followed by Dunnett's multiple comparisons test. (**E**) Percentage of 5mC/dC quantified by LC-MS/MS in genomic DNA isolated from HAP1 cells treated with DMSO or DNMT1 inhibitor GSK-3484862 at indicated concentrations. Each data point represents an independent replicate. Data represent the means ± SEM ($n = 3$ replicates). The p values were determined using one-way ANOVA followed by Dunnett's multiple comparisons test. (**F**) Immunoblot analysis showing the DNMT1 protein level in HAP1 cells treated with DMSO or DNMT1 inhibitor GSK-3484862 at indicated concentrations for 3 days, with β-ACTIN as a loading control. (**G**) Dose–response curves of HAP1 cells pre-treated with DMSO or DNMT1 inhibitor GSK-3484862 at indicated concentrations for 3 days upon DAC treatment. Cell viability was measured by CellTiter-Glo after 3 days of DAC treatment. Data are presented with ± SEM. Experiments performed in duplicates. The p values were determined using nonlinear regression followed by the extra sum-of-squares F test. (**H**) Representative images of γH2A.X immunostaining in HAP1 cells treated with DMSO or DAC (500 nM) for 24 h (left). HAP1 cells were pre-treated with DMSO or DNMT1 inhibitor GSK-3484862 at indicated concentrations for 3 days before DAC treatment. Quantification of γH2A.X immunostaining signal intensity in these cells after DAC treatment (right). Each point represents the mean of one experiment ($N = 2$ experiments; $n > 1800$ cells for each treatment). The p values were determined using one-way ANOVA followed by Dunnett's multiple comparisons test. The scale bar is 10 μm. (**I**) Dose–response curve of HeLa S3 cells upon DAC treatment at indicated concentrations. Cell viability was measured by CellTiter-Glo after 3 days of DAC treatment. Data are presented with ± SEM. Experiments performed in duplicates.

© The Author(s)

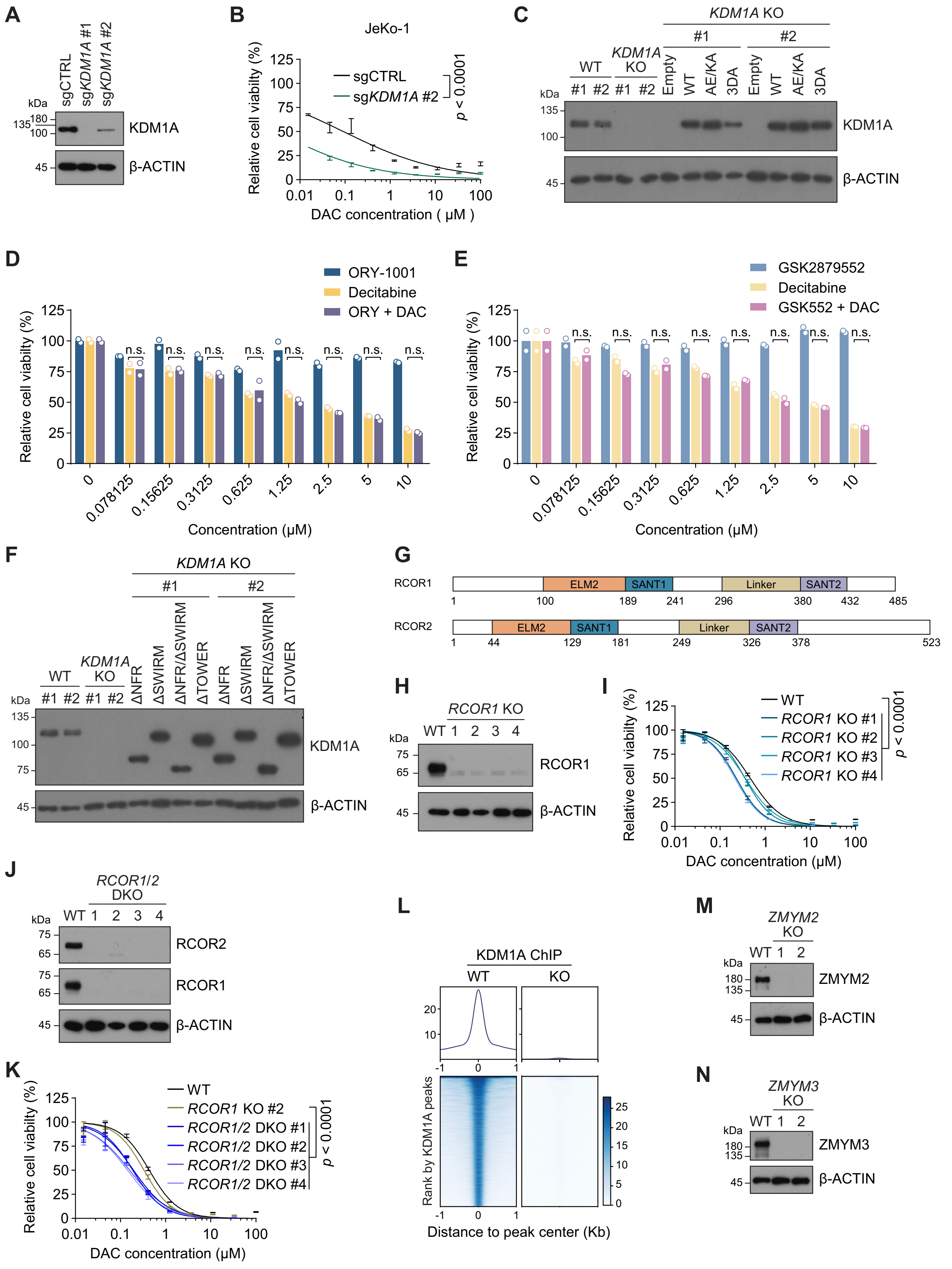
A
sgCTRL
sgKDM1A #1
sgKDM1A #2
kDa
180
135
100
KDM1A
45
β-ACTIN
B
JeKo-1
sgCTRL
sgKDM1A #2
p < 0.0001
Relative cell viability (%)
DAC concentration (μM)
C
KDM1A KO
#1
#2
WT
KDM1A KO
Empty
WT
AE/KA
3DA
KDM1A
β-ACTIN
D
ORY-1001
Decitabine
ORY + DAC
n.s.
Concentration (μM)
E
GSK2879552
Decitabine
GSK552 + DAC
F
KDM1A KO
ΔNFR
ΔSWIRM
ΔNFR/ΔSWIRM
ΔTOWER
G
RCOR1
ELM2
SANT1
Linker
SANT2
RCOR2
H
RCOR1 KO
RCOR1
I
WT
RCOR1 KO #1
RCOR1 KO #2
RCOR1 KO #3
RCOR1 KO #4
DAC concentration (μM)
J
RCOR1/2 DKO
RCOR2
RCOR1
K
RCOR1 KO #2
RCOR1/2 DKO #1
RCOR1/2 DKO #2
RCOR1/2 DKO #3
RCOR1/2 DKO #4
L
KDM1A ChIP
WT
KO
Rank by KDM1A peaks
Distance to peak center (Kb)
M
ZMYM2 KO
ZMYM2
N
ZMYM3 KO
ZMYM3

© The Author(s)

Figure EV4. Not the demethylase activity of KDM1A, but the interactions between KDM1A with RCOR proteins and ZMYM3 contribute to the cellular response to DAC.

(**A**) Immunoblot analysis showing the KDM1A protein level in HAP1 cells transduced with either sgCTRL or two different sgRNAs targeting *KDM1A*. (**B**) Dose–response curves of JeKo-1 cells transduced with control or *KDM1A*-targeting sgRNAs upon DAC treatment at indicated concentration. Cell viability was measured by CellTiter-Glo after 3 days of DAC treatment. Data are presented with ± SEM. Experiments performed in duplicates. The *p* value was determined using nonlinear regression followed by the extra sum-of-squares F test. (**C**) Immunoblot analysis showing the KDM1A protein level in WT and *KDM1A* KO HAP1 clones, and *KDM1A* KO clones complemented with either empty vector, KDM1A-WT, KDM1A-AE/KA, or KDM1A-3DA with β-ACTIN as a loading control. (**D**) Survival analysis of HAP1 cells after treatment with DAC, KDM1A inhibitor ORY-1001, or both at indicated concentrations for 3 days. Cell viability was measured by CellTiter-Glo. Each data point represents one independent experiment. The *p* values were determined using two-way ANOVA followed by Dunnett's multiple comparisons test (n.s., not significant). (**E**) Survival analysis of HAP1 cells after treatment with DAC, KDM1A inhibitor GSK2879552, or both at indicated concentrations for 3 days. Cell viability was measured by CellTiter-Glo. Each data point represents one independent experiment. The *p* values were determined using two-way ANOVA followed by Dunnett's multiple comparisons test. (**F**) Immunoblot analysis showing the KDM1A protein level in WT and *KDM1A* KO HAP1 clones, and *KDM1A* KO clones complemented with either KDM1A-ΔNFR, KDM1A-ΔSWIRM, KDM1A-ΔNFR/ΔSWIRM, or KDM1A-ΔTOWER with β-ACTIN as a loading control. (**G**) Schematic diagrams of the domain structure of full-length RCOR1 and RCOR2. (**H**) Immunoblot analysis showing the RCOR1 protein level in WT and *RCOR1* KO HAP1 clones with β-ACTIN as a loading control. (**I**) Dose–response curves of WT and *RCOR1* KO HAP1 clones upon DAC treatment at indicated concentrations. Cell viability was measured by CellTiter-Glo after 3 days of DAC treatment. Data are presented with ± SEM. Experiments performed in duplicates. The *p* values were determined using nonlinear regression followed by the extra sum-of-squares F test. (**J**) Immunoblot analysis showing the RCOR1 and RCOR2 protein levels in WT and *RCOR1* and *RCOR2* double knockout (DKO) HAP1 clones with β-ACTIN as a loading control. (**K**) Dose–response curves of WT, *RCOR1* KO #2, and *RCOR1/2* DKO HAP1 clones upon DAC treatment at indicated concentration. Cell viability was measured by CellTiter-Glo after 3 days of DAC treatment. Data are presented with ± SEM. Experiments performed in duplicates. The *p* values were determined using nonlinear regression followed by the extra sum-of-squares F test. (**L**) KDM1A ChIP-seq metagene profiles at KDM1A binding peaks in HAP1 WT and *KDM1A* KO clones (upper). Heatmap representation of KDM1A occupancies at KDM1A binding peaks in WT and *KDM1A* KO clones ranked by the intensity of KDM1A ChIP-seq signal (lower). (**M**) Immunoblot analysis showing the ZMYM2 protein level in WT and *ZMYM2* KO HAP1 clones with β-ACTIN as a loading control. (**N**) Immunoblot analysis showing the ZMYM3 protein level in WT and *ZMYM3* KO HAP1 clones with β-ACTIN as a loading control.

© The Author(s)

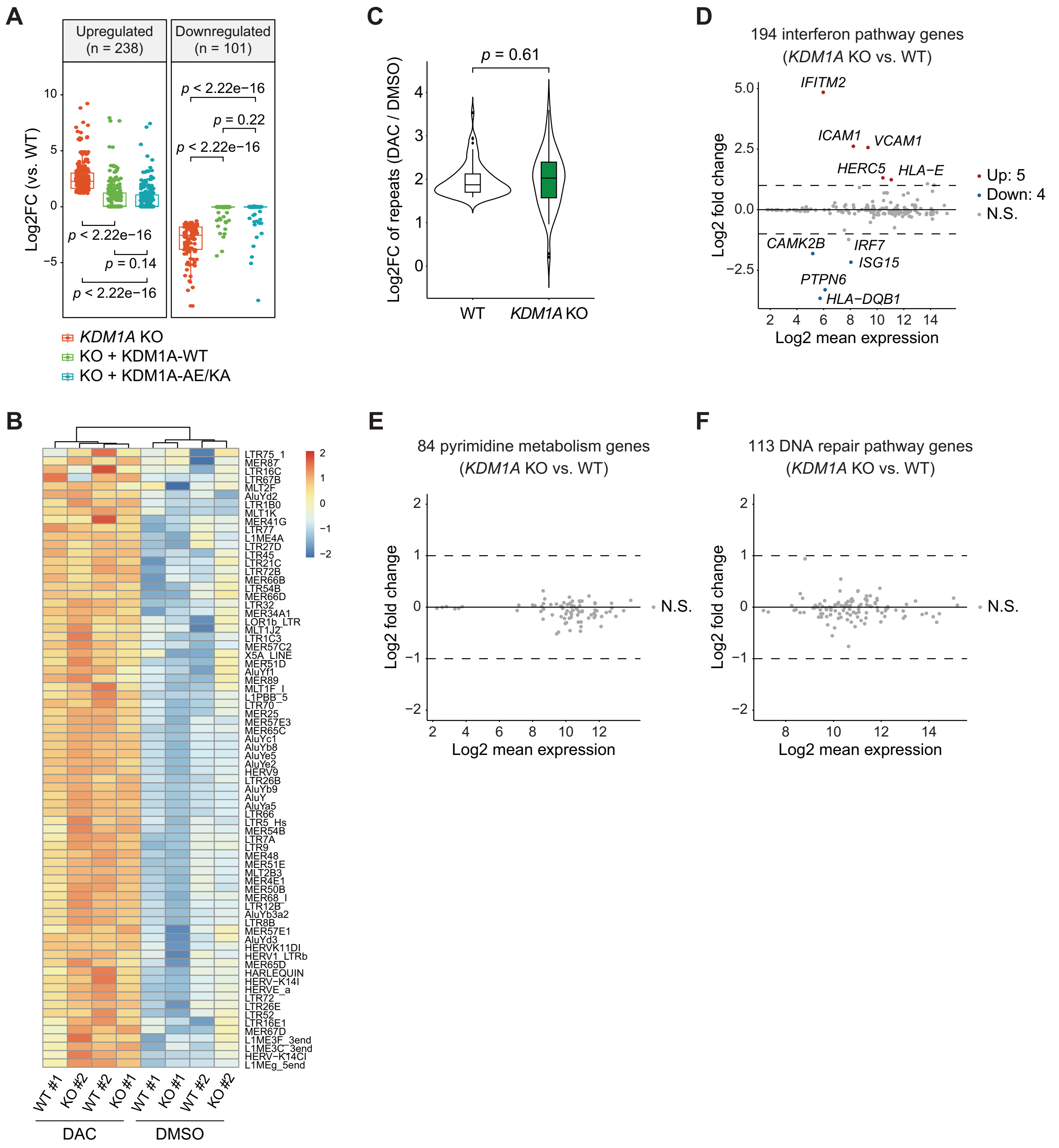
A
Upregulated (n = 238)
Downregulated (n = 101)
Log2FC (vs. WT)
p < 2.22e−16
p = 0.14
p < 2.22e−16
p < 2.22e−16
p = 0.22
p < 2.22e−16
KDM1A KO
KO + KDM1A-WT
KO + KDM1A-AE/KA
B
LTR75_1
MER87
LTR16C
LTR67B
MLT2F
AluYd2
LTR1B0
MLT1K
MER41G
LTR77
L1ME4A
LTR27D
LTR45
LTR21C
LTR72B
MER66B
LTR54B
MER66D
LTR32
MER34A1
LOR1b_LTR
MLT1J2
LTR1C3
MER57C2
X5A_LINE
MER51D
AluYf1
MER89
MLT1F_I
L1PBB_5
LTR70
MER25
MER57E3
MER65C
AluYc1
AluYb8
AluYe5
AluYe2
HERV9
LTR26B
AluYb9
AluY
AluYa5
LTR66
LTR5_Hs
MER54B
LTR7A
LTR9
MER48
MER51E
MLT2B3
MER4E1
MER50B
MER68_I
LTR12B
AluYb3a2
LTR8B
MER57E1
AluYd3
HERVK11DI
HERV1_LTRb
MER65D
HARLEQUIN
HERV-K14I
HERVE_a
LTR72
LTR26E
LTR52
LTR16E1
MER67D
L1ME3F_3end
L1ME3C_3end
HERV-K14CI
L1MEg_5end
WT #1 KO #2 WT #2 KO #1 WT #1 KO #1 WT #2 KO #2
DAC
DMSO
C
p = 0.61
Log2FC of repeats (DAC / DMSO)
WT
KDM1A KO
D
194 interferon pathway genes (KDM1A KO vs. WT)
Log2 fold change
Log2 mean expression
IFITM2
ICAM1
VCAM1
HERC5
HLA−E
CAMK2B
IRF7
ISG15
PTPN6
HLA−DQB1
Up: 5
Down: 4
N.S.
E
84 pyrimidine metabolism genes (KDM1A KO vs. WT)
Log2 fold change
Log2 mean expression
N.S.
F
113 DNA repair pathway genes (KDM1A KO vs. WT)
Log2 fold change
Log2 mean expression
N.S.

 © The Author(s)

Figure EV5. Transcriptome analysis in WT and *KDM1A* KO HAP1 clones.

(**A**) Box plot depicting transcriptional changes of significantly upregulated and downregulated genes upon loss-of-KDM1A could be rescued by complementation with either KDM1A-WT or KDM1A-AE/KA. Box plots are presented with median (horizontal line) and upper and lower quartile boundaries (box range), plus 1.5 times inter-quartile range (whiskers). FC, fold change. The *p* values were determined by Wilcoxon test. (**B**) Heatmap depicting loss-of-KDM1A has no significant effect on the transcriptional activation of retroelements after 500 nM DAC treatment for 3 days. (**C**) Violin plot showing differential expression of retroelements activated by DAC treatment from (**B**) in HAP1 WT and *KDM1A* KO clones. Box plots are presented with median (horizontal line) and upper and lower quartile boundaries (box range), plus 1.5 times inter-quartile range (whiskers). The *p* value was determined by Wilcoxon test ($n = 74$). (**D**) MA-plot showing differential expression of interferon pathway genes after *KDM1A* KO in HAP1 cells. (**E**) MA-plot showing differential expression of pyrimidine metabolism genes after *KDM1A* KO in HAP1 cells. (**F**) MA-plot showing differential expression of DNA repair genes after *KDM1A* KO in HAP1 cells.

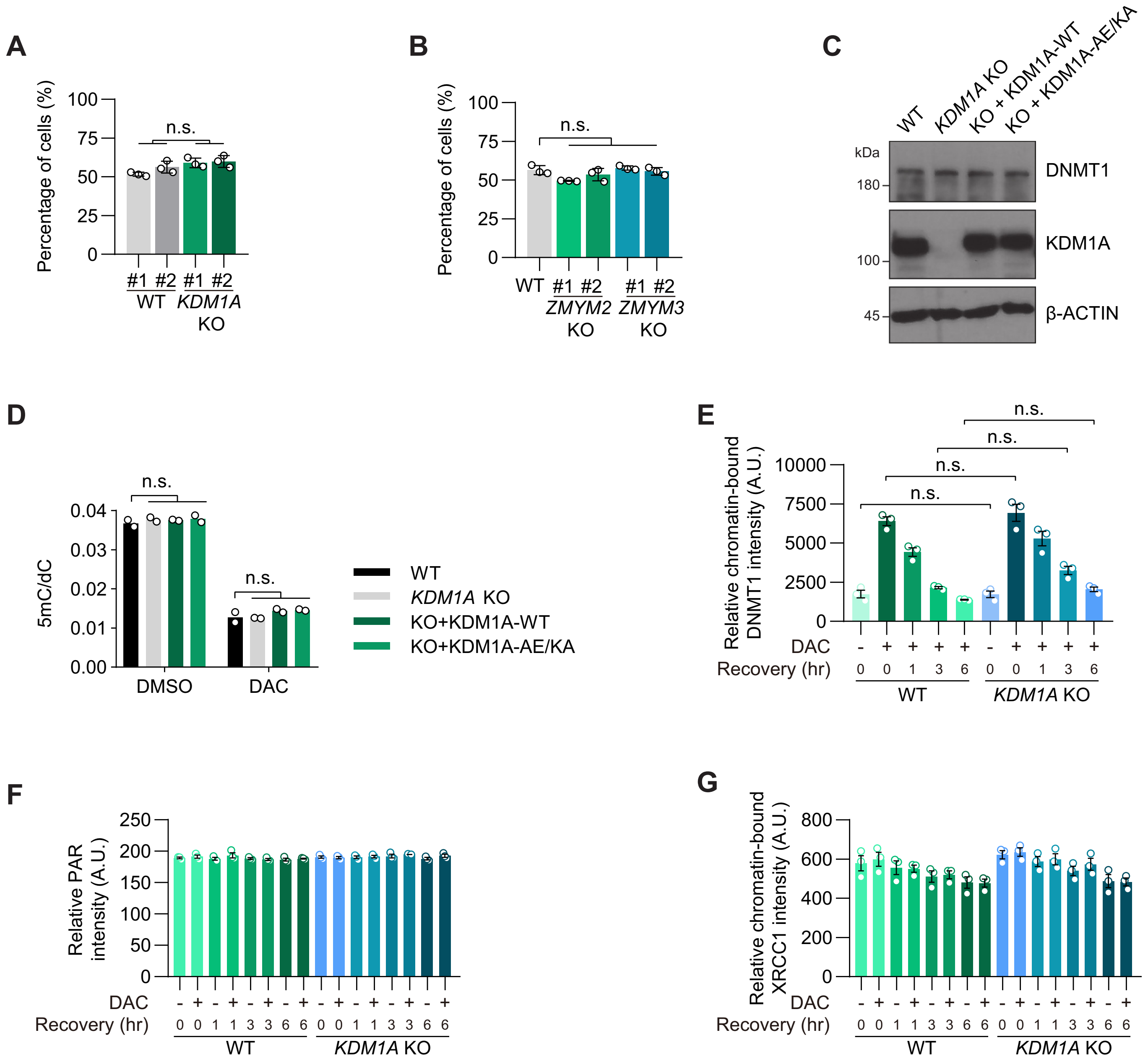

Figure EV6. KDM1A does not affect S-phase progression, global DNA methylation, or the levels of PARylation and XRCC1 in response to DAC pulse.

(**A**) Percentages of cells in S phase in WT and *KDM1A* KO clones. Data are presented as mean ± SEM ($n = 3$ replicates). The p values were determined using one-way ANOVA followed by Sidak's multiple comparisons test. (**B**) Percentages of cells in S phase in WT, *ZMYM2* KO, and *ZMYM3* KO clones. Data are presented as mean ± SEM ($n = 3$ replicates). The p values were determined using one-way ANOVA followed by Sidak's multiple comparisons test. (**C**) Immunoblot analysis showing the DNMT1 protein level in the HAP1 WT, *KDM1A* KO, and *KDM1A* KO clone complemented with either KDM1A-WT or KDM1A-AE/KA, with β-ACTIN as a loading control. (**D**) Percentage of 5mC/dC quantified by LC-MS/MS in genomic DNA isolated from WT and *KDM1A* KO clones, and KDM1A KO clones complemented with either KDM1A-WT or KDM1A-AE/KA treated with DMSO or 500 nM DAC for 3 days. Experiments performed in duplicates. The p values were determined using two-way ANOVA followed by Dunnett's multiple comparisons test. (**E**) DNMT1 protein levels in HAP1 WT and *KDM1A* KO cells at the indicated time points after release from single-round thymidine synchronization, with or without a 30-min DAC (10 μM) pulse. Data represent the means ± SEM ($n = 3$). The p values were determined using one-way ANOVA followed by Sidak's multiple comparisons test. (**F**) PARylation levels in HAP1 WT and *KDM1A* KO cells at the indicated time points after release from single-round thymidine synchronization, with or without a 30-min DAC (10 μM) pulse. Data represent the means ± SEM ($n = 3$). (**G**) Chromatin-bound XRCC1 levels in HAP1 WT and *KDM1A* KO cells at the indicated time points after release from single-round thymidine synchronization, with or without a 30-min DAC (10 μM) pulse. Data represent the means ± SEM ($n = 3$). Source data are available online for this figure.

 © The Author(s)

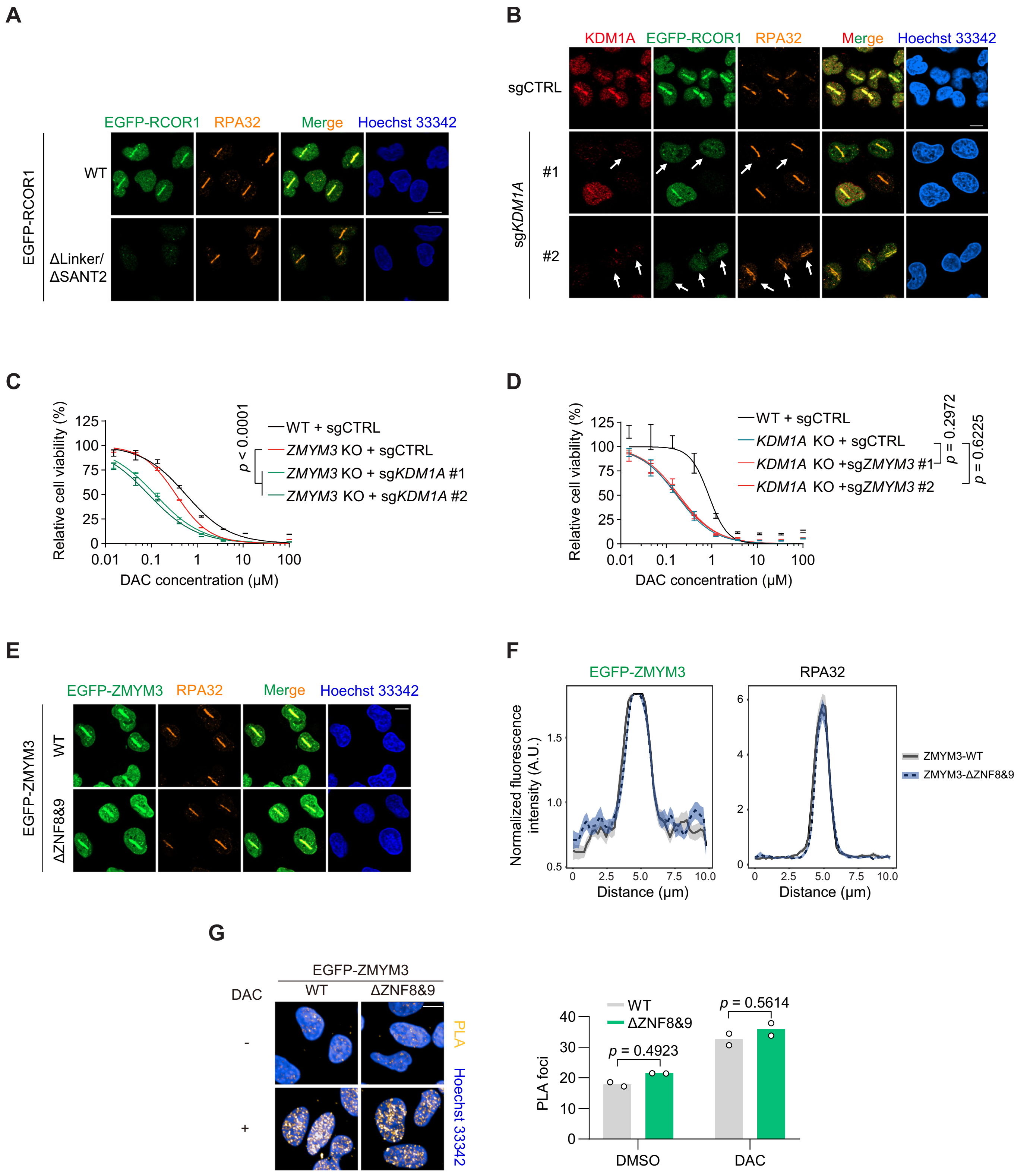
A
EGFP-RCOR1
RPA32
Merge
Hoechst 33342
EGFP-RCOR1
WT
ΔLinker/ ΔSANT2
B
KDM1A
EGFP-RCOR1
RPA32
Merge
Hoechst 33342
sgCTRL
sgKDM1A
#1
#2
C
Relative cell viability (%)
DAC concentration (μM)
p < 0.0001
WT + sgCTRL
ZMYM3 KO + sgCTRL
ZMYM3 KO + sgKDM1A #1
ZMYM3 KO + sgKDM1A #2
D
Relative cell viability (%)
DAC concentration (μM)
WT + sgCTRL
KDM1A KO + sgCTRL
KDM1A KO +sgZMYM3 #1
KDM1A KO +sgZMYM3 #2
p = 0.2972
p = 0.6225
E
EGFP-ZMYM3
RPA32
Merge
Hoechst 33342
EGFP-ZMYM3
WT
ΔZNF8&9
F
EGFP-ZMYM3
RPA32
Normalized fluorescence intensity (A.U.)
Distance (μm)
ZMYM3-WT
ZMYM3-ΔZNF8&9
G
EGFP-ZMYM3
DAC
WT
ΔZNF8&9
-
+
PLA
Hoechst 33342
WT
ΔZNF8&9
p = 0.5614
p = 0.4923
PLA foci
DMSO
DAC

Figure EV7. ZMYM3 is partially responsible for the recruitment of KDM1A to DNA damage sites.

(**A**) Representative images showing the Linker and SANT2 domains of RCOR1 were required for its recruitment to laser-induced damage sites. EGFP-tagged WT or ΔLinker/ΔSANT2 mutant RCOR1 were expressed in U2OS cells. RPA32 was used as a DNA DSB marker. The scale bar is 10 µm. (**B**) Representative images showing the recruitment of EGFP-tagged RCOR1 to laser-induced damage sites in U2OS cells transduced with control sgRNA or two different sgRNAs targeting *KDM1A*. Arrows indicate *KDM1A*-depleted U2OS cells. RPA32 was used as a DNA DSB marker. The scale bar is 10 µm. (**C**) Dose–response curves of HAP1 WT and *ZMYM3* KO cells transduced with control sgRNA (sgCTRL) or two different sgRNAs targeting *KDM1A* (sg*KDM1A* #1 and #2) upon DAC treatment at indicated concentrations. Cell viability was measured by CellTiter-Glo after 3 days of DAC treatment. Data are presented with ± SEM. Experiments performed in duplicates. The *p* values were determined using nonlinear regression followed by the extra sum-of-squares F test. (**D**) Dose–response curves of HAP1 WT and *KDM1A* KO cells transduced with control sgRNA (sgCTRL) or two different sgRNAs targeting *ZMYM3* (sg *ZMYM3* #1 and #2) upon DAC treatment at indicated concentrations. Cell viability was measured by CellTiter-Glo after 3 days of DAC treatment. Data are presented with ± SEM. Experiments performed in duplicates. The *p* values were determined using nonlinear regression followed by the extra sum-of-squares F test. (**E**) Representative images showing the recruitment of EGFP-tagged wild-type ZMYM3 or ΔZNF8&9 mutant to laser-induced damage sites in U2OS cells. RPA32 was used as a DNA DSB marker. The scale bar is 10 µm. (**F**) Quantification for profiles of EGFP-tagged ZMYM3 and RPA32 immunostaining signals along the lines across damage stripes in (**E**). The relative signal intensities were normalized to the mean of U2OS cells expressing EGFP-tagged wild-type ZMYM3. Data represent mean ± SEM ($n = 24$ cells for ZMYM3-WT; $n = 26$ cells for ZMYM3-ΔZNF8&9). (**G**) Representative images (left) of PLA results between EGFP and γH2A.X in U2OS cells expressing either EGFP-tagged wild-type ZMYM3 or ΔZNF8&9 mutant 24 h after release from single-round thymidine synchronization, with or without a 30-min DAC (10 µM) pulse. Quantification of PLA foci number (right) in these cells. Each point represents the mean of one experiment ($N = 2$ experiments; $n > 5000$ cells for each condition). The *p* values were determined using one-way ANOVA followed by Sidak's multiple comparisons test. The scale bar is 10 µm.

 © The Author(s)
